# Shaping of a Reactive Manganese Catalyst Enables Access to Polyfunctionalized Cyclohexanes via Enantioselective C(*sp^3^*)─H Bond Oxidation of 1,3‐*meso* Diethers

**DOI:** 10.1002/anie.202507755

**Published:** 2025-06-01

**Authors:** Andrea Palone, Arnau Call, Aleria Garcia‐Roca, Josep M. Luis, Matthew S. Sigman, Massimo Bietti, Cristina Nevado, Miquel Costas

**Affiliations:** ^1^ Institut de Química Computacional i Catàlisi (IQCC) and Departament de Química Universitat de Girona Campus Montilivi Girona Catalonia E‐17071 Spain; ^2^ Department of Chemistry University of Utah Salt Lake City Utah 84112 USA; ^3^ Dipartimento di Scienze e Tecnologie Chimiche Università “Tor Vergata” Via della Ricerca, Scientifica, 1 Rome I‐00133 Italy; ^4^ Department of Chemistry University of Zurich Winterthurerstrasse 190 Zurich CH 8057 Switzerland

**Keywords:** Bioinspired catalysis, Desymmetrization, Enantioselective C─H oxidation, Homogeneous catalysis, Manganese

## Abstract

Chiral polyoxygenated cyclohexanes are valuable constituents of biologically relevant products. Herein, we report a protocol for the direct access to these scaffolds via site‐ and enantioselective non‐directed oxidation of cyclohexyl‐3,5‐*meso* diethers using aqueous H₂O₂. Structural shaping of a highly reactive chiral Mn‐oxo species, achieved through the combination of a sterically encumbered ligand and a bulky carboxylic acid, promotes a precise fit of the substrate within the catalyst pocket, which translates into exceptional enantioselectivity (up to >99% ee). Computational studies reveal that C─H oxidation proceeds via an initial hydrogen atom transfer, followed by electron transfer, leading to the formation of a chiral cationic intermediate. The resulting desymmetrized 3‐methoxycyclohexanone products serve as valuable intermediates for the synthesis of bioactive cores, as they can undergo orthogonal chemical modifications to enable further structural diversification.

## Graphical abstract

Structural shaping of a highly reactive chiral Mn‐oxo species translates into exceptionally enantioselective C─H oxidation catalyst of cyclohexyl 1,3*‐meso* diethers (up to >99% ee) leading to polyfunctionalized cyclohexanes.

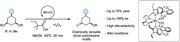

## Introduction

The enantioselective functionalization of C(*sp^3^*)─H bonds represents a powerful strategy for simultaneously generating structural complexity and imparting stereocontrol from readily available starting materials.^[^ ^1^, ^2^, ^3^, ^4^ ^]^ Among the many strategies, enantioselective hydrogen atom transfer (HAT) reactions are attracting increasing interest. Relevant, although limited examples, include the use of optically active amine‐boryl radicals in the context of kinetic resolutions,^[^ ^5^, ^6^, ^7^, ^8^ ^]^ and more recently, chiral amines for enantioselective diol epimerization and C─C bond‐forming reactions.^[^ ^9^ ^]^ In this context, enantioselective C(*sp^3^*)─H oxidations are particularly appealing, as oxygenated hydrocarbon frameworks are ubiquitous constituents of biologically relevant products, and chirality is a function‐defining property in numerous biological processes.^[^ ^10^, ^11^, ^12^, ^13^, ^14^ ^]^ The standing limitation in the development of these transformations is the need for reagents capable of oxidizing C(*sp^3^*)─H bonds not only in a site‐ but also in an enantioselective manner.^[^ ^15^ ^]^ These reagents must overcome the challenges posed by the presence of multiple non‐equivalent C(*sp^3^*)─H bonds in most organic molecules, as well as their inherent low reactivity.^[^ ^16^ ^]^

**Figure 1:**
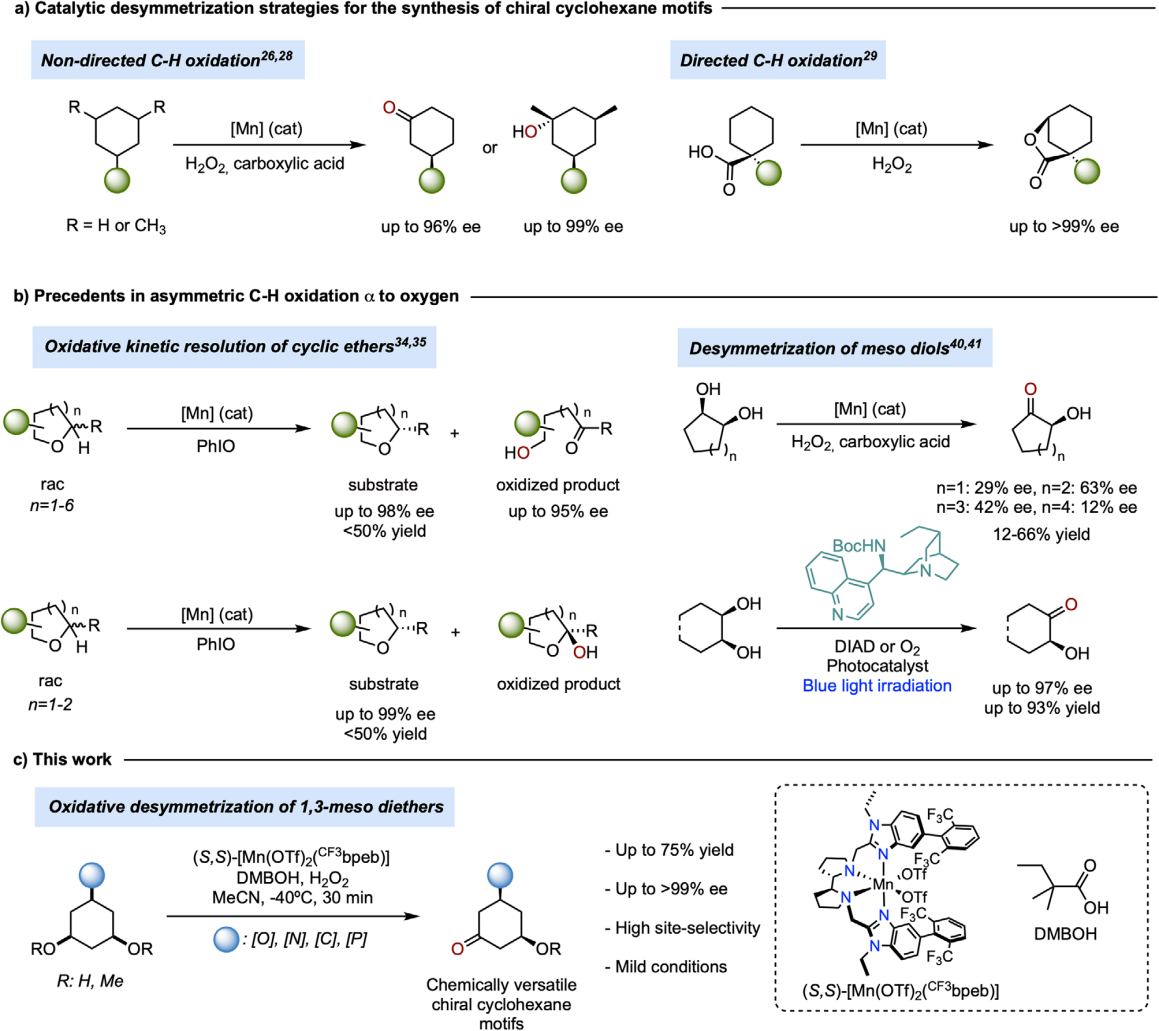
a) Selected precedents for enantioselective synthesis of cyclohexane motifs via C─H bond oxidation. b) Precedents in enantioselective oxidation of C─H bonds adjacent to oxygen. DIAD: diisopropyl azodicarboxylate. c) Current work.

The synergistic combination of chiral manganese complexes and carboxylic acid co‐ligands provides a promising path to address these challenges. These systems engage in enzyme‐like peroxide activation mechanisms to generate high‐valent manganese‐oxo carboxylato species (i.e., [Mn^V^O(OCOR)(N_4_L)]^2+^, where OCOR stands for carboxylate co‐ligand and N_4_L stands for tetradentate ligand). These species act as powerful oxidants capable of stereoretentively oxygenating strong C(*sp^3^*)─H bonds through an initial HAT that, in some cases, may be followed by an electron transfer (ET) from the carbon‐centered radical leading to a cationic intermediate.^[^ ^17^, ^18^, ^19^, ^20^, ^21^, ^22^, ^23^, ^24^ ^]^ Because the HAT step proceeds enantioselectively, it enables the desymmetrization of substrates generating multiple stereocenters in a single step thus yielding stereochemically enriched molecules.^[^ ^25^ ^]^ Based on this concept, our group has recently described manganese catalysts that unlock highly enantioselective oxygenations of non‐activated, strong C(*sp^3^*)─H bonds, such as the ketonization of *N*‐cyclohexyl amides,^[^ ^26^, ^27^ ^]^ the hydroxylation of tertiary C─H bonds in 3,5‐dimethylcyclohexane derivatives,^[^ ^28^ ^]^ and the *γ*‐lactonization of carboxylic acids.^[^ ^29^, ^30^, ^31^ ^]^ By desymmetrizing simple aliphatic derivatives, these reactions provide access to value‐added chiral oxygenated products, thereby expanding the portfolio of chiral pool building blocks (Figure 1a).

Building on their ability to selectively oxidize strong and non‐activated C(*sp^3^*)─H bonds, we reasoned that these catalysts would also react with C─H bonds adjacent to an oxygen atom, which are hyperconjugatively activated toward HAT.^[^ ^32^, ^33^ ^]^ While the oxidation of such bonds is readily achieved by various radical and radical‐like oxidants, enantioselective versions of these transformations are limited.^[^ ^34^, ^35^, ^36^, ^37^, ^38^, ^39^ ^]^ Key examples include the kinetic resolution of racemic tetrahydrofurans and tetrahydropyrans through C(*sp^3^*)─H oxidation at activated positions (up to 99% ee), and the oxidative desymmetrization of cyclic *cis*‐1,2‐diols, although in this case, enantioselectivities do not exceed 63% ee (Figure 1b).^[^ ^40^ ^]^ Most notable is the recent report of Phipps and co‐workers describing a photocatalytic enantioselective HAT‐initiated oxidation of *meso*‐diols mediated by a cinchona alkaloid‐derived catalyst.^[^ ^41^ ^]^ In considering high‐valent manganese‐oxo species, a fundamental challenge exists to achieve high enantio‐discrimination for this substrate class. Specifically, tertiary C─H(OR) bonds have bond dissociation energies (BDEs) that are ∼4 kcal·mol^−1^ lower than those of nonactivated tertiary C─H bonds. Considering that HAT reactions generally follow linear activation energy versus driving force correlations, it can be anticipated that for the weaker C─H(OR) bonds, HAT will proceed with lower activation barriers and earlier transition states.^[^ ^42^ ^]^ In this scenario, achieving energy differences that can induce high enantio‐discrimination is notoriously challenging. One potential solution is to design the catalysts in a manner that allows control over the access of the targeted C─ H(OR) bond to the reactive site. This approach is reminiscent of mechanisms found in iron‐dependent enzymes, where selectivity is dictated by the substrate's approach and orientation relative to the reactive iron‐oxo species, which can be fine‐tuned by mutating specific protein residues in the second coordination sphere of the enzyme.^[^ ^43^, ^44^, ^45^, ^46^ ^]^

Building on these ideas, herein we describe the development of a manganese catalyst that, in combination with hydrogen peroxide and a sterically hindered alkanoic acid, is able to promote the desymmetrization of *meso‐*3,5‐dioxygenated cyclohexanes via enantioselective C(*sp^3^*)─H oxidation (Figure 1c). The reaction transforms readily available precursors into densely oxygenated chiral scaffolds setting, in a single step, two stereocenters and forming a carbonyl group that can be further manipulated orthogonally, offering multiple opportunities for synthetic elaboration. This work comprises a detailed catalyst optimization campaign that was guided by analysis of the shape of the catalytic pocket around the putative high‐valent manganese‐oxo carboxylato species. The resulting optimized catalyst system facilitates reactions with exceptionally low catalyst loadings, under mild conditions, and within short reaction times, efficiently producing chiral functionalized cyclohexanes in up to 75% isolated yield and outstanding enantioselectivity (up to >99% ee). Computational studies reveal that the high site‐ and enantioselectivity arise from an enzyme‐like positioning of a specific C─H bond of the substrate into a highly structured chiral active site in the key enantioselective HAT step.

## Results and Discussion

**Figure 2:**
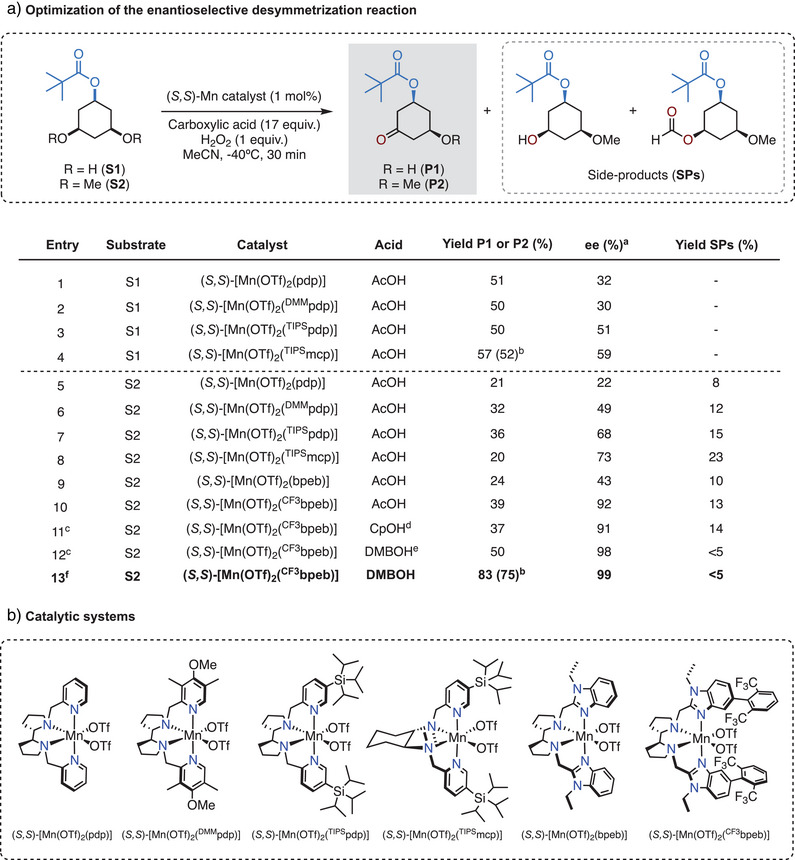
a) Catalyst and co‐ligand optimization in the desymmetrization of esters **S1** and **S2**. ^a^ ee values determined by GC analysis. ^b^ Yield of the isolated product. ^c^ Using 3 equiv. of H_2_O_2_. ^d^ Cyclopropanecarboxylic acid. ^e^ 2,2‐Dimethylbutanoic acid. ^f^ Using 2 mol% catalyst and 7 equiv. of H_2_O_2_. b) Manganese catalysts used in this study. See SI for further details on the screening of different carboxylic acids and manganese catalysts, as well as different substrate OR groups.

Leveraging our recent work on the enantioselective oxidation of 3,5‐dimethyl cyclohexane derivatives,^[^ ^28^ ^]^ we focused on the desymmetrization of *cis*‐3,5‐dihydroxycyclohexyl pivalate (**S1**). Initial optimization was conducted using 1 mol% of various chiral manganese catalysts, 1 equivalent of H_2_O_2_ (delivered over 30 min via syringe pump), in MeCN at −40 °C, in the presence of 17 equivalents of acetic acid (AcOH). Under these conditions, oxidation of **S1** using the (*S,S*)‐[Mn(OTf)_2_(pdp)] (OTf = CF_3_SO_3_^−^, pdp = *N,N’*‐bis(2‐pyridylmethyl)‐2,2′‐bipyrrolidine) furnished *cis*‐3‐hydroxy‐5‐oxocyclohexyl pivalate (**P1**) in 51% yield and moderate 32% ee. The product derives from hydroxylation at one of the tertiary C─H(OH) bonds to generate geminal diol intermediate, which subsequently undergoes water release (Figure 2a, entry 1). Changing to an electron‐rich catalyst (*S,S*)‐[Mn(OTf)_2_(^DMM^pdp)] (^DMM^pdp = *N,N’*‐bis(4‐methoxy‐3,5‐dimethyl‐2‐pyridylmethyl)‐2,2′‐bipyrrolidine) did not improve the reaction outcome (entry 2). Notably, the introduction of bulky *tris*‐isopropylsilyl (TIPS) groups on the pyridine ligand (i.e., (*S,S*)‐[Mn(OTf)_2_(^TIPS^pdp)]) and modification of the catalyst chiral diamine backbone (i.e., (*S,S*)‐[Mn(OTf)_2_(^TIPS^mcp)]; mcp = *N,N’*‐dimethyl *N,N’*‐bis(2‐pyridylmethyl‐1,2‐*trans*‐diaminocyclohexane) led to slightly increased yields of **P1** (50–57%) and enhanced enantioselectivities (51–59% ee) (entries 3–4). Previous examples of manganese‐catalyzed oxidation of secondary alcohols have demonstrated that stereoselectivity is primarily governed by substrate binding to the catalyst, which places the targeted α‐C─H bonds near the metal oxo unit.^[^ ^36^, ^47^, ^48^, ^49^, ^50^ ^]^ In this scenario, the moderate enantioselectivities observed in the oxidation of **S1** likely stem from competitive binding of the two enantiotopic hydroxyl groups, which can limit enantiodiscrimination.^[^ ^51^ ^]^ Accordingly, we questioned whether converting the OH groups into the corresponding methyl ethers (OCH_3_) would improve enantioselectivity.

Oxidation of *cis*‐3,5‐dimethoxycyclohexyl pivalate (**S2**) using (*S,S*)‐[Mn(OTf)_2_(pdp)] catalyst provided *cis*‐3‐methoxy‐5‐oxocyclohexyl pivalate (**P2**) in moderate yield (21%) and low enantioselectivity (22% ee) (entry 5). Interestingly, in contrast to **S1**, when increasing the catalyst size, the oxidation of **S2** showed significant improvements in enantioselectivity: 49, 68, and 73% ee were obtained using (*S,S*)‐[Mn(OTf)_2_(^DMM^pdp)], (*S,S*)‐[Mn(OTf)_2_(^TIPS^pdp)], and (*S,S*)‐[Mn(OTf)_2_(^TIPS^mcp)], respectively (entries 6–8). However, these reactions also generate substantial amounts of side products (**SPs** in Figure 2), which arise from competitive primary C─H bond oxidation at the OCH_3_ groups (see detailed analysis in the SI). The presence of **SPs** demonstrates competition between the weaker but sterically congested tertiary C─H bonds at C_3_ and C_5_ and the stronger yet more accessible and statistically favored primary C─H bonds. This compromised chemoselectivity contrasts with the single product observed in the oxidation of 3,5‐dimethyl‐cyclohexanes,^[^ ^28^ ^]^ a feature that reflects the weaker nature of the ethereal α‐C─H bonds and their increased susceptibility to oxidation.

**Figure 3:**
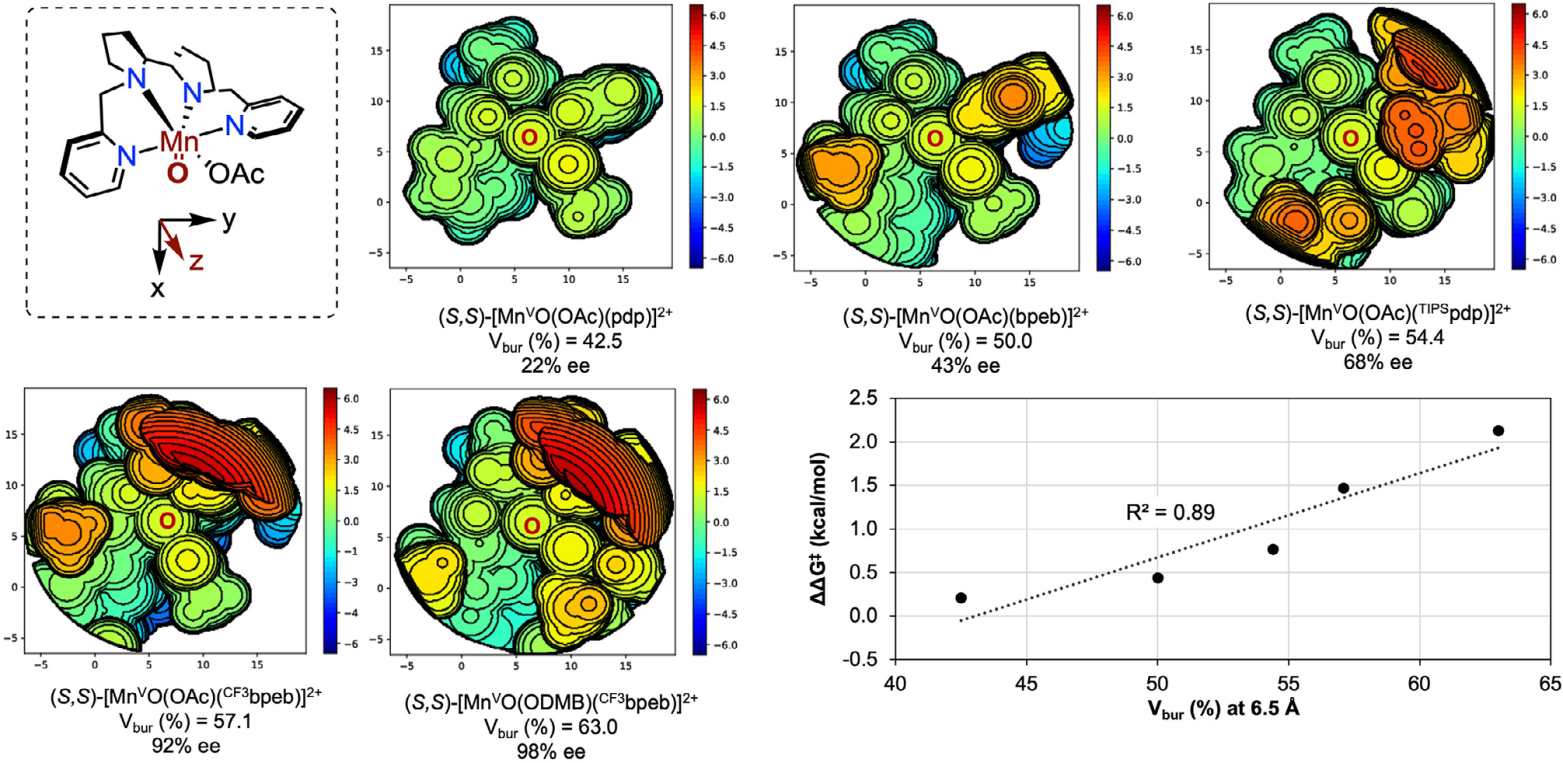
Calculated steric maps of the most stable energy conformer obtained under the applied structural constraints of [Mn^V^O(OCOR)(N_4_L)]^2+^ species visualized by *SambVca* application^[^ ^55^ ^]^ at a radius of 6.5 Å with 0.05 mesh (See SI for details). The map is centered on the oxo unit aligned to the Mn═O vector (*z* axis pointing toward the reader). The plot shows the linear correlation between V_bur_ (%) and enantioselectivity (in ΔΔG^‡^) of the different [Mn^V^O(OCOR)(N_4_L)]^2+^ species.

To identify the factors that govern the enantioselectivity in the non‐directed oxidation of **S2**, we conducted buried volume (V_bur_ (%)) analysis^[^ ^52^ ^]^ at the oxo unit of the catalytically active species responsible for HAT.^[^ ^17^ ^]^ For this purpose, the structural and dynamic behavior of [Mn^V^O(OCOR)(N_4_L)]^2+^ was examined using the Conformer‐Rotamer Ensemble Sampling Tool (CREST) software at the GFNFF‐xTB level, applying several structural constraints, including the O···O distance (See SI for conformational search details).^[^ ^53^ ^]^ The lowest energy conformation was selected for analysis.^[^ ^53^ ^]^ As shown in Figure 3, the increase in enantioselectivity from (*S,S*)‐[Mn(OTf)_2_(pdp)] to (*S,S*)‐[Mn(OTf)_2_(^TIPS^pdp)] correlates with an increase of V_bur_ from 42.5 to 54.4%, which can be reasonably attributed to the restriction of the cone trajectories available for **S2** to reach the oxo unit. Building on this concept, we hypothesized that further increasing the V_bur_ (%) by redesigning the ligand framework could enhance enantioinduction. To test this hypothesis, we considered substituting pyridines with the larger *N*‐ethylbenzimidazoles.^[^ ^54^ ^]^ Using the (*S,S*)‐[Mn(OTf)_2_(bpeb)] catalyst (V_bur_ = 50.0%) results in a notable increase in enantiodiscrimination (43% ee, entry 9) compared to the (*S,S*)‐[Mn(OTf)_2_(pdp)] analog (22% ee, entry 5) without impacting the yield of the desired product **P2**. To further increase V_bur_ (%) and leveraging the observation that steric hindrance on the pyridine ring positively impacts the stereochemical outcome, we introduced an aryl group at position 5 of the benzimidazole ring. Gratifyingly, the sterically encumbered (*S,S*)‐[Mn(OTf)_2_(^CF3^bpeb)] catalyst (V_bur_ = 57.1%) increased the yield of the desired product **P2** (39%) while affording outstanding enantioselectivity (92% ee) (entry 10). Capitalizing on the improved spatial definition around the catalytic center, a further increase in V_bur_ up to 63.0% determined by the use of the bulky 2,2‐dimethylbutanoic acid (DMBOH) co‐ligand in place of acetic acid, improved enantioselectivity to 98%, accompanied by a satisfactory **P2** yield of 50% (entry 12). Notably, this increase in yield can be attributed to a significant reduction in the formation of **SPs** (<5% yield) (See Table S4 for details), resulting in a convergent and efficient tertiary C─H bond oxidation. Further optimization of the catalyst and H_2_O_2_ loadings led to the formation of **P2** in a synthetically useful isolated yield (75%) and outstanding enantioselectivity (99% ee) (entry 13). The catalyst evolution guided by V_bur_ (%) analysis suggests that the increase in enantioinduction is a result of the reduced available volume within the chiral catalyst pocket, evidenced by the satisfactory linear correlation between V_bur_ (%) and enantioselectivity (*R* ^2^ = 0.89) (Figure 3).

## Computational Analysis

**Figure 4:**
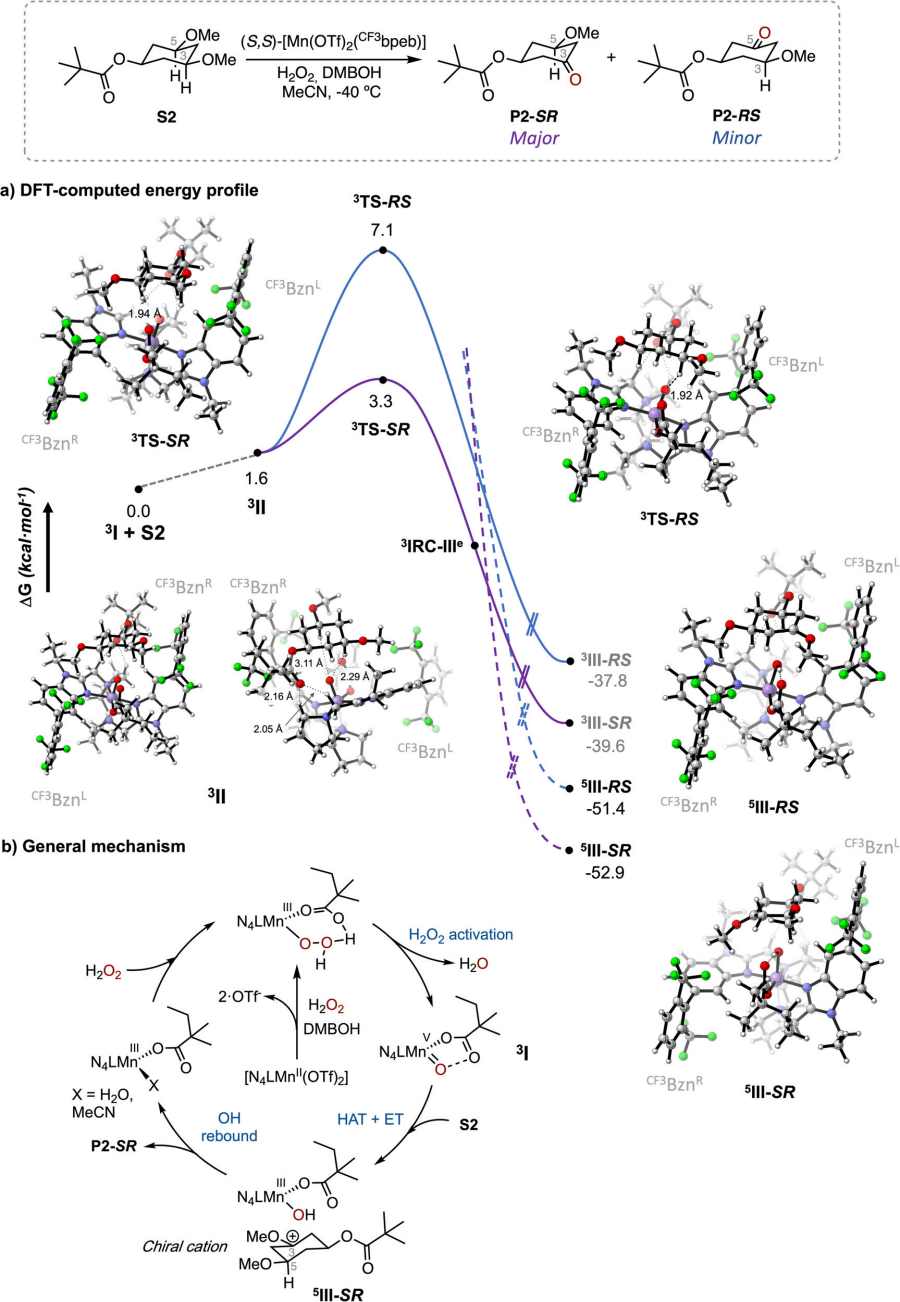
a) Gibbs energy profiles at the B3LYP‐GD3BJ/def2‐TZVPP/SMD(MeCN)//B3LYP‐GD3BJ/6–31G(d,p)(Mn‐LANL2DZ) level of theory for HAT from the C_3_─H (**^3^TS‐*SR***) and C_5_─H (**^3^TS‐*RS***) bonds of **S2** to the Mn‐oxo carboxylato species generated from (*S,S*)‐[Mn(OTf)_2_(^CF3^bpeb)], H_2_O_2_, and DMBOH. The ΔG energies have been computed assuming a temperature of 233.15 K and a concentration of 0.125 M for **S2**. The spin state of each species is denoted by the superscript. Interatomic distances are in Å. Energies in kcal·mol^−1^. b) Proposed mechanism for the Mn‐catalyzed desymmetrization of **S2**, featuring the formation of a chiral cationic intermediate.

The full catalytic cycle proposed for the oxidation of **S2** using the (*S,S*)‐[Mn(OTf)_2_(^CF3^bpeb)] catalyst in combination with DMBOH is displayed in Figure 4b. The C‐H oxidizing species is proposed to be a (*S,S*)‐[Mn^V^O(ODMB)(^CF3^bpeb)]^2+^ (**^3^I**) intermediate formed via a carboxylic acid assisted heterolytic cleavage of a (*S,S*)‐[Mn^III^OOH(DMBOH)(^CF3^bpeb)]^2+^ precursor.^[^ ^17^, ^18^, ^19^, ^20^, ^21^, ^22^ ^]^ To further elucidate the factors that govern the outstanding levels of site‐ and enantioselectivity in the oxidation of **S2** using the (*S,S*)‐[Mn(OTf)_2_(^CF3^bpeb)] catalyst in combination with DMBOH, we conducted density functional theory (DFT) calculations on the C─H cleavage step of the reaction. Considering that the enantio‐determining step for the generation of **P2** is HAT from the tertiary C(*sp^3^*)─H bond, we calculated the free energy profile for the abstraction of the enantiotopic C_3_─H and C_5_─H bonds [leading to the major (**P2*‐SR***) and minor (**P2*‐RS***) enantiomers, respectively]^[^ ^56^ ^]^ by the (*S,S*)‐[Mn^V^O(ODMB)(^CF3^bpeb)]^2+^ intermediate (**^3^I**) via the corresponding α‐OCH_3_ carbon radicals (Figure 4a). We initially evaluated all possible conformations of the reactant complexes, focusing on those where the enantiotopic tertiary C─H bonds are oriented toward the Mn‐oxo unit. The most representative conformations were optimized at the DFT (B3LYP) level using the LANL2DZ basis set and the corresponding pseudopotential for Mn, along with 6–31G(d,p) for all other atoms. Grimme's GD3BJ algorithm was applied for dispersion corrections.^[^ ^57^, ^58^ ^]^ The electronic structures were further refined by B3LYP‐GD3BJ/def2‐TZVPP/SMD(MeCN) single‐point energy corrections. Our analysis of the various reactant complexes revealed that the resting‐state manganese complex can adopt four primary conformations for both C_3_─H and C_5_─H abstractions. Among these, the triplet state (i.e., S = 1) reactant complex **^3^II** was found to be energetically favored for both enantiotopic tertiary C─H bonds, highlighting the highly structured nature of the catalyst pocket that ensures precise substrate positioning, a key factor in achieving the high site‐ and enantioselectivity observed.

The reactant complex **^3^II** positions the pivalate substituent opposite to the DMBO co‐ligand, while the 3,5‐dimethoxycyclohexyl framework is accommodated within the space defined by the two bulky benzimidazole arms (^CF3^Bzn, referred to as ^CF3^Bzn^L^ and ^CF3^Bzn^R^ in Figure 4a). The ^CF3^Bzn groups impose large steric barriers on either side of the catalyst, shaping its pocket. Additionally, the lone pairs of the ester carbonyl group of **S2** interact with the methylenic α‐C─H bond adjacent to the nitrogen of the benzimidazole arm and another α‐C─H bond adjacent to the nitrogen of the chiral pyrrolidine ring of the ligand backbone. This interaction forms a 6‐membered ring‐like structure, significantly stabilizing the reactant species (Figure 4a, **^3^II** views). Similar stabilizing interactions have been previously reported in pyridine‐based manganese catalysts.^[^ ^28^, ^59^ ^]^ Rotating the pivalate group of **S2** suppresses this interaction resulting in a >11 kcal·mol^−1^ free energy increase, as calculated at the B3LYP‐GD3BJ/6–31G(d,p)(Mn‐LANL2DZ) level of theory. This underscores the importance of proper substrate positioning for asymmetric C─H hydroxylation.

Through these interactions, the **^3^II** complex promotes HAT from C_3_─H via the triplet transition state **^3^TS‐*SR***, which features a low Gibbs energy barrier of 3.3 kcal·mol^−1^ (Figure 4a). In contrast, the alternative transition state for C_5_─H cleavage (**^3^TS‐*RS***) leading to the ***RS*‐P2** enantiomer exhibits a significantly higher Gibbs energy barrier of 7.1 kcal·mol^−1^. Additionally, the computed HAT activation barrier aligns well with literature values for the oxidation of activated C─H bonds by Mn oxo species,^[^ ^60^ ^]^ and is slightly lower than those reported for HAT in 3,5‐dimethyl‐cyclohexane derivatives (3.8 kcal·mol^−1^).^[^ ^28^ ^]^ The computed lengths of the C_3_─H and O─H bonds that are cleaved and formed in **^3^TS‐*SR*** are 1.11 and 1.94 Å, respectively, indicating an early transition state.

The IRC path connects the **^3^TS‐*SR*** transition state to a transient species corresponding to the product of a canonical HAT (**^3^IRC‐III^e^** in Figure 4a) that exhibits a spin density of −0.80, mainly localized on C_3_, and 2.8 on Mn, consistent with a triplet given by an antiferromagnetic coupling of a quadruplet Mn^IV^‐OH species and a carbon radical (See Table S5 for details). However, as the IRC progresses downhill 31.4 kcal·mol^−1^ from **^3^IRC‐III^e^**, it reaches an intermediate (**^3^III‐*SR***) where the spin density on Mn has decreased to 2.0, and a + 1.1 charge is localized on the cyclohexane scaffold (which has no spin density) (Figure 4a and Table S5). Therefore, **^3^III‐*SR*** corresponds to a couple constituted by an S = 1 Mn^III^‐OH complex and a cationic species. These computational results suggest a highly asynchronous transition state for C─H bond cleavage, where an initial HAT is followed by an ET from the incipient carbon radical to the electrophilic metal center. This mechanistic scenario finds precedent in Mn‐catalyzed and dioxirane promoted C─H bond oxidations in cyclopropane‐containing hydrocarbons that provide structurally rearranged products indicative of the formation of cationic intermediates. ^[^ ^23^, ^24^, ^61^ ^]^ A spin‐crossing leads to the resulting quintuplet cationic intermediate (**^5^III‐*SR***), which is 56.2 kcal·mol^−1^ downhill from **^3^TS‐*SR***, rendering the C_3_─H abstraction process effectively irreversible (Figure 4a). This intermediate species is expected to undergo a rapid OH‐rebound, with the stereochemical information established in the initial enantioselective HAT that is preserved through the overall process (Figure 4b).^[^ ^62^, ^63^ ^]^

The high enantioselectivities achieved with such a low kinetic barrier in the presence of highly reactive electrophilic Mn‐oxo species warrants further discussion. The calculated Gibbs energy difference between **^3^TS‐*SR*** and **^3^TS‐*RS*** [ΔΔG^‡^_calc_ (233.15 K) = 3.8 kcal·mol^−1^] aligns with the experimentally determined value of 99% ee [ΔΔG^‡^_exp_ (233.15 K) = 3.5 kcal·mol^−1^, considering 99.9% ee]. The origin of this substantial Gibbs energy difference between the two competing diastereomeric transition states becomes apparent upon examination of the noncovalent interactions (NCIs) between the incoming substrate **S2** and the reactive (*S,S*)‐[Mn^V^O(ODMB)(^CF3^bpeb)]^2+^ species (Figure S6). Both transition states exhibit an intricate network of NCIs, with the ester carbonyl group maintaining interactions with two methylenic α‐ to N C─H bonds of the catalyst ligand. The main difference lies in the positioning of **S2** in the catalyst pocket. In **^3^TS‐*SR***, one of the C─H bonds of the C_6_ methylene group establishes a C─H···π interaction with the aryl ring of the ^CF3^Bzn^L^ arm (Figure S6).^[^ ^64^, ^65^ ^]^ The NCI isosurface between the edge of **S2** and the π‐face of the aryl moiety of ^CF3^Bzn^L^ further supports the presence of this attractive interaction, which stabilizes **^3^TS‐*SR***. In contrast, such an interaction is absent in **^3^TS‐*RS*** due to the alternative positioning of **S2** within the catalyst pocket. Although the abstraction of both C_3_─H and C_5_─H originates from the same reactant species (**^3^II**), the subtle differences in NCIs in the transition state—combined with the highly structured catalyst active site (as indicated by the V_bur_ (%) analysis)—play a crucial role in achieving the exquisite site‐ and enantioselectivity attained in the non‐directed ketonization of **S2**.

## Substrate Scope

**Figure 5:**
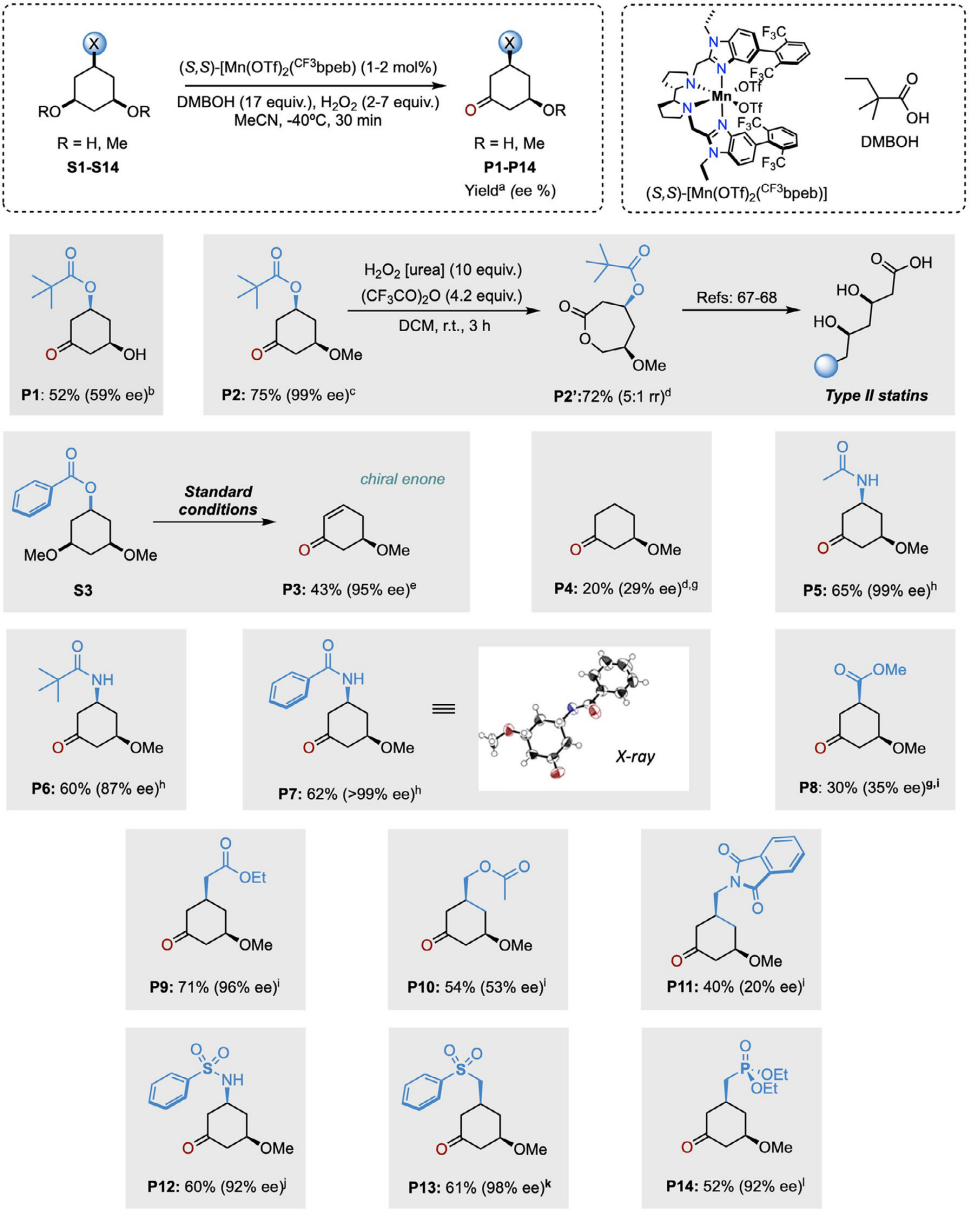
Substrate scope of the enantioselective ketonization. ^a^ Yield of the isolated product. ^b^ Using (*S,S*)‐[Mn(OTf)_2_(^TIPS^mcp)] (1 mol%), H_2_O_2_ (1 equiv.), AcOH. (17 equiv.). ^c^ Using catalyst (2 mol%), H_2_O_2_ (7 equiv.). ^d^ rr: Regioselectivity ratio determined by ^1^H‐NMR. ^e^ Using catalyst (2 mol%) and H_2_O_2_ (3.5 equiv.). ^f^ Using catalyst (1 mol%) and H_2_O_2_ (2 equiv.). ^g^ GC yield. ^h^ Using catalyst (2 mol%) and H_2_O_2_ (5 equiv.). ^i^ Using catalyst (1 mol%) and H_2_O_2_ (3.5 equiv.). ^j^ Using catalyst (2.5 mol%) and H_2_O_2_ (3.5 equiv.). ^k^ Using catalyst (1 mol%) and H_2_O_2_ (2 equiv.). ^l^ Using catalyst (2 mol%) and H_2_O_2_ (5 equiv.).

Having formulated a plausible rationale for the observed site‐ and enantioselectivity enabled by the (*S,S*)‐[Mn(OTf)_2_(^CF3^bpeb)] catalyst in combination with DMBOH in the oxidation of **S2**, we sought to further explore its generality by applying the optimized conditions to the oxidation of C1‐functionalized 3,5‐dimethoxycyclohexane derivatives (**S3**‐**S14**) (Figure 5). These substrates can be readily prepared from resorcinol derivatives through arene hydrogenation under mild conditions (for details see the SI),^[^ ^66^ ^]^ making them attractive platforms for the construction of polyfunctionalized chiral cyclohexanes. Interestingly, **P2** can be converted through Baeyer‐Villiger oxidation to the 7‐membered ring lactone **P2’**, while retaining the stereogenic centers (Figure 5). This compound is a valuable lead for diversification, particularly for products bearing the hallmark chiral 1,3 dioxygenated pattern found in Type II statins.^[^ ^67^, ^68^, ^69^ ^]^

Replacing the pivaloyl by benzoyl group (**S3**) leads to the formation of 5‐methoxycyclohex‐2‐en‐1‐one (**P3**) in 43% isolated yield and excellent enantioselectivity (95% ee). Interestingly, **P3**, stemming from an in situ OBz elimination, is a crucial intermediate in Nicolaou's total synthesis of Rugulin, a naturally occurring bisanthraquinone with potent antibacterial activity. This compound was originally prepared in three steps from the diacetate analogue with an overall yield of 32% and moderate enantioselectivity (65% ee) through an asymmetric ester hydrolysis employing pig liver esterase in stoichiometric amount.^[^ ^70^ ^]^ Gratifyingly, our approach furnishes the same precursor in a single step, in 43% yield and outstanding enantioselectivity using a low catalyst loading (1 mol%).

The oxidative desymmetrization of the simple *cis*‐1,3‐dimethoxycyclohexane (**S4**) delivered 3‐methoxycyclohexanone (**P4**) (20% yield) with low enantioinduction (29% ee). It is worth mentioning that the desymmetrization of **S4** is particularly challenging due to its lower structural complexity, underscoring the crucial role of C1‐substitution in enhancing reaction efficiency and enantio‐discrimination.^[^ ^28^ ^]^ The reaction extends successfully to different *cis*‐3,5‐dimethoxycyclohexyl amides (**S5‐S7**). Thus, the oxidation of acetamide **S5** resulted in the formation of the *N*‐(3‐methoxy‐5‐oxocyclohexyl)acetamide (**P5**) in good isolated yield (65%) and excellent enantioselectivity (99% ee). The introduction of bulkier acyl groups, as in pivalamide **S6** and benzamide **S7**, does not compromise reactivity delivering **P6** and **P7** as the exclusive products with high levels of enantioselectivity (87% and >99% ee, respectively).

The effect of the position of the carbonyl group on the C1 substituent was also investigated. Oxidation of all *cis* methyl 3,5‐dimethoxycyclohexanecarboxylate (**S8**), which features the carbonyl group directly bound to the cyclohexane ring, delivered ketonization product **P8** in moderate isolated yield (30%) and enantioselectivity (35% ee). This outcome can be attributed to the electron‐withdrawing nature of the ester, as recently supported by multivariate linear regression modeling analysis in the ketonization of methylenes.^[^ ^59^ ^]^ However, when a methylene unit is placed between the carbonyl group and the cyclohexane ring, as in ester **S9**, high enantioselectivity is restored (96% ee), and product **P9** was obtained in 71% isolated yield. The high enantioselectivity can be explained as a result of reduced positive charge buildup in the ring. We note in that the chiral motif displayed by **P9** is particularly valuable, en route to the synthesis of a wide variety of terpenoids.^[^ ^71^ ^]^ For example, hydrolysis of the ester group to the free carboxylic acid allows for an increase in molecular complexity by enabling sequential bond formation through directed C─H bond functionalization.^[^ ^72^ ^]^

In contrast, shifting the carbonyl group to the γ position from C1, as in **S10** and **S11**, significantly erodes enantio‐discrimination in products **P10** and **P11** (53% and 20% ee, respectively). As disclosed in our recent work on tertiary C(*sp* ^3^)─H bond hydroxylation,^[^ ^28^ ^]^ the relative position of the carbonyl group is crucial for determining the degree of discrimination between the enantiotopic tertiary C─H bonds. This is likely due to the establishment of H‐bonding interaction between the carbonyl group of the substrate and the α‐ to N C─H bonds of the catalyst.

Encouraged by these results, *cis*‐1,3‐dimethoxycyclohexane frameworks bearing sulfonamide (**S12**), sulfone (**S13**), and phosphonate ester (**S14**) groups were chosen for further exploration. Desymmetrization of **S12** proceeds with high isolated yield of **P12** (60%) and excellent enantioselectivity (92% ee). The access to chiral sulfonamides—bioisosters of amides with enhanced hydrolytic stability—is particularly appealing as they are frequently employed as bacteriostatic antibiotic agents (sulfa drugs).^[^ ^73^ ^]^ Similarly, **S13** and **S14** can be efficiently converted into the chiral products **P13** and **P14**, both exhibiting outstanding levels of enantioselectivity. Interestingly, both α‐ to CH_2_ phosphonate ester and sulfone functionalities can be conveniently employed for olefination reactions through conventional methods,^[^ ^74^, ^75^ ^]^ making chiral **P13** and **P14** products particularly attractive as versatile building blocks for subsequent synthetic elaboration. Collectively, the reaction exhibits a degree of generality and enables the generation of chiral architectures with three different functionalities, allowing expedite orthogonal chemical manipulation. The generation of 3‐methoxycyclohexanone derivatives in a single step can be leveraged to provide access to relevant bioactive compounds such as non‐natural variants of vitamin D3, which display different affinities with the vitamin D binding protein (hDBP).^[^ ^76^ ^]^

## Conclusion

In summary, a highly precise oxidizing system based on a manganese catalyst and aqueous hydrogen peroxide has been evolved to exert control over site‐ and enantioselectivity in the non‐directed C(*sp^3^*)─H bond oxidation of cyclohexyl‐3,5‐*meso* diethers. Through systematic steric manipulation around the reactive high valent Mn‐oxo carboxylato species—achieved by ligand design and the use of a bulky alkanoic acid—a promising strategy for catalyst evolution has emerged. The exceptional enantioselectivities observed when employing this system can be rationalized by the highly structured chiral catalyst pocket, which properly orients the targeted weak tertiary C─H bonds through NCIs in the transition state. Computational studies reveal that the reaction proceeds asynchronously through an initial HAT from the substrate to the Mn‐oxo species followed by ET, leading to the formation of a chiral cationic intermediate. The reaction provides access to chiral 3‐methoxycyclohexanone derivatives via biologically inspired C─H oxidation catalysts, offering a practical route for synthesizing valuable chiral multi‐oxygenated cyclohexane scaffolds in line with sustainability principles. The reaction is also notable because these valuable products have been proven challenging to access in a highly enantioselective manner even by enzymatic methods. We anticipate that continued advancements in catalyst design could pave the way for accessing more efficiently stereochemically defined polyoxygenated structures through asymmetric C─H oxidation.

## Supporting Information

Materials and Methods describing preparation of complexes and substrates, characterization and experimental procedures for the catalytic reactions. Details on the crystallographic data for *(‐)‐* ***P7***.^[^ ^77^ ^]^ NMR Spectra. HPLC traces.

## Author Contributions

A.P. and A.C. contributed equally to this work and were responsible for conducting the experimental and computational studies, respectively. A.G.‐R. and M.S.S. contributed to the data‐science‐driven catalyst optimization, with M.S.S. leading this component of the project. J.M.L. led and supervised the computational transition‐state analysis. M.B., C.N., and M.C. provided conceptual input and strategic guidance throughout the project. M.C. also supervised the overall project and coordinated collaboration between the different research teams. All authors contributed to the preparation of the manuscript.

## Conflict of Interests

The authors declare no conflict of interest.

## Supporting information



Supporting Information

## Data Availability

The data that support the findings of this study are available in the supplementary material of this article.
