# Supplementary material for: Shaping of a Reactive Manganese Catalyst Enables Access to Polyfunctionalized Cyclohexanes via Enantioselective C(sp3 )─H Bond Oxidation of 1,3‐meso Diethers
Source: Angew Chem Int Ed Engl. 2025 Jun 1;64(30):e202507755. doi: 10.1002/anie.202507755 (PMC12281083; doi:10.1002/anie.202507755)
Supplement: Supplementary file 1 — Supporting Information [file ANIE-64-e202507755-s001.docx]

**Shaping of a Reactive Manganese Catalyst Enables Access to Polyfunctionalized Cyclohexanes via Enantioselective C(*sp^3^*)-H Bond Oxidation of 1,3-*meso* Diethers**

Andrea Palone,^a,&^ Arnau Call,^a,&^ Aleria Garcia-Roca,^b^ Josep M. Luis,^a,^* Matthew S. Sigman,^b,^*

Massimo Bietti,^c,^* Cristina Nevado,^d,^* Miquel Costas^a,^*

^a^ Institut de Química Computacional i Catàlisi (IQCC) and Departament de Química, Universitat de

Girona, Campus Montilivi, Girona E-17071, Catalonia, Spain.

^b^ Department of Chemistry, University of Utah, Salt Lake City, Utah, 84112, United States.

^c^ Dipartimento di Scienze e Tecnologie Chimiche, Università “Tor Vergata”, Via della Ricerca

Scientifica, 1 I-00133 Rome, Italy.

^d^ Department of Chemistry, University of Zurich, Winterthurerstrasse 190, CH 8057 Zurich,

Switzerland.

^&^ Equally contributed to this work

e-mail: josepm.luis@udg.edu, sigman@chem.utah.edu, bietti@uniroma2.it,

cristina.nevado@chem.uzh.ch, miquel.costas@udg.edu

- 1. **Materials**

Reagents and solvents were of commercially available reagent quality unless stated otherwise. Solvents were purchased from SDS, Scharlab, and Fluorochem. They were purified and dried either by passing through an activated alumina purification system (M-Braun SPS-800) or using conventional distillation techniques.

- 1. **Instrumentation**

Oxidation products were identified by comparing their GC retention times and GC-MS spectra with those of authentic compounds and/or by ^1^H and ^13^C-NMR analyses. X-ray diffraction analyses were carried out on a BRUKER SMART APEX CCD diffractometer using graphite-monochromated MoKα radiation (λ = 0.71073 Å) from an X-ray tube. NMR spectra were recorded on Bruker DPX400 spectrometers under standard conditions. Electrospray ionization mass spectrometry (ESI-MS) experiments were performed on a Bruker Daltonics Esquire 3000 spectrometer using a 1 mM solution of the analyzed compound. High-resolution mass spectra (HRMS) were recorded on a Bruker MicroTOF-Q II (Q-TOF) instrument with an ESI source at Serveis Tècnics of the University of Girona. Samples were introduced into the mass spectrometer ion source by direct infusion through a syringe pump and externally calibrated using sodium formate. Optical rotations were measured at room temperature (25°C) using a Jasco P-2000 iRM-800 polarimeter. Concentration is expressed in g/100 mL. The cell was 10 cm long with a 1 mL capacity. Chromatographic analyses were performed on an Agilent GC-7820-A chromatograph using an HP-5 column (30 m). Enantiomer resolution was carried out using J&W CYCLOSIL-B columns and supercritical fluid chromatography (SFC) on an Agilent 1260 Infinity II SFC system with a CHIRALPAK IC-3 column

- 1. **Synthesis of the substrates**

### ***Ester synthesis protocol***

The following ester substrates were synthesized starting from boronic ester **A**:^[1]^

A round-bottom flask equipped with a septum and maintained under a nitrogen atmosphere was charged with a 0.40 M solution of 3-phenyl-2,4-dioxa-3-borabicyclo[3.3.1]nonan-7-ol (**A**) (1 equiv.) in dry dichloromethane, along with triethylamine (TEA) (2 equiv.) and 4-dimethylaminopyridine (DMAP) (10 mol%). The reaction mixture was cooled to 0 °C, and the acyl chloride or anhydride (2 equiv.) was added dropwise over 10 minutes. The reaction was then stirred for 18 hours, allowing the temperature to gradually rise to room temperature. After completion, the reaction mixture was quenched by adding 1M HCl until pH=1, then diluted with dichloromethane. The organic layer was separated, and the aqueous layer was extracted with dichloromethane (2×). The combined organic extracts were washed with saturated NaHCO₃, dried over anhydrous sodium sulfate (Na₂SO₄), and concentrated under reduced pressure. The resulting intermediate B was used directly in the next step without further purification

A round-bottom flask equipped with a septum and maintained under a nitrogen atmosphere was charged with a 0.50 M solution of **B** (1 equiv.) in dry dichloromethane and pinacol (2 equiv.). BF₃·Et₂O (0.5 equiv.) was then added dropwise over 10 minutes, and the reaction mixture was stirred for 4 hours. After solvent removal, the crude product was purified by flash chromatography on silica gel using ethyl acetate as the eluent

 **S1**: The product was isolated as a white solid (2.10 g, 53% yield). ^1^H NMR (400 MHz, CDCl_3_) δ, ppm: 4.78 (tt, *J* = 10.1, 4.2 Hz, 1H), 3.81 – 3.78 (m, 2H), 2.27 – 2.11 (m, 3H), 1.79 (s, 2H), 1.47 (dtd, *J* = 12.1, 10.0, 7.4 Hz, 3H), 1.19 (s, 9H). ^13^C NMR (101 MHz, CDCl_3_) δ, ppm: 178.0, 67.6, 65.8, 43.0, 39.5, 38.8, 27.2. HRMS(ESI+) *m/z* calculated for C_11_H_20_O_4_ [M+Na]^+^ 239.1254, found 239.1256.

A round-bottom flask equipped with a septum and maintained under a nitrogen atmosphere was charged with a 0.35 M solution of diol **S1** (1.70 g, 8.0 mmol, 1 equiv.) in dry THF, along with CH₃I (14 mL, 0.22 mol, 28 equiv.). The reaction mixture was cooled to 0 °C, and sodium hydride (580 mg, 24 mmol, 3 equiv.) was added portion-wise as a solid over 10 minutes under nitrogen flow. The reaction was stirred for 18 hours at room temperature. After completion, the reaction mixture was cooled to 0 °C and carefully quenched under nitrogen flow by adding a saturated aqueous NH₄Cl solution. The mixture was then diluted with Et₂O, and the organic layer was separated. The aqueous layer was extracted with Et₂O (2×), and the combined organic extracts were washed with brine and dried over anhydrous magnesium sulfate (MgSO₄). The solvent was removed under reduced pressure, and the crude product was purified by flash chromatography on silica gel using hexane/ethyl acetate (5:1) to afford **S2** as a yellow oil (1.7 g, 91% yield). ^1^H NMR (400 MHz, CDCl_3_) δ, ppm: 4.65 (tt, *J* = 11.7, 4.4 Hz, 1H), 3.35 (s, 6H), 3.20 (tt, *J* = 11.4, 4.1 Hz, 2H), 2.43 (dtt, *J* = 11.7, 3.9, 1.8 Hz, 1H), 2.34 (dtd, *J* = 10.8, 3.9, 1.8 Hz, 2H), 1.35 – 1.09 (m, 12H). ^13^C NMR (101 MHz, CDCl_3_) δ, ppm: 178.1, 74.4, 67.8, 56.4, 38.8, 37.6, 37.0, 27.2. HRMS(ESI+) *m/z* calculated for C_13_H_24_O_4_ [M+Na]^+^ 267.1567, found 267.1573.

The synthesis of **S3** followed the same procedure as **S2**. The product was isolated as a yellow oil (1.10 g, 41% yield over three steps). ^1^H NMR (400 MHz, CDCl_3_) δ, ppm: 8.07 – 8.01 (m, 2H), 7.61 – 7.54 (m, 1H), 7.44 (dd, *J* = 8.4, 7.1 Hz, 2H), 4.94 (tt, *J* = 11.7, 4.3 Hz, 1H), 3.38 (s, 6H), 3.28 (tt, *J* = 11.3, 4.1 Hz, 2H), 2.50 (ddtd, *J* = 11.1, 9.3, 3.8, 3.1, 1.7 Hz, 3H), 1.42 (q, *J* = 11.6 Hz, 2H), 1.22 (q, *J* = 11.6 Hz, 1H). ^13^C NMR (101 MHz, CDCl_3_) δ, ppm: 166.0, 133.2, 130.4, 129.8, 128.5, 74.4, 68.8, 56.4, 37.6, 37.2. HRMS(ESI+) *m/z* calculated for C_15_H_20_O_4_ [M+Na]^+^ 287.1254, found 287.1258.

***Synthesis of substrate S4***

A round-bottom flask equipped with a septum and kept under nitrogen was charged with a 0.35 M solution of *cis*-cyclohexane-1,3-diol (0.50 g, 4.3 mmol, 1 equiv.) in dry THF, followed by the addition of CH₃I (7 mL, 0.11 mol, 25 equiv.). The reaction mixture was then cooled to 0 °C. Sodium hydride (310 mg, 13 mmol, 3 equiv.) was added as a solid, portion-wise, over 10 minutes under a nitrogen flow, and the reaction was left stirring for 18 hours. At this point, the reaction mixture was cooled to 0 °C and carefully quenched under nitrogen flow by adding a saturated aqueous NH₄Cl solution. The reaction mixture was diluted with Et₂O, and the organic layer was separated from the aqueous layer. The aqueous layer was extracted with Et₂O (2×), and the organic layers were combined. The organic layer was washed with brine and dried over anhydrous MgSO₄. After the solvent was removed, **S4** was purified by flash chromatography over silica using a hexane:ethyl acetate 10:1 mixture, yielding S4 (300 mg, 63%) as a yellow oil. ^1^H NMR (400 MHz, CDCl_3_) δ, ppm: 3.38 (s, 6H), 3.14 (tt, *J* = 10.4, 4.0 Hz, 2H), 2.47 (dtt, *J* = 11.8, 4.0, 2.1 Hz, 1H), 2.11 – 2.00 (m, 2H), 1.88 – 1.81 (m, 1H), 1.22 – 1.05 (m, 4H).

###

### ***Amides synthesis protocol***

Substrates **S5**, **S6** and **S7** were obtained starting from the free amine **C**.

In a dry vial equipped with a stir bar, 788 mg of 3,5-dimethoxyaniline (5.14 mmol, 1 equiv.) was dissolved in 4 mL of methanol and 2 mL of acetic acid (0.9 M). Then, Rh(Al₂O₃) (80 mg, 10% w/w) was added, and the vial was placed in a Parr high-pressure hydrogenation apparatus. The system was purged with nitrogen for 10 minutes with vigorous stirring. The vessel was slowly pressurized to 5 bar with hydrogen gas, then allowed to vent; this process was repeated three times. After pressurization to 10 bar, the mixture was stirred for 48 hours at room temperature. After this time, the vessel was slowly depressurized and opened. The mixture was filtered over a Celite, and the resulting solution was evaporated under reduced pressure. The resulting solid was suspended in toluene and subjected to rotary evaporation. The crude amine **C** was immediately used in the next step without further purification.

To a freshly prepared solution **C** in CH₂Cl₂ (18 mL of 0.1 M solution, 1.8 mmol) was added triethylamine (1.0 equiv), then the reaction mixture was cooled to 0 °C. The acyl chloride (1.0 equiv) was added dropwise over 10 minutes and the reaction was left stirring overnight, allowing it to warm up to room temperature. At this point, a saturated aqueous Na₂CO₃ solution was added until pH~10-11 and then diluted with dichloromethane. The organic layer was separated from the basic aqueous layer. The aqueous layer was extracted with dichloromethane (2×) and the organic layers were combined. The organic layer was washed with 1M HCl and dried over anhydrous Na₂SO₄. The organic layer was evaporated to dryness, and the crude amide was purified by flash chromatography over silica gel.

**S5:** Following the general conditions of a*mides synthesis protocol*, the crude mixture was purified by flash chromatography over silica using a hexane:ethyl acetate 1:1 mixture, and the product was concentrated to dryness. The product was isolated as a white solid (75% yield, over 2 steps).

^1^H NMR (400 MHz, CDCl_3_) δ, ppm: 5.50 (d, *J* = 8.3 Hz, 1H), 3.87 (tdt, *J* = 11.9, 8.2, 4.0 Hz, 1H), 3.35 (s, 6H), 3.25 (tt, *J* = 10.5, 4.0 Hz, 2H), 2.42 – 2.34 (m, 1H), 2.30 – 2.22 (m, 2H), 1.97 (s, 3H), 1.15 (dtd, *J* = 34.1, 11.9, 10.5 Hz, 3H). ^13^C NMR (101 MHz, CDCl_3_) δ, ppm: 169.3, 75.2, 56.4, 43.8, 37.8, 37.2, 23.6. HRMS(ESI+) *m/z* calculated C_10_H_19_NO_3_ [M+Na]^+^ 224.1257, found 224.1253.

**S6:** Following the general conditions of a*mides synthesis protocol*, the crude mixture was purified by flash chromatography over silica using a hexane:ethyl acetate 1:1 mixture, and the product was concentrated to dryness. The product was isolated as a white solid (43% yield, over 2 steps).

^1^H NMR (400 MHz, CDCl_3_) δ, ppm: 5.87 (d, *J* = 8.2 Hz, 1H), 3.90 (tdt, *J* = 10.4, 8.2, 4.0 Hz, 1H), 3.39 – 3.24 (m, 8H), 2.28 (dtd, *J* = 13.8, 3.6, 1.4 Hz, 1H), 2.20 – 2.11 (m, 2H), 1.34 (dt, *J* = 12.2, 9.7 Hz, 1H), 1.28 – 1.15 (m, 11H). ^13^C NMR (101 MHz, CDCl_3_) δ, ppm: 177.5, 75.1, 56.3, 43.2, 38.5, 36.8, 36.8, 27.5. HRMS(ESI+) *m/z* calculated for C_13_H_25_NO_3_ [M+Na]^+^ 266.1727, found 266.1729.

**S7:** Following the general conditions of a*mides synthesis protocol*, the crude mixture was purified by flash chromatography over silica using a hexane:ethyl acetate 1:1 mixture and the product was concentrated to dryness. The product was isolated as a white solid (63% yield, over 2 steps).

^1^H NMR (400 MHz, CDCl_3_) δ, ppm: 7.83 – 7.69 (m, 2H), 7.52 – 7.47 (m, 1H), 7.43 (ddt, *J* = 8.3, 6.6, 1.4 Hz, 2H), 6.47 (d, *J* = 8.4 Hz, 1H), 4.15 (dddd, *J* = 14.3, 10.4, 8.3, 4.0 Hz, 1H), 3.36 (s, 8H), 2.31 (ddt, *J* = 16.1, 12.4, 3.7 Hz, 3H), 1.47 – 1.33 (m, 3H). ^13^C NMR (101 MHz, CDCl_3_) δ, ppm: 166.6, 134.7, 131.6, 128.7, 127.0, 75.3, 56.4, 44.1, 36.9, 36.9. HRMS(ESI+) *m/z* calculated for C_15_H_21_NO_3_ [M+Na]^+^ 286.1414, found 286.1416.

***Synthesis of substrate S8***

**S8** was prepared according to a slight modification of a reported procedure.^[2]^

In a dry vial equipped with a stir bar, 1 g of methyl 3,5-dimethoxybenzoate (5.09 mmol, 1 equiv.) was dissolved in 8 mL of methanol and 22 µL of acetic acid (0.6 M). Then, Rh(Al₂O₃) (100 mg, 10% w/w) was added, and the vial was placed in a Parr high-pressure hydrogenation apparatus. The system was purged with nitrogen for 10 minutes with vigorous stirring. The vessel was slowly pressurized to 5 bar with hydrogen gas, then allowed to vent; this process was repeated three times. After pressurization to 10 bar, the mixture was stirred for 48 hours at room temperature. After this time, the vessel was slowly depressurized and opened. The mixture was filtered over Celite, and the resulting solution was evaporated under reduced pressure. Then, **S8** was purified by flash chromatography over silica using a pentane:diethyl ether 2:1 mixture to obtain **S8** (800 mg, 78% yield) as a colorless oil. ^1^H NMR (400 MHz, CDCl_3_) δ, ppm: 3.69 (s, 3H), 3.36 (s, 6H), 3.17 (tt, *J* = 11.3, 4.0 Hz, 2H), 2.44 (dtt, *J* = 11.8, 4.0, 1.9 Hz, 1H), 2.35 – 2.21 (m, 3H), 1.35 – 1.23 (m, 2H), 1.14 (q, *J* = 11.5 Hz, 1H). ^13^C NMR (101 MHz, CDCl_3_) δ, ppm: 174.8, 76.7, 56.2, 52.1, 38.5, 37.8, 33.8. HRMS(ESI+) *m/z* calculated for C_10_H_18_O_4_ [M+Na]^+^ 225.1097, found 225.1104.

***Synthesis of substrate S9***

A round-bottom flask equipped with a septum and kept under nitrogen was cooled to 0°C, then charged with lithium aluminium hydride (115 mg, 3 mmol, 1.1 equiv.) and 10 mL of dry THF. At this point, **S8** in THF solution (553 mg, 2.7 mmol, 1 equiv.) was added slowly under nitrogen flow. The reaction was left stirring overnight, allowing it to warm up to room temperature. After this, the reaction mixture was quenched with 1 M NaOH solution at 5°C. After filtration through Celite, and extraction with ethyl acetate (2 × 50 mL), the organic fractions were dried over anhydrous Na₂SO₄, filtered, and evaporated to dryness to obtain the primary alcohol intermediate **A1** (500 mg, 96% yield) as a yellow oil without further purification. ^1^H NMR (400 MHz, CDCl_3_) δ, ppm: 3.55 (t, *J* = 5.7 Hz, 2H), 3.36 (s, 6H), 3.19 (tt, *J* = 11.3, 4.1 Hz, 2H), 2.47 (dtt, *J* = 11.7, 4.0, 2.0 Hz, 1H), 2.17 – 2.08 (m, 2H), 1.58 – 1.44 (m, 2H), 1.11 (q, *J* = 11.4 Hz, 1H), 0.87 (td, *J* = 12.3, 11.0 Hz, 2H). ^13^C NMR (101 MHz, CDCl_3_) δ, ppm: 77.1, 67.6, 55.9, 38.1, 35.6, 34.4. ESI-MS *m/z* found for C_9_H_18_O_3_ [M+Na]^+^ 197.1.

A round-bottom flask equipped with a septum and kept under nitrogen was charged with alcohol **A1** (203 mg, 1.2 mmol, 1 equiv), triethylamine (230 µL, 1.68 mmol, 1.4 equiv), and 15 mL of dry dichloromethane. The reaction mixture was then cooled to 0°C. At this point, methanesulfonyl chloride (112 µL, 1.45 mmol, 1.2 equiv) was delivered over a period of 40 minutes by syringe pump into the solution, and the reaction was left stirring at the same temperature for an additional 20 minutes. The reaction mixture was successively washed with the following solutions: H₂O, 1 M HCl, NaHCO₃ (aq, sat), and NaCl (aq, sat). The organic layer was dried over anhydrous Na₂SO₄, filtered, and evaporated to dryness to obtain **A2** (241 mg, 82% yield) as a yellow oil without further purification. ^1^H NMR (400 MHz, CDCl_3_) δ, ppm: 4.11 (d, *J* = 6.4 Hz, 2H), 3.37 (s, 6H), 3.19 (tt, *J* = 11.2, 4.1 Hz, 2H), 3.02 (s, 3H), 2.53 – 2.46 (m, 1H), 2.14 (d, *J* = 12.3 Hz, 2H), 1.79 (dddq, *J* = 12.7, 9.6, 6.4, 3.2 Hz, 1H), 1.12 (q, *J* = 11.4 Hz, 1H), 0.94 (td, *J* = 12.4, 11.1 Hz, 2H). ^13^C NMR (101 MHz, CDCl_3_) δ, ppm: 76.5, 73.3, 56.1, 37.8, 37.4, 34.0, 32.9. ESI-MS *m/z* found for C_10_H_20_O_5_S [M+Na]^+^ 275.1.

A round-bottom flask was charged with **A2** (651 mg, 2.58 mmol, 1 equiv), NaI (96 mg, 0.64 mmol, 0.25 equiv), NaCN (506 mg, 10.3 mmol, 4 equiv), and 8 mL of N,N-dimethylformamide. The reaction mixture was then heated slowly to 70°C and left stirring overnight. After this, the reaction mixture was cooled to room temperature and diluted with ethyl acetate. The reaction mixture was washed with H₂O (3×) and NaCl (aq, sat). The organic layer was dried over anhydrous Na₂SO₄, filtered, and evaporated to dryness to obtain **A3** (241 mg, 51% yield) as a yellow oil without further purification. ^1^H NMR (400 MHz, CDCl_3_) δ, ppm: 3.37 (s, 6H), 3.19 (tt, *J* = 11.2, 4.1 Hz, 2H), 2.48 (ddt, *J* = 10.1, 4.1, 2.1 Hz, 1H), 2.36 (d, *J* = 6.8 Hz, 2H), 2.19 (d, *J* = 12.2 Hz, 2H), 1.71 (tdq, *J* = 13.6, 6.7, 3.3 Hz, 1H), 1.11 (q, *J* = 11.5 Hz, 1H), 1.00 (td, *J* = 12.3, 10.9 Hz, 2H). ^13^C NMR (101 MHz, CDCl_3_) δ, ppm: 118.1, 76.3, 56.1, 37.4, 37.2, 30.1, 24.2. ESI-MS *m/z* found for C_10_H_17_NO_2_ [M+Na]^+^ 206.2.

A round-bottom flask was charged with **A3** (241 mg, 1.3 mmol, 1 equiv), NaOH (520 mg, 13.1 mmol, 10 equiv), and 3 mL of a mixture of EtOH:water (2:1). The reaction mixture was then heated slowly to 80°C and left stirring overnight. After this, the reaction mixture was cooled to room temperature, almost all of the solvent was evaporated, and it was diluted with ethyl acetate. The reaction mixture was washed with 1 M HCl (3×) and NaCl (aq, sat). The organic layer was dried over anhydrous Na₂SO₄, filtered, and then evaporated to dryness to obtain the crude intermediate, which was used in the next step without further purification.

To the crude mixture, 3 mL of ethanol and one drop of sulfuric acid were added. The reaction mixture was then heated to reflux and left stirring overnight. After this, the reaction mixture was cooled to room temperature, almost all of the solvent was evaporated, and it was diluted with ethyl acetate. The reaction mixture was washed with H₂O (3×) and NaCl (aq, sat). The organic layer was dried over anhydrous Na₂SO₄, filtered, and evaporated to dryness. The crude product was then purified by flash chromatography over silica using hexane:ethyl acetate (50:1) to obtain **S9** (115 mg, 50% yield over two steps) as a yellow oil. ^1^H NMR (400 MHz, CDCl_3_) δ, ppm: 4.14 (q, *J* = 7.1 Hz, 2H), 3.35 (s, 6H), 3.18 (tt, *J* = 11.2, 4.1 Hz, 2H), 2.46 (dtt, *J* = 11.6, 3.9, 1.8 Hz, 1H), 2.28 (d, *J* = 7.0 Hz, 2H), 2.12 – 2.03 (m, 2H), 1.81 (ddtd, *J* = 15.7, 12.3, 7.0, 3.4 Hz, 1H), 1.26 (t, *J* = 7.2 Hz, 3H), 1.06 (q, *J* = 11.4 Hz, 1H), 0.89 (td, *J* = 12.2, 11.0 Hz, 2H). ^13^C NMR (101 MHz, CDCl_3_) δ, ppm: 172.4, 76.8, 60.4, 56.0, 55.8, 41.1, 37.7, 37.5, 29.7, 14.3. HRMS(ESI+) *m/z* calculated for C_12_H_22_O_4_ [M+Na]^+^ 253.1410, found 253.1415.

***Synthesis of intermediate B1***

A round-bottom flask equipped with a septum and kept under nitrogen was charged with alcohol **A1** (456 mg, 2.62 mmol, 1 equiv), carbon tetrabromide (1.74 g, 5.24 mmol, 2.0 equiv), and 15 mL of dry dichloromethane. The reaction mixture was then cooled to 0°C. At this point, triphenylphosphine (1.37 g, 5.24 mmol, 2.0 equiv) was added as a solid under nitrogen flow into the solution, and the reaction was left stirring for 2 hours, allowing it to warm up to room temperature. The reaction mixture was then evaporated to dryness, and the crude product was purified by flash chromatography over silica using hexane:ethyl acetate (5:1) to obtain **B1** (435 mg, 70% yield) as a colorless oil. ^1^H NMR (400 MHz, CDCl_3_) δ, ppm: 3.39 (s, 8H), 3.21 (tt, *J* = 11.2, 4.1 Hz, 2H), 2.47 (dtt, *J* = 11.9, 4.0, 1.9 Hz, 1H), 2.30 – 2.20 (m, 2H), 1.70 (dddq, *J* = 15.4, 9.3, 6.2, 3.2 Hz, 1H), 1.12 (q, *J* = 11.4 Hz, 1H), 0.97 (td, *J* = 12.3, 11.1 Hz, 2H). ^13^C NMR (101 MHz, CDCl_3_) δ, ppm: 76.7, 56.0, 38.8, 37.7, 36.5, 35.3. HRMS (ESI+) *m/z* calculated for C_19_H_17_BrO_2_ [M+H]^+^ 237.0485, found 237.0486.

***Synthesis of substrate S10***

A round-bottom flask equipped with a septum and kept under nitrogen was charged with alcohol **A1** (80 mg, 0.46 mmol, 1 equiv), triethylamine (128 µL, 0.92 mmol, 2.0 equiv), and 15 mL of dry dichloromethane. The reaction mixture was then cooled to 0°C. At this point, acetic anhydride (87 µL, 0.92 mmol, 2.0 equiv) was added to the solution, and the reaction was left stirring for 18 hours, allowing it to warm up to room temperature. The reaction mixture was successively washed with the following solutions: H₂O, 1 M HCl, NaHCO₃ (aq, sat), and NaCl (aq, sat). The organic layer was dried over anhydrous Na₂SO₄, filtered, and evaporated to dryness to obtain **S10** (89 mg, 90% yield) as a yellow oil without further purification. ^1^H NMR (400 MHz, CDCl_3_) δ, ppm: 4.00 (d, *J* = 6.3 Hz, 2H), 3.39 (s, 6H), 3.20 (tt, *J* = 11.2, 4.1 Hz, 2H), 2.50 (dtt, *J* = 11.7, 4.0, 2.0 Hz, 1H), 2.14- 2.06 (m, 5H), 1.70 (ttt, *J* = 12.7, 6.5, 3.4 Hz, 1H), 1.12 (q, *J* = 11.4 Hz, 1H), 0.94 (td, *J* = 12.3, 11.0 Hz, 2H). ^13^C NMR (101 MHz, CDCl_3_) δ, ppm: 171.1, 76.8, 68.4, 56.0, 37.8, 34.5, 32.4, 20.9. HRMS (ESI+) *m/z* calculated for C_11_H_20_O_4_ [M+Na]^+^ 239.1254, found 239.1249.

***Synthesis of substrate S11***

A round-bottom flask was charged with **A3** (227 mg, 0.90 mmol, 1 equiv), KI (14 mg, 0.09 mmol, 0.1 equiv), potassium phthalimide (250 mg, 1.35 mmol, 1.5 equiv), and 6 mL of N,N-dimethylformamide. The reaction mixture was then heated slowly to 80°C and left stirring overnight. After this, the reaction mixture was cooled to 0°C, quenched with 10 mL of water, and washed with EtOAc (3×). The combined organic layers were washed with H₂O and NaCl (aq, sat) and dried over anhydrous Na₂SO₄, filtered, and evaporated to dryness. The crude amide was then purified by flash chromatography over silica using hexane:ethyl acetate (10:1) to obtain **S11** (169 mg, 62% yield) as a white solid. ^1^H NMR (400 MHz, CDCl_3_) δ, ppm: 7.87 (dd, *J* = 5.5, 3.1 Hz, 2H), 7.75 (dd, *J* = 5.5, 3.0 Hz, 2H), 3.66 (d, *J* = 7.2 Hz, 2H), 3.35 (s, 6H), 3.16 (tt, *J* = 11.2, 4.1 Hz, 2H), 2.53 – 2.44 (m, 1H), 2.05 (d, *J* = 12.2 Hz, 2H), 1.87 (ddt, *J* = 12.5, 8.3, 4.0 Hz, 1H), 1.17 – 1.07 (m, 1H), 0.99 (q, *J* = 12.1 Hz, 2H). ^13^C NMR (101 MHz, CDCl_3_) δ, ppm: 168.5, 134.0, 132.0, 123.3, 76.7, 56.0, 43.2, 37.6, 35.6, 32.3. HRMS (ESI+) *m/z* calculated for C_17_H_21_NO_4_  [M+Na]^+^ 326.1368, found 326.1370.

***Synthesis of substrate S12***

 **S12**: Following the general conditions of a*mides synthesis protocol*, the crude mixture was purified by flash chromatography over silica using hexane:ethyl acetate (1:1), and the product was concentrated to dryness. The product was isolated as a white solid (25% yield, over 2 steps). ^1^H NMR (400 MHz, CDCl_3_) δ, ppm: 7.96 – 7.85 (m, 2H), 7.65 – 7.49 (m, 3H), 4.92 (d, *J* = 8.5 Hz, 1H), 3.29 (s, 7H), 3.14 (tt, *J* = 10.2, 4.0 Hz, 2H), 2.28 (dtd, *J* = 12.0, 3.9, 1.9 Hz, 1H), 2.13 – 2.01 (m, 2H), 1.29 – 1.12 (m, 3H). ^13^C NMR (101 MHz, CDCl_3_) δ, ppm: 141.4, 132.7, 129.2, 126.8, 74.7, 56.1, 48.3, 38.2, 36.4. HRMS (ESI+) *m/z* calculated for C_14_H_21_NO_4_S [M+Na]^+^ 322.1084, found 322.1084.

***Synthesis of substrate S13***

In a crimped vial equipped with a magnetic stir bar, thiophenol (122 µL, 1.2 mmol, 1.2 equiv), K₂CO₃ (198 mg, 1.5 mmol, 1.5 equiv), **B1** (237 mg, 1 mmol, 1.0 equiv), and DMSO (4 mL) were added. After 10 minutes at room temperature, the reaction mixture was heated to 50°C and stirred for 2 hours. After complete substrate conversion, Et₂O (5 mL) was added, and the organic phase was extracted with water (3×10 mL). The organic phase was then dried over anhydrous MgSO₄. After filtration, the solution was concentrated under vacuum, and the crude compound was used for the next step without further purification.

In a round-bottom flask, the sulfide intermediate was dissolved in 5 mL of a 1:1 solution of methanol and water. Oxone (922 mg, 3 mmol, 3 equiv, with respect to **B1**) was added, and the reaction was stirred at room temperature for 4 hours. After complete substrate conversion, the crude reaction mixture was concentrated under vacuum to remove the methanol, and then extracted with CH₂Cl₂ (3×30 mL). The combined organic layers were dried over anhydrous MgSO₄, filtered, and concentrated under vacuum. The crude compound was then purified by flash chromatography on silica gel using hexane:ethyl acetate (1:1), and the product was concentrated to dryness. The product **S13** was isolated as a colorless oil (100 mg, 35% yield, over 2 steps). ^1^H NMR (400 MHz, CDCl_3_) δ, ppm: 7.95 – 7.90 (m, 2H), 7.70 – 7.63 (m, 1H), 7.62 – 7.55 (m, 2H), 3.33 (s, 6H), 3.16 (tt, *J* = 11.2, 4.1 Hz, 2H), 3.06 (d, *J* = 6.2 Hz, 2H), 2.48 – 2.41 (m, 1H), 2.27 (dd, *J* = 10.0, 6.1 Hz, 2H), 2.09 (dddp, *J* = 12.5, 9.5, 6.3, 3.3 Hz, 1H), 1.06 (d, *J* = 11.5 Hz, 1H), 1.00 – 0.91 (m, 2H). ^13^C NMR (101 MHz, CDCl_3_) δ, ppm: 140.2, 133.8, 129.4, 127.8, 76.3, 62.0, 56.1, 37.8, 37.6, 27.8. HRMS (ESI+) *m/z* calculated for C_15_H_22_O_4_S [M+Na]^+^ 321.1131, found 321.1134.

***Synthesis of substrate S14***

In a crimped vial equipped with a magnetic stir bar, **B1** (93 mg, 0.4 mmol, 1.0 equiv) was added along with 2 mL of triethyl phosphite. The reaction mixture was then heated to 120°C and stirred for 2 days. Subsequently, the temperature was raised to 140°C and maintained under stirring for an additional day. The crude reaction mixture was cooled to room temperature and then directly charged into the column. It was purified by flash chromatography on silica gel using DCM:EtOH (9:1), and the product was concentrated to dryness. The product was isolated as a colorless oil (24 mg, 20% yield). ^1^H NMR (400 MHz, CDCl_3_) δ, ppm: 4.15 – 4.04 (m, 4H), 3.36 (s, 6H), 3.16 (tt, *J* = 11.2, 4.1 Hz, 2H), 2.45 (d, *J* = 11.5 Hz, 1H), 2.25 (d, *J* = 12.2 Hz, 2H), 1.78 – 1.73 (m, 2H), 1.33 (t, *J* = 7.1 Hz, 6H), 1.08 (t, *J* = 11.4 Hz, 1H), 0.97 – 0.88 (m, 2H). ^13^C NMR (101 MHz, CDCl_3_) δ, ppm: 77.2, 61.5 (d, *J* = 6.8 Hz), 56.0, 39.2 (d, *J* = 10.2 Hz), 37.5, 32.5 (d, *J* = 139.5 Hz), 27.8 (d, *J* = 4.4 Hz), 16.5 (d, *J* = 6.1 Hz). ^31^P NMR (162 MHz, CDCl_3_) δ, ppm: 30.54. GC-MS (CI) [M – O + H]^+^ m/z = 279.0

- 1. **Synthesis of the complexes**

Triflate complexes with (*S,S*) and (*R,R*) configuration, Mn(pdp), Mn(^NMe2^pdp), Mn(^DMM^pdp), Mn(^TIPS^pdp), Fe(^TIPS^pdp), Mn(^TIPS^mcp), Mn(^TIPS^ecp), Mn(^H^bpeb), Mn(^CF3^bpeb) and Mn(^iPr^bpeb) were synthesized according to reported procedures.^[3-8]^

**Figure S1.** Schematic representation of the catalysts used in this work.

**1.5. Oxidation reactions**

**1.5.1. Reaction protocol for catalysis**

The substrate (50 µmol, 1 equiv.), carboxylic acid (850 µmol, 17 equiv.), and the corresponding catalyst (0.5 µmol, 1 mol%) were dissolved in 0.4 mL of acetonitrile (CH₃CN) to achieve a substrate concentration of 0.125 M, in a 10 mL vial equipped with a stir bar, cooled to -40°C in a CH₃CN/N₂(liq) bath. Then, 56 µL of a 0.9 M hydrogen peroxide solution in CH₃CN (1.0 equiv.) diluted from commercially available H₂O₂ (50% H₂O₂ solution in water, Aldrich) was delivered over a period of 30 minutes by syringe pump into the solution. The reaction mixture was left under stirring for an additional 5 minutes. At this point, the reaction was quenched with 100 µmol of 2-propanol, and an internal standard (biphenyl) was added. The solution was quickly filtered through a short plug of silica, which was subsequently rinsed with 2 x 1 mL AcOEt. GC analysis of the solution provided substrate conversions and product yields relative to the internal standard integration. Commercially unavailable products were identified by a combination of ¹H, ¹³C-NMR analysis, and HRMS. Enantiomeric excesses were determined by GC equipped with a chiral column, and the oxidized products were identified by comparison to the GC retention time of racemate products.

**1.5.2. General Procedure for product isolation**

A 10 mL round-bottom flask was charged with the substrate (500 μmol, 1 equiv.), carboxylic acid (8.5 mmol, 17 equiv.), catalyst (5 μmol, 1.0 mol%), and CH₃CN (4 mL). The mixture was then cooled to -40 ºC in a CH₃CN/N₂(liq) bath under magnetic stirring. At this point, 556 μL of a 0.9 M hydrogen peroxide solution in CH₃CN (1.0 equiv.) were added by syringe pump over a period of 30 minutes at -40°C. The reaction mixture was left under stirring for an additional 5 minutes. Some oxidation products (**P2**, **P3**, **P8**) are unstable, as also mentioned by Nicolaou and co-workers in a published work on analogous compounds.^[9]^ Therefore, two different workup procedures have been developed for their isolation.

Workup A: A cold 10 mL aqueous NaHCO₃ saturated solution (stored in the fridge at 3–5 ºC) was added to the reaction mixture still in the -40 ºC bath. The resultant solution was rapidly extracted with CH₂Cl₂ (3 x 10 mL). The organic fractions were combined, dried over MgSO₄, and the solvent was evaporated to dryness. The crude oxidized product was then purified by flash chromatography over silica gel.

Workup B: 500 µmol of 2-propanol was added to the reaction mixture while still in the -40 ºC bath. The crude oxidized mixture was then directly loaded onto a flash chromatography column over silica gel and purified with the eluent of choice.

*Note: The oxidation products showed partial instability upon purification over silica gel, resulting in minor impurities and solvent peaks in the NMR spectra. Despite multiple purification attempts, we were not able to obtain the products in a purer form. To mitigate decomposition, purification was performed with minimal exposure to silica, which improved recovery and spectral quality.*

**1.5.3. Optimization reaction conditions**

**Reaction optimization for oxidation of S1**

**Table S1.** Catalyst screening.

| Entry | Catalyst | Deviation | Conv. (%)^a^ | Yield P1 (%)^a^ | ee P1 (%)^b^ |
| --- | --- | --- | --- | --- | --- |
| 1 | [Mn(OTf)_2_(pdp)] | - | 70 | 51 | 32 |
| 2 | [Mn(OTf)_2_(^DMM^pdp)] | - | 62 | 50 | 30 |
| 3 | [Mn(OTf)_2_(^TIPS^pdp)] | - | 60 | 50 | 51 |
| 4^c^ | [Mn(OTf)_2_(^TIPS^pdp)] | HFIP instead of MeCN | 71 | 43 | 46 |
| 5^c^ | [Mn(OTf)_2_(^TIPS^pdp)] | TFE instead of MeCN | 62 | 47 | 24 |
| 6 | [Fe(OTf)_2_(^TIPS^pdp)] | - | 45 | 39 | 49 |
| 7 | [Mn(OTf)_2_(^TIPS^mcp)] | - | 69 | 57 (52)^d^ | 59 |
| 8^c^ | [Mn(OTf)_2_(^TIPS^mcp)] | - | 71 | 53 | 59 |
| 9 | [Mn(OTf)_2_(^TIPS^ecp)] | - | 40 | 28 | 60 |
| 10 | [Mn(OTf)_2_(^CF3^bpeb)] | - | 50 | 40 | 29 |

^a^ Conversions and yields determined by GC analysis of two or three different independent runs. ^b^ ee values determined by GC. ^c^ Reaction performed at 0 ºC. ^d^ Yield of the isolated product.

**Table S2.** Co-ligand screening.

| Entry | Catalyst | Carboxylic acid | Conv. (%)^a^ | Yield P1 (%)^a^ | ee (%)^b^ |
| --- | --- | --- | --- | --- | --- |
| 1 | [Mn(OTf)_2_(^TIPS^pdp)] | AcOH | 60 | 50 | 51 |
| 2 | [Mn(OTf)_2_(^TIPS^pdp)] | CpOH | 66 | 50 | 42 |
| 3 | [Mn(OTf)_2_(^TIPS^pdp)] | ClAcOH ^c^ | 73 | 56 | 40 |
| 4 | [Mn(OTf)_2_(^TIPS^mcp)] | AcOH | 69 | 57 | 59 |
| 5 | [Mn(OTf)_2_(^TIPS^mcp)] | CpOH | 60 | 52 | 59 |
| 6 | [Mn(OTf)_2_(^TIPS^mcp)] | DMBOH | 51 | 40 | 40 |
| 7 | [Mn(OTf)_2_(^TIPS^ecp)] | AcOH | 40 | 28 | 60 |
| 8 | [Mn(OTf)_2_(^TIPS^ecp)] | CpOH | 40 | 28 | 56 |
| 9 | [Mn(OTf)_2_(^TIPS^ecp)] | DMBOH | 39 | 30 | 53 |

^a^ Conversions and yields determined by GC analysis of two or three different independent runs. ^b^ ee values determined by GC. ^a^ chloroacetic acid.

**Reaction optimization for oxidation of S2**

**Table S3.** Catalyst screening.

| Entry | Catalyst | Deviation | Conv. (%)^a^ | Yield P2 (%)^a,b^ | Yield SP1 (%)^a,c^ | Yield SP2 (%)^a,d^ | Yield S1+P1 (%)^a^ | ee P2 (%)^e^ |
| --- | --- | --- | --- | --- | --- | --- | --- | --- |
| 1 | [Mn(OTf)_2_(pdp)] |  | 38 | 21 | 6 | 1 | 1 | 29 |
| 2 | [Mn(OTf)_2_(^DMM^pdp)] |  | 50 | 32 | 8 | 1 | 3 | 49 |
| 3 | [Mn(OTf)_2_(^NMe2^pdp)] |  | 42 | 19 | 7 | 3 | 1 | 28 |
| 4 | [Mn(OTf)_2_(^TIPS^pdp)] |  | 56 | 36 | 11 | 2 | 3 | 68 |
| 5 | [Fe(OTf)_2_(^TIPS^pdp)] | 3.5 equiv. H_2_O_2_ | 30 | 5 | 17 | 1 | - | 65 |
| 6 | [Mn(OTf)_2_(^TIPS^mcp)] |  | 61 | 20 | 13 | 5 | 5 | 73 |
| 7 | [Mn(OTf)_2_(^CF3^bpeb)] |  | 52 | 39 | 8 | 1 | 4 | 92 |

^a^ Conversions and yields determined by GC analysis of two or three different independent runs. ^b^ Yield of the isolated product. ^c^ Product identified by GC-MS (CI) [M+H]^+^ m/z = 231.1. ^d^ Product identified by GC-MS (CI) [M+NH_4_]^+^ m/z = 276.1. ^e^ ee values determined by GC.

**Table S4.** Co-ligand screening.

| Entry | Catalyst | Deviation | Conv. (%)^a^ | Yield P2 (%)^a,b^ | Yield SP1 (%)^a,c^ | Yield SP2 (%)^a,d^ | Yield S1+P1 (%)^a^ | ee P2 (%)^e^ |
| --- | --- | --- | --- | --- | --- | --- | --- | --- |
| 1 | [Mn(OTf)_2_(^CF3^bpeb)] | - | 52 | 39 | 8 | 1 | 4 | 92 |
| 2 | [Mn(OTf)_2_(^CF3^bpeb)] | TFE instead of MeCN | 72 | 33 | 3 | 1 | 12 | 92 |
| 3 | [Mn(OTf)_2_(^CF3^bpeb)] | CpOH, 3 equiv. H_2_O_2_ | 66 | 37 | 7 | 2 | 5 | 91 |
| 4 | [Mn(OTf)_2_(^CF3^bpeb)] | BuOH,^f^ 3 equiv. H_2_O_2_ | 52 | 44 | <1 | <1 | <1 | 93 |
| 5 | [Mn(OTf)_2_(^CF3^bpeb)] | EBuOH,^g^ 3 equiv. H_2_O_2_ | 40 | 25 | <1 | <1 | <1 | 96 |
| 6 | [Mn(OTf)_2_(^CF3^bpeb)] | DMBOH, 3 equiv. H_2_O_2_ | 63 | 50 | <1 | <1 | <1 | 98 |
| 7 ^h^ | [Mn(OTf)_2_(^CF3^bpeb)] | DMBOH, 3 equiv. H_2_O_2_ | 99 | 44 | <1 | <1 | 9 | 94 |
| 8 | **[Mn(OTf)_2_(^CF3^bpeb)]** | **DMBOH, 7 equiv. H_2_O_2_, 2 mol%** | **99** | **83 (75) ^i^** | **<1** | **<1** | **<1** | **99** |

^a^ Conversions and yields determined by GC analysis of two or three different independent runs. ^b^ ee values determined by GC. ^c^ Product identified by GC-MS (CI) [M+H]^+^ m/z = 231.1. ^d^ Product identified by GC-MS (CI) [M+NH_4_]^+^ m/z = 276.1. ^e^ Yield of the isolated product. ^f^ Butyric acid. ^g^ 2-Ethylbutyric acid. ^h^ Reaction performed at 0 ºC. ^i^ Yield of the isolated product.

***Scheme S1.*** *GC traces comparing Entry 3 and Entry 6 from Table S4.*

***
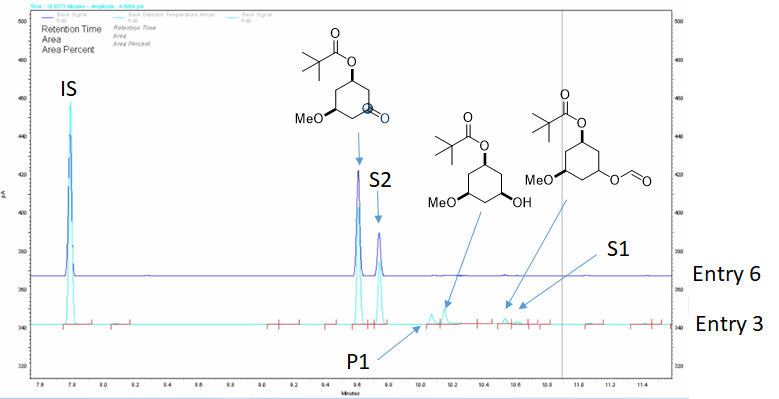
***

**1.5.4. Characterization of oxidation products**

**P1**: The product was obtained following oxidation protocol with 17 equivalents of acetic acid (AcOH), 1 mol% of *(S,S)*-Mn(^TIPS^mcp), and 1 equivalents of H_2_O_2_ (Workup A). The crude product was purified by flash chromatography over silica using hexane:ethyl acetate (1:1) and the product was concentrated to dryness. **P1** was isolated as a white solid (52% yield, 59% ee). ^1^H NMR (400 MHz, CDCl_3_) δ, ppm: 5.23 (tt, J = 7.5, 4.2 Hz, 1H), 4.25 (td, J = 7.2, 3.6 Hz, 1H), 2.69 (td, J = 14.8, 4.6 Hz, 2H), 2.53 (td, J = 14.5, 7.1 Hz, 2H), 2.36 (dt, J = 13.8, 4.0 Hz, 1H), 2.05 (dt, J = 13.9, 7.1 Hz, 1H), 1.18 (s, 9H). ^13^C NMR (101 MHz, CDCl_3_) δ, ppm: 205.6, 177.2, 69.3, 67.2, 49.7, 45.9, 38.7, 37.1, 27.0. HRMS (ESI+) *m/z* calculated for C_11_H_18_O_4_ [M+Na]^+^ 237.1097, found 237.1097. Chiral GC analysis with J&W CYCLOSIL-B. [α]_D_^24^ -56.46 (CHCl_3_, c 0. 15).

**P2**: The product was obtained following the oxidation protocol with 17 equivalents of 2,2-dimethylbutanoic acid (DMBOH), 2 mol% of (*R,R*)-Mn(^CF₃^bpeb), and 7 equivalents of H₂O₂ (Workup A). The crude product was purified by flash chromatography over silica using hexane:ethyl acetate (5:1 → 3:1 → 2:1) and concentrated to dryness. The final product was isolated as a yellow oil (75% yield, 99% ee). ^1^H NMR (400 MHz, CDCl_3_) δ, ppm: 5.07 – 4.97 (m, 1H), 3.60 (tt, *J* = 8.9, 4.4 Hz, 1H), 3.35 (d, *J* = 2.6 Hz, 3H), 2.71 (dddt, *J* = 27.5, 14.5, 5.1, 1.6 Hz, 2H), 2.44 (dddd, *J* = 16.0, 14.4, 9.2, 1.3 Hz, 3H), 1.88 (dt, *J* = 13.1, 9.0 Hz, 1H), 1.18 (s, 9H). ^13^C NMR (101 MHz, CDCl_3_) δ, ppm: 205.2, 177.6, 74.3, 67.4, 56.4, 46.8, 46.1, 38.7, 35.0, 27.0. HRMS(ESI+) *m/z* calculated for C_12_H_20_O_4_ [M+Na]^+^ 251.1254, found 251.1248. Chiral GC analysis with J&W CYCLOSIL-B. [α]_D_^24^ -9.70 (CHCl_3_, c 0.51).

**P3**: The product was obtained following oxidation protocol with 17 equivalents of DMBOH, 2 mol% of *(R,R)*-Mn(^CF3^bpeb), and 3.5 equivalents of H_2_O_2_. (Workup B). The crude product was purified by flash chromatography over silica using pentane:diethyl ether (1:0 → 2:1) and concentrated to dryness. The final product was isolated as a yellow oil (43% yield, 95% ee). ^1^H NMR (400 MHz, CDCl_3_) δ, ppm: 6.89 (ddd, *J* = 10.1, 4.9, 3.5 Hz, 1H), 6.07 (dt, *J* = 10.1, 2.1 Hz, 1H), 3.81 (ddt, *J* = 8.8, 7.1, 4.2 Hz, 1H), 3.37 (s, 3H), 2.85 – 2.64 (m, 2H), 2.54 (ddd, *J* = 16.1, 9.0, 0.7 Hz, 1H), 2.49 – 2.35 (m, 1H). ^13^C NMR (101 MHz, CDCl_3_) δ, ppm: 197.9, 146.4, 130.2, 75.4, 56.2, 43.8, 31.7. Chiral GC analysis with J&W CYCLOSIL-B. [α]_D_^24^ +10.13 (CHCl_3_, c 0.32). Spectral data match those previously reported.^[9]^

**P4:** The product was obtained following oxidation protocol with 17 equivalents of DMBOH, 1 mol% of *(S,S)*-Mn(^CF3^bpeb), and 2 equivalents of H_2_O_2_ (Workup B). The crude product was purified by flash chromatography over silica using pentane:diethyl ether (1:1) and concentrated to dryness. The final product was isolated as a yellow oil (17% yield, 29% ee). ^1^H NMR (400 MHz, CDCl_3_) δ, ppm: 3.69 (tdd, *J* = 6.7, 3.8, 2.7 Hz, 1H), 3.35 (s, 3H), 2.68 – 2.59 (m, 1H), 2.53 – 2.43 (m, 1H), 2.38 – 2.31 (m, 2H), 2.01 – 1.95 (m, 1H), 1.89 – 1.79 (m, 1H), 1.75 – 1.65 (m, 1H). ^13^C NMR (101 MHz, CDCl_3_) δ, ppm: 209.5, 78.2, 56.0, 47.1, 41.1, 29.5, 20.6. Chiral GC analysis with J&W CYCLOSIL-B. Spectral data match those previously reported.^[10]^

**P5:** The product was obtained following oxidation protocol with 17 equivalents of DMBOH, 2 mol% of *(R,R)*-Mn(^CF3^bpeb), and 5 equivalents of H_2_O_2_ (Workup A). The crude product was purified by flash chromatography over silica using hexane:ethyl acetate (1:1) and concentrated to dryness. The final product was isolated as a white solid (65% yield, 99% ee). ^1^H NMR (400 MHz, CDCl_3_) δ, ppm: 6.72 (s, 1H), 4.64 (dq, *J* = 8.9, 4.5 Hz, 1H), 4.00 (td, *J* = 4.0, 2.0 Hz, 1H), 3.36 (s, 3H), 2.74 (ddt, *J* = 14.6, 4.0, 1.9 Hz, 1H), 2.64 – 2.50 (m, 3H), 2.10 (td, *J* = 5.4, 5.0, 3.6 Hz, 2H), 1.92 (s, 3H). ^13^C NMR (101 MHz, CDCl_3_) δ, ppm: 207.1, 169.1, 79.2, 56.7, 47.0, 46.8, 45.1, 33.3, 23.5. HRMS (ESI+) *m/z* calculated for C_9_H_15_NO_3_ [M+Na]^+^ 208.0944, found 208.0944. Chiral GC analysis with J&W CYCLOSIL-B. [α]_D_^24^ +39.58 (CHCl_3_, c 0.31).

**P6**: The product was obtained following oxidation protocol with 17 equivalents of DMBOH, 1.5 mol% of *(R,R)*-Mn(^CF3^bpeb), and 5 equivalents of H_2_O_2_ (Workup A). The crude product was purified by flash chromatography over silica using hexane: ethyl acetate (1:1) and concentrated to dryness. The final product was isolated as a white solid (60% yield, 87% ee). ^1^H NMR (400 MHz, CDCl_3_) δ, ppm: 7.12 (s, 1H), 4.65 (dt, *J* = 8.5, 4.2 Hz, 1H), 4.05 (dd, *J* = 4.6, 2.1 Hz, 1H), 3.38 (s, 3H), 2.83 – 2.72 (m, 1H), 2.60 – 2.50 (m, 3H), 2.14 – 2.05 (m, 2H), 1.13 (s, 9H). ^13^C NMR (101 MHz, CDCl_3_) δ, ppm: 207.1, 177.6, 79.6, 56.8, 47.0, 46.9, 45.2, 45.2, 38.5, 32.8, 27.3. HRMS (ESI+) *m/z* calculated for C_12_H_21_NO_3_ [M+Na]^+^ 250.1414, found 250.1417. Chiral GC analysis with J&W CYCLOSIL-B. [α]_D_^24^ +64.93 (CHCl_3_, c 0.30).

 **P7**: The product was obtained following oxidation protocol with 17 equivalents of DMBOH, 2 mol% of *(S,S)*-Mn(^CF3^bpeb), and 5 equivalents of H_2_O_2_ (Workup A). The crude product was purified by flash chromatography over silica using hexane: ethyl acetate (1:1) and concentrated to dryness. The final product was isolated as a white solid (62% yield, > 99% ee). ^1^H NMR (400 MHz, CDCl_3_) δ, ppm: 7.72 – 7.66 (m, 2H), 7.62 (d, *J* = 8.5 Hz, 1H), 7.52 – 7.45 (m, 1H), 7.45 – 7.38 (m, 2H), 4.91 (dt, *J* = 8.4, 4.3 Hz, 1H), 4.11 (td, *J* = 3.7, 1.8 Hz, 1H), 3.42 (s, 3H), 2.84 (ddt, *J* = 14.6, 3.8, 2.0 Hz, 1H), 2.71 (td, *J* = 4.5, 4.1, 2.1 Hz, 2H), 2.58 (dd, *J* = 14.7, 3.7 Hz, 1H), 2.32 – 2.16 (m, 2H). ^13^C NMR (101 MHz, CDCl_3_) δ, ppm: 206.9, 166.2, 134.4, 131.5, 128.5, 126.9, 79.6, 56.9, 47.4, 47.0, 45.1, 33.1. HRMS(ESI+) *m/z* calculated for C_14_H_17_NO_3_ [M+Na]^+^ 270.1101, found 270.1104. Chiral SFC analysis were done using Chiralpack IC-3. [α]_D_^24^ -68.32 (CHCl_3_, c 0.08). X-ray diffraction quality crystals were obtained by slow evaporation of a solution of **P7** in CH_3_Cl. Absolute configuration of (-)-**P7** could not be unequivocally assigned by X-ray diffraction.

*Solid state structure of (-)-****P7***:


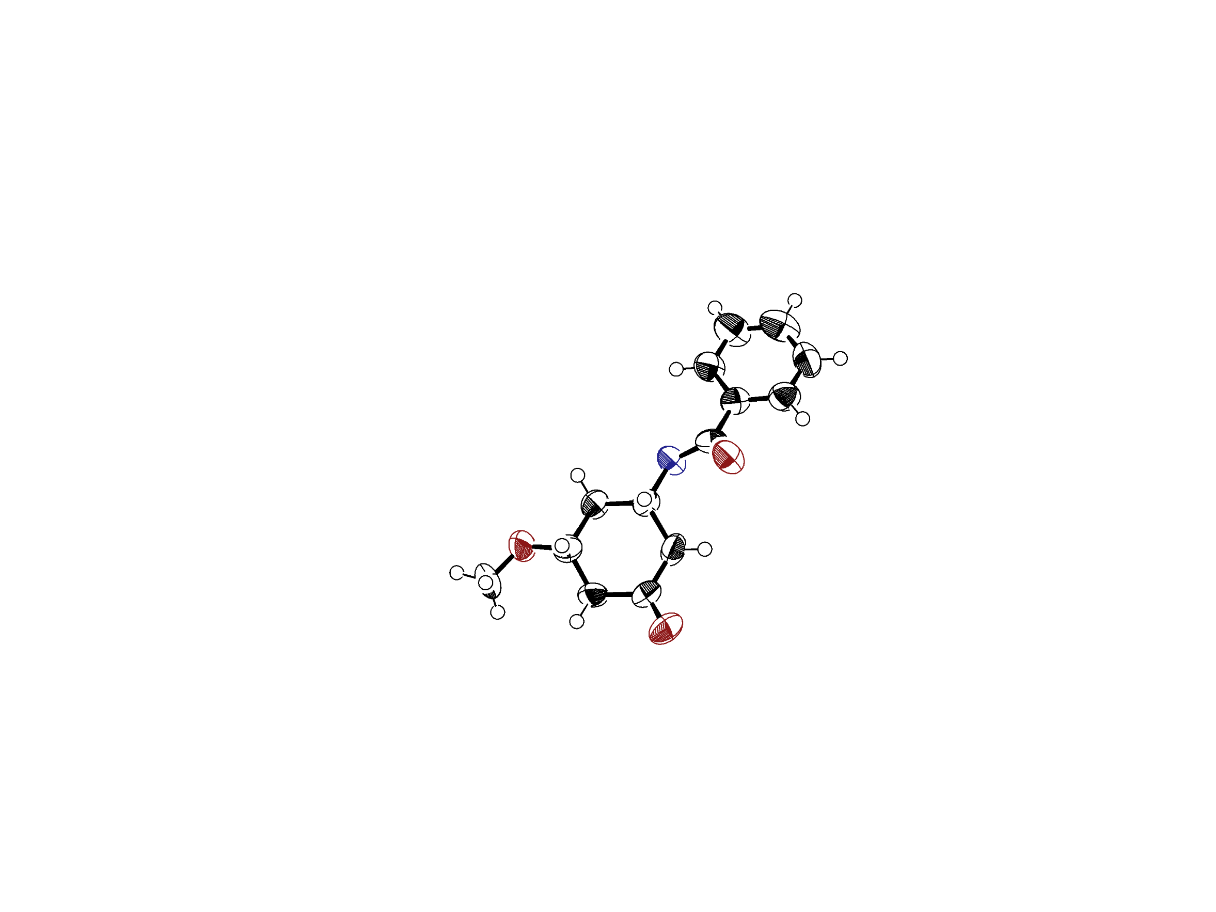


*Crystal data of* ***P7:***

| **Chemical formula** | | C_14_H_17_NO_3_ | | |  |
| --- | --- | --- | --- | --- | --- |
| **Formula weight** | | 247.28 g/mol | | |  |
| **Temperature** | | 130(2) K | | |  |
| **Wavelength** | | 0.71073 Å | | |  |
| **Crystal size** | | 0.080 x 0.080 x 0.570 mm | | |  |
| **Crystal habit** | | colorless needle | | |  |
| **Crystal system** | | orthorhombic | | |  |
| **Space group** | | P 21 21 21 | | |  |
| **Unit cell dimensions** | | a = 9.6113(6) Å | | α = 90° |  |
|  | | b = 10.7851(6) Å | | β = 90° |  |
|  | | c = 12.2839(7) Å | | γ = 90° |  |
| **Volume** | | 1273.33(13) Å^3^ | |  |  |
| **Z** | | 4 | | |  |
| **Density (calculated)** | | 1.290 g/cm^3^ | | |  |
| **Absorption coefficient** | | 0.091 mm^-1^ | | |  |
| **F(000)** | | 528 | | |  |
| **Diffractometer** | | D8 QUEST ECO three-circle diffractometer | | |  |
| **Radiation source** | | Ceramic x-ray tube (Mo Kα, λ = 0.71073 Å) | | |  |
| **Theta range for data collection** | | 3.29 to 27.49° | | |  |
| **Index ranges** | | -12<=h<=12, -14<=k<=14, -15<=l<=15 | | |  |
| **Reflections collected** | | 55943 | | |  |
| **Independent reflections** | | 2924 [R(int) = 0.0871] | | |  |
| **Coverage of independent reflections** | | 99.8% | | |  |
| **Absorption correction** | | Multi-Scan | | |  |
| **Max. and min. transmission** | | 0.9930 and 0.9500 | | |  |
| **Structure solution technique** | | direct methods | | |  |
| **Structure solution program** | | XT, VERSION 2018/2 | | |  |
| **Refinement method** | | Full-matrix least-squares on F2 | | |  |
| **Refinement program** | | SHELXL-2019/1 (Sheldrick, 2019) | | |  |
| **Function minimized** | | Σ w(Fo2 - Fc2)2 | | |  |
| **Data / restraints / parameters** | | 2924 / 0 / 170 | | |  |
| **Goodness-of-fit on F2** | | 1.099 | | |  |
| **Final R indices** | 2561 data; I>2σ(I) | | R1 = 0.0393, wR2 = 0.0909 | | |
|  | all data | | R1 = 0.0514, wR2 = 0.0988 | | |
| **Weighting scheme** | w=1/[σ^2^(F_o_^2^)+(0.0377P)^2^+0.5104P] where P=(F_o_^2^+2F_c_^2^)/3 | | | | |
| **Absolute structure parameter** | 0.1(5) | | | | |
| **Largest diff. peak and hole** | 0.237 and -0.282 eÅ^-3^ | | | | |
| **R.M.S. deviation from mean** | 0.042 eÅ^-3^ | | | | |

***Oxidation of S8***

The yields of **P8** were determined by GC analysis, using biphenyl as an internal standard. Compound **S8** was oxidized following the oxidation protocol with 17 equivalents of DMBOH, 2 mol% of (*S,S*)-Mn(^CF3^bpeb) as a catalyst, and 3.5 equivalents of H_2_O_2_. All attempts to isolate **P8** failed due to degradation during workup. Instead, the product was identified by GC-MS coupled with ^1^H NMR of the crude reaction mixture. To obtain an interpretable ^1^H NMR spectrum, a separate oxidation reaction was conducted using 17 equivalents of acetic acid (AcOH), 1 mol% of (*S,S*)-Mn(^CF3^bpeb), and 1.5 equivalents of H_2_O_2_. The reaction was performed in deuterated acetonitrile, quenched with 3 drops of 2-propanol, and directly submitted to NMR analysis. Only identified and characteristic peaks of **P8** are reported. ^1^H NMR (400 MHz, CD_3_CN) δ, ppm: 3.65 – 3.60 (m, 1H), 3.29 (s, 3H), 2.81 (tdd, *J* = 9.4, 5.4, 4.3 Hz, 1H), 2.69 – 2.62 (m, 2H), 2.61 – 2.51 (m, 1H), 2.50 – 2.31 (m, 4H, **S8 + P8**). Chiral GC analysis with J&W CYCLOSIL-B. GC-MS (CI) [M + NH_4_]^+^ m/z = 204.0.

**P9**: The product was obtained following oxidation protocol with 17 equivalents of DMBOH, 1 mol% of *(S,S)*-Mn(^CF3^bpeb), and 3.5 equivalents of H_2_O_2_ (Workup A). The crude product was purified by flash chromatography over silica using pentate:diethyl ether (1:1) and concentrated to dryness. The product was isolated as a colorless oil (71% yield, 96% ee). ^1^H NMR (400 MHz, CDCl_3_) δ, ppm: 4.15 (q, *J* = 7.1 Hz, 2H), 3.48 (tt, *J* = 10.7, 4.5 Hz, 1H), 3.36 (s, 3H), 2.81 (ddt, *J* = 13.8, 4.8, 2.0 Hz, 1H), 2.46 – 2.35 (m, 3H), 2.35 – 2.23 (m, 2H), 2.22 – 2.11 (m, 1H), 2.07 (td, *J* = 13.1, 12.6, 1.1 Hz, 1H), 1.39 (ddd, *J* = 12.6, 11.4, 10.4 Hz, 1H), 1.26 (t, *J* = 7.1 Hz, 3H). ^13^C NMR (101 MHz, CDCl_3_) δ, ppm: 207.6, 171.6, 77.1, 60.6, 56.3, 47.4, 46.5, 40.7, 36.9, 29.9, 14.2. HRMS (ESI+) *m/z* calculated for C_11_H_18_O_4_ [M+Na]^+^ 237.1097, found 237.1104.Chiral GC analysis with J&W CYCLOSIL-B. [α]_D_^24^ -4.19 (CHCl_3_, c 0.29).

**P10:** The product was obtained following oxidation protocol with 17 equivalents of DMBOH, 1 mol% of *(S,S)*-Mn(^CF3^bpeb), and 5 eq. of H_2_O_2_ (Workup A). The crude product was purified by flash chromatography over silica using hexane:ethyl acetate (2:1) was concentrated to dryness. The product was isolated as a colorless oil (54% yield, 53% ee). ^1^H NMR (400 MHz, CDCl_3_) δ, ppm: 4.09 – 3.99 (m, 2H), 3.48 (tt, *J* = 10.7, 4.5 Hz, 1H), 3.36 (d, *J* = 1.2 Hz, 3H), 2.87 – 2.77 (m, 1H), 2.41 (ddt, *J* = 13.9, 3.9, 1.9 Hz, 1H), 2.36 – 2.23 (m, 2H), 2.16 – 1.95 (m, 5H), 1.42 (ddd, *J* = 12.8, 11.8, 10.5 Hz, 1H). ^13^C NMR (101 MHz, CDCl_3_) δ, ppm: 207.4, 170.9, 77.1, 67.5, 56.3, 47.6, 43.8, 33.8, 32.7, 20.8. Chiral GC analysis with J&W CYCLOSIL-B.

**P11:** The product was obtained following oxidation protocol with 17 equivalents of DMBOH, 1.5 mol% of *(R,R)*-Mn(^CF3^bpeb), and 5.5 equivalents of H_2_O_2_ (Workup A). The crude product was purified by flash chromatography over silica using hexane: ethyl acetate (1:1) and concentrated to dryness. The product was isolated as a colorless oil (40% yield, 20% ee). ^1^H NMR (400 MHz, CDCl_3_) δ, ppm: 7.88 (dd, *J* = 5.5, 3.0 Hz, 2H), 7.76 (dd, *J* = 5.5, 3.0 Hz, 2H), 3.77 – 3.68 (m, 2H), 3.45 (tt, *J* = 10.9, 4.7 Hz, 1H), 3.37 (s, 3H), 2.83 (ddt, *J* = 13.6, 4.2, 2.0 Hz, 1H), 2.42 – 2.27 (m, 3H), 2.25 – 2.11 (m, 2H), 1.52 – 1.40 (m, 1H). ^13^C NMR (101 MHz, CDCl_3_) δ, ppm: 207.1, 168.4, 134.2, 131.8, 123.5, 77.2, 56.3, 47.6, 44.9, 42.8, 35.1, 32.7. HRMS (ESI+) *m/z* calculated for C_16_H_17_NO_4_ [M+Na]^+^ 310.1055, found 310.1052. Chiral SFC analysis were done using Chiralpack IB-3.

**P12:** The product was obtained following oxidation protocol with 17 equivalents of DMBOH, 3 mol% of *(R,R)*-Mn(^CF3^bpeb), and 3.5 equivalents of H_2_O_2_ (Workup A). The crude product was purified by flash chromatography over silica using hexane: ethyl acetate 1:1 and the product was concentrated to dryness. The mixture of product **P12** and substrate **S12** was isolated as white solid (60% yield, 92% ee). ^1^H NMR (400 MHz, Chloroform-d) δ 7.91 – 7.84 (m, 2H), 7.62 – 7.55 (m, 1H), 7.55 – 7.49 (m, 2H), 5.84 (d, J = 9.3 Hz, 1H), 4.06 (dt, J = 9.1, 4.5 Hz, 1H), 3.92 (t, J = 3.9 Hz, 1H), 3.34 (s, 3H), 2.76 – 2.67 (m, 1H), 2.53 – 2.37 (m, 3H), 2.03 – 1.94 (m, 2H). ^13^C NMR (101 MHz, CDCl_3_) δ 205.5, 141.2, 132.6, 129.2, 126.9, 78.6, 56.8, 51.1, 47.7, 45.1, 33.8. HRMS (ESI+) *m/z* calculated for C_13_H_17_NO_4_S [M+K]^+^ 322.0510, found 322.0517. Chiral SFC analysis were done using Chiralpack IA-3.

**P13:** The product was obtained following oxidation protocol with 17 equivalents of DMBOH, 1 mol% of *(R,R)*-Mn(^CF3^bpeb), and 2 equivalents of H_2_O_2_ (Workup A). The crude product was purified by flash chromatography over silica using hexane: ethyl acetate (1:1) and concentrated to dryness. The product was isolated as a yellow oil (62% yield, 98% ee). ^1^H NMR (400 MHz, CDCl_3_) δ, ppm: 7.91 – 7.84 (m, 2H), 7.62 – 7.55 (m, 1H), 7.55 – 7.49 (m, 2H), 5.84 (d, J = 9.3 Hz, 1H), 4.06 (dt, J = 9.1, 4.5 Hz, 1H), 3.92 (t, J = 3.9 Hz, 1H), 3.34 (s, 3H), 2.76 – 2.67 (m, 1H), 2.53 – 2.37 (m, 3H), 2.03 – 1.94 (m, 2H). ^13^C NMR (101 MHz, CDCl_3_) δ, ppm: 205.5, 141.2, 132.6, 129.2, 126.9, 78.6, 56.8, 51.1, 47.7, 45.1, 33.8. HRMS (ESI+) *m/z* calculated for C_14_H_18_O_4_S [M+Na]^+^ 305.0818, found 305.0827. Chiral GC analysis were done using J&W CYCLOSIL-B.

 **P14:** The product was obtained following oxidation protocol with 17 equivalents of DMBOH, 2 mol% of *(R,R)*-Mn(^CF3^bpeb), and 5 equivalents of H_2_O_2_ (Workup A). The crude product was purified by flash chromatography over silica using hexane:ethyl acetate (1:1) and the product was concentrated to dryness. The product was isolated as a yellow oil (52% yield, >99% ee). ^1^H NMR (400 MHz, CDCl_3_) δ, ppm: 7.91 – 7.84 (m, 2H), 7.62 – 7.55 (m, 1H), 7.55 – 7.49 (m, 2H), 5.84 (d, J = 9.3 Hz, 1H), 4.06 (dt, J = 9.1, 4.5 Hz, 1H), 3.92 (t, J = 3.9 Hz, 1H), 3.34 (s, 3H), 2.76 – 2.67 (m, 1H), 2.53 – 2.37 (m, 3H), 2.03 – 1.94 (m, 2H). ^13^C NMR (101 MHz, CDCl_3_) δ, ppm: 207.5, 77.23, 61.70 (dd, *J* = 6.7, 2.6 Hz), 56.26, 47.85 (d, *J* = 9.9 Hz), 47.33, 38.20 (d, *J* = 9.8 Hz), 32.45 (d, *J* = 140.7 Hz), 28.28 (d, *J* = 4.2 Hz), 16.48 (d, *J* = 6.0 Hz).^31^P NMR (162 MHz, CDCl_3_) δ, ppm: 29.02. Chiral GC analysis were done using J&W CYCLOSIL-B. GC-MS (CI) [M – O + H]^+^ m/z = 264.0

# **1.5.5. Derivatization of P2**

A round-bottom flask equipped with a septum and mantained under nitrogen was charged with a 0.10 M solution of (-)-**P2** (51.4 mg, 0.23 mmol, 1 equiv) in dry dichloromethane and hydrogen peroxide • urea (212 mg, 2.3 mmol, 10 equiv). Trifluoroacetic anhydride (130 μl, 0.97 mmol, 4.2 equiv) was added dropwise, and the reaction was stirred at room temperature for 3 h. After completion, the reaction was quenched with saturated NaHCO_3_ and then the aqueous layer was extracted with dichloromethane (3x). The combined organic layers were dried over anhydrous Na_2_SO_4_, filtered, and evaporated under reduced pressure. The crude product was then purified by flash chromatography over silica gel using ether as the eluent, yielding **P2’a** as pure regioisomer (13 mg, 25% yield, white solid) and a mixture of **P2’a** + **P2’b** (regioselectivity= 5:1) as mixture (26 mg, 47% yield) as a colourless oil.

**P2’a**: ^1^H NMR (400 MHz, CDCl_3_) δ, ppm: 5.05 (tdd, *J* = 9.2, 3.9, 2.6 Hz, 1H), 4.31 – 4.21 (m, 2H), 3.51 (dddd, *J* = 8.3, 6.8, 4.1, 2.9 Hz, 1H), 3.39 (s, 3H), 3.02 (dd, *J* = 14.0, 9.4 Hz, 1H), 2.87 (ddd, *J* = 14.1, 2.6, 1.2 Hz, 1H), 2.28 (dt, *J* = 13.6, 4.0 Hz, 1H), 2.07 – 1.97 (m, 2H), 1.19 (s, 9H). ^13^C NMR (101 MHz, CDCl_3_) δ, ppm: 177.3, 170.3, 74.7, 68.6, 65.0, 57.0, 39.7, 38.7, 38.1, 27.0. [α]_D_^24^ +7.10 (CHCl_3_, c 0.15).

**P2’a**+ **P2’b**: ^1^H NMR (400 MHz, CDCl_3_) δ, ppm: 5.10 – 5.00 (m, 1H. **P2’a**), 4.99 – 4.93 (m, 0.25H, **P2’b**), 4.34 – 4.19 (m, 2.30H, **P2’a + P2’b**), 3.62 – 3.56 (m, 0.30H, **P2’b**), 3.54 – 3.47 (m, 1H, **P2’a**), 3.40 - 3.37 (m, 3.40H, **P2’a + P2’b**), 3.02 (dd, *J* = 14.0, 9.4 Hz, 1H, **P2’a**), 2.94 (d, *J* = 5.7 Hz, 0.34H, **P2’b**), 2.87 (ddd, *J* = 13.9, 2.5, 1.2 Hz, 1H, **P2’a**), 2.28 (dt, *J* = 13.7, 4.2 Hz, 1.11H, **P2’a + P2’b**), 2.01 (dt, *J* = 13.6, 8.5 Hz, 1.35H, **P2’a + P2’b**), 1.23 – 1.17 (m, 11H, **P2’a + P2’b**). ^13^C NMR (101 MHz, CDCl_3_) δ, ppm: 177.3 (**P2’b**), 177.3 (**P2’b**), 170.9 (**P2’b**), 170.3 (**P2’a**), 74.7 (**P2’a**), 72.3 (**P2’b**), 68.7 (**P2’b**), 68.6 (**P2’a**), 67.5 (**P2’b**), 65.0 (**P2’a**), 57.0 (**P2’a**), 56.8 (**P2’b**), 39.7 (**P2’a**), 39.4 (**P2’b**), 38.7 (**P2’a**), 38.1 (**P2’a**), 38.0 (**P2’b**), 27.0 (**P2’a**), 27.0 (**P2’b**). HRMS (ESI+) *m/z* calculated for C_12_H_20_O_5_, [M+Na]^+^ 267,1203, found 267.1225.

**1.6. Conformational analysis and V_bur_ (%) calculation of Mn oxo species.**

*i) Conformational search using CREST.*

To uncover the factors governing enantioselectivity in the non-directed oxidation of **S2**, we performed a buried volume (V_bur_ (%)) analysis^[11]^ at the oxo unit of the catalytically active species responsible for HAT.^[12]^ The structural and dynamic features of [Mn^V^O(OCOR)(N_4_L)]^2+^ were explored using the CREST software (v3.0) at the GFN-FF-xTB level, applying several structural constraints—such as the O···O distance, dihedral angles of the chiral backbone, octahedral coordination at Mn, and geometric restraints around the pyridine nitrogen. The lowest-energy conformer obtained under these conditions was selected for analysis (**Figure S2**)


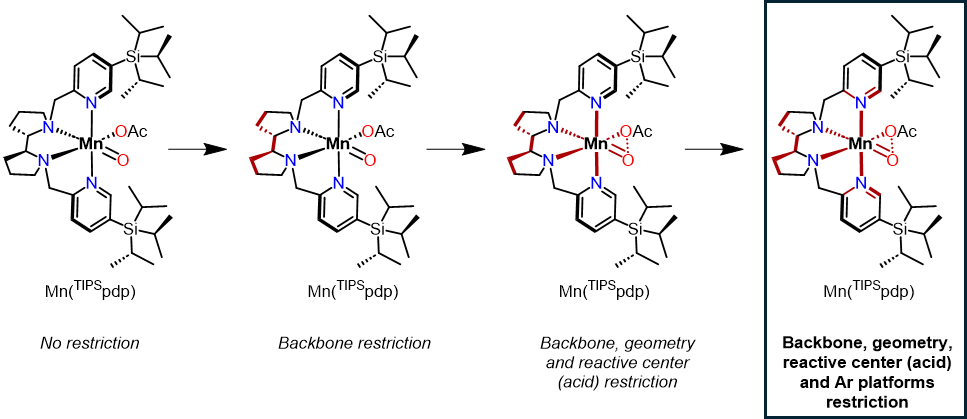


**Figure S2**: Schematic representation of the structural constrains (highlighted in red) applied to preserve the representative geometry of the Mn(V)=oxo intermediates.

The number of conformers and their clustering statistics are summarized in **Figure S3.**


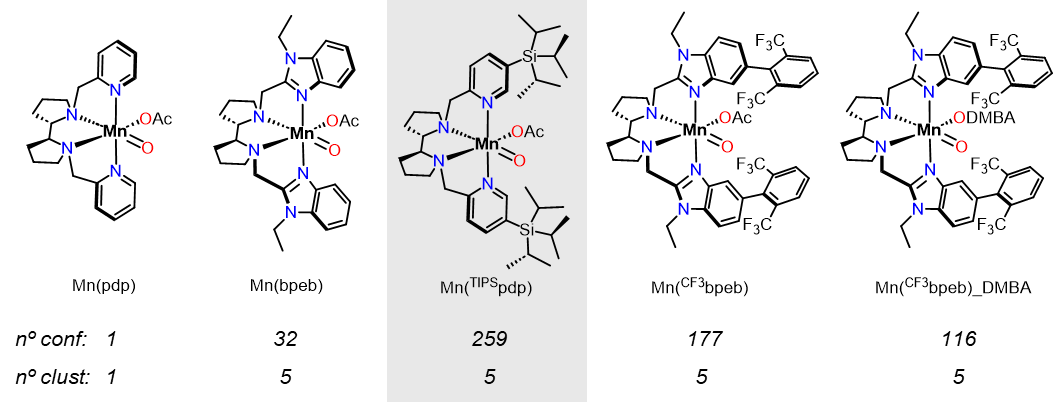


**Figure S3**: Summary of the number of conformers and and clustering results for each Mn(IV)=O intermediate obtained from the CREST analysis.

V_bur_ (%) values were calculated using Morfeus with a 6.5 Å radius centered on the oxo atom. The steric environment was further visualized through 2D steric maps generated with SambVca^[13]^ using a 0.05 mesh resolution, with the Mn=O bond oriented along the z-axis (Figure 3). A linear correlation was observed between V_bur_(%) and enantioselectivity, expressed as ΔΔG^‡^, across the different complexes.

*ii) Buried volume calculation using Morfeus.*

V_bur_ (%) values were calculated using Morfeus with a 6.5 Å radius centered on the oxo atom.

To install Morfeus run the following command:

*conda install -c conda-forge morfeus ml*

In the same folder where crest_best.xyz file is stored. The following bash command can be run for the Vbur calculation:

*burvol --xyz crest_best.xyz --center "x y z" --radius 6.5 --cutoff 2.0 --grid 0.10*

Where x y z is the metal center atom where V_bur_ (%) is calculated, which you would look up manually or by parsing the file.

Check further information in Github:

https://digital-chemistry-laboratory.github.io/morfeus/buried_volume.html

A linear correlation was observed between V_bur_ (%) and enantioselectivity, expressed as ΔΔG^‡^, across the different complexes.

*iii) 2D buried volume representations using SambVca*.

The steric environment was further visualized through 2D steric maps generated with SambVca using a 0.05 mesh resolution, with the Mn=O bond oriented along the z-axis (Figure 3). SambVca^[13]^ can be used on the HTML website that is provided by Luigi Cavallo (developer); or the software version can be installed in the terminal of the computer.

**1.7. Computational details for DFT calculations**

The optimization of reaction intermediates and transition states geometries were performed by using the Gaussian program,^[14]^ at the DFT (B3LYP) level using the LanL2DZ basis set and the pseudopotential for Mn, with 6-31+G(d,p) for the other atoms, incorporating dispersion corrections using the Grimme algorithm GD3BJ.^[15-16]^ Single-point electronic energy corrections of reaction intermediates and transition states were performed by increasing the basis set quality to triple-ζ Def2-TZVPP^[17]^ and adding solvent (acetonitrile) corrections through the Solvation Model based on Density (SMD),^[18]^ including GD3BJ dispersion corrections. All stationary points in the PES were characterized by means of analytical vibrational frequency calculations and connected through IRC calculations. Therefore, the whole methodology of the study can be denoted as B3LYP-GD3BJ/def2-TZVPP/SMD(MeCN)//B3LYP-GB3BJ/6-31G(d,p)(Mn-LANL2DZ).

**Figure S4:** Intrinsic Reaction Coordinate (IRC) plot (empty dots) computed at the B3LYP-GB3BJ/6-31G(d,p)(Mn-LANL2DZ) level of theory, illustrating the reaction pathway from the transition state (**^3^TS-*SR***) to the product (**^3^III-*SR***). The energy profile (in kcal·mol^-1^) is plotted against the reaction coordinate, confirming the TS connectivity between the reactant and product. Colored dots indicate species further analyzed in Table S5, with the following color assignments: **^3^TS-*SR*** (red), **^3^IRC-III^a^** (green), **^3^IRC-III^b^** (orange), **^3^IRC-III^c^** (purple), **^3^IRC-III^d^** (yellow), **^3^IRC-III^e^** (black), **^3^IRC-III^f^** (dark green), **^3^IRC-III^g^** (pale blue), **^3^IRC-III^h^** (grey), and **^3^IRC-III^i^** (pink).

**Figure S5:** Intrinsic Reaction Coordinate (IRC) plot (empty dots) computed at the B3LYP-GB3BJ/6-31G(d,p)(Mn-LANL2DZ) level of theory, illustrating the reaction pathway from the transition state (**^3^TS-*RS***) to the product (**^3^III-*RS***). The energy profile (in kcal·mol^-1^) is plotted against the reaction coordinate, confirming the TS connectivity between the reactant and product.

**Table S5.** Mulliken charges and spin densities of Mn, O (oxo), C_3_ and **S2** (calculated as the sum of all their atomic contributions) computed at the B3LYP-GD3BJ/def2-TZVPP/SMD(MeCN) level of theory. Absolute and relative electronic energies for each species are also provided for comparison.

|  | Mulliken charge | | | Spin density | | | | Elec. Energy  def2-TZVPP (a.u.) | Rel. Elec. Energy def2-TZVPP (kcal·mol^-1^) |
| --- | --- | --- | --- | --- | --- | --- | --- | --- | --- |
|  | **Mn** | **C_3_** | **S2** | **Mn** | **O** | **C_3_** | **S2** |  |  |
| **^3^II** | 0.867 | 0.212 | 0.021 | 2.731 | -0.311 | 0.002 | 0.003 | -5652.242499 | 0.00 |
| **^3^TS-*SR*** | **0.831** | **0.259** | **0.052** | **2.721** | **-0.439** | **-0.003** | **-0.034** | -5652.238191 | 2.70 |
| **^3^IRC-III^a^** | 0.846 | 0.311 | 0.213 | 2.747 | -0.398 | -0.053 | -0.216 | -5652.235027 | 4.70 |
| **^3^IRC-III^b^** | 0.861 | 0.368 | 0.427 | 2.776 | -0.208 | -0.139 | -0.477 | -5652.234921 | 4.76 |
| **^3^IRC-III^c^** | 0.861 | 0.352 | 0.525 | 2.781 | -0.039 | -0.255 | -0.656 | -5652.237457 | 3.16 |
| **^3^IRC-III^d^** | 0.864 | 0.250 | 0.484 | 2.801 | 0.086 | -0.463 | -0.795 | -5652.252947 | -6.56 |
| **^3^IRC-III^e^** | **0.864** | **0.228** | **0.459** | **2.801** | **0.094** | **-0.497** | **-0.802** | -5652.255927 | -8.43 |
| **^3^IRC-III^f^** | 0.872 | 0.255 | 0.664 | 2.477 | 0.096 | -0.338 | -0.503 | -5652.260537 | -11.32 |
| **^3^IRC-III^g^** | 0.871 | 0.304 | 1.066 | 2.030 | 0.055 | 0.002 | 0.0189 | -5652.279865 | -23.45 |
| **^3^IRC-III^h^** | 0.843 | 0.292 | 1.097 | 2.033 | 0.060 | 0.004 | 0.014 | -5652.294559 | -32.67 |
| **^3^IRC-III^i^** | **0.833** | **0.305** | **1.131** | **2.022** | **0.077** | **0.003** | **0.010** | -5652.305955 | -39.82 |
| **^3^III-*SR*** | 0.867 | 0.311 | 1.120 | 2.018 | 0.078 | 0.003 | 0.012 | -5652.307969 | -41.08 |
| **^5^IRC-III^d^** | 0.878 | 0.239 | 0.446 | 2.981 | 0.222 | 0.507 | 0.888 | -5652.247989 | -3.44 |
| **^5^IRC-III^e^** | 0.878 | 0.215 | 0.413 | 3.011 | 0.185 | 0.544 | 0.899 | -5652.251844 | -5.86 |
| **^5^IRC-III^f^** | 0.891 | 0.188 | 0.451 | 3.195 | 0.101 | 0.532 | 0.800 | -5652.255961 | -8.45 |
| **^5^III-*SR*** | 1.219 | 0.376 | 1.141 | 3.902 | 0.020 | 0.003 | 0.016 | -5652.326939 | -52.99 |


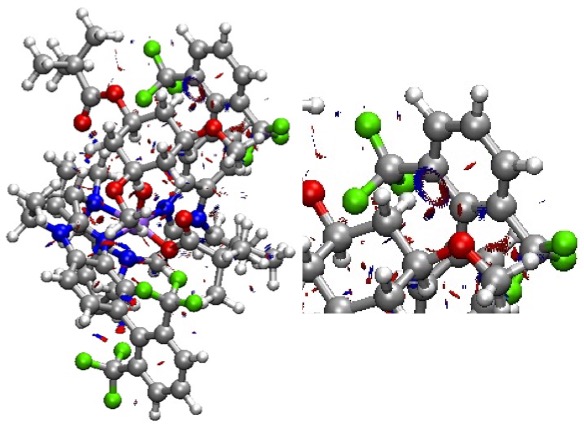


**Figure S6:** Noncovalent interactions in the transition state **^3^TS-*SR*** visualized using the NCOPLOT program.^[19]^ The analysis highlights the C−H···π interaction between the aryl ring of ^CF3^Bzn^R^ and one of the C−H bonds of the C_6_ methylene group of **S2**.

**1.8. Atomic coordinates of DFT-calculated structures**

Mn -0.108558000 1.251278000 0.077090000

O -0.901424000 0.490956000 -1.158229000

O 0.626423000 -0.317589000 0.780741000

N -1.598321000 1.042587000 1.332059000

N -2.332090000 0.737149000 3.400514000

N 0.610742000 2.363263000 1.813390000

N -0.609340000 3.138827000 -0.622346000

N 1.463059000 1.658383000 -1.034471000

N 2.202987000 2.494587000 -2.950896000

C -2.823639000 0.389402000 1.233531000

C -3.517190000 -0.078883000 0.123227000

H -3.115737000 0.034794000 -0.870548000

C -4.712709000 -0.748122000 0.352296000

C -5.208320000 -0.915820000 1.666261000

H -6.146305000 -1.439779000 1.806182000

C -4.520015000 -0.448156000 2.781541000

H -4.910830000 -0.597415000 3.781414000

C -3.305853000 0.197660000 2.541800000

C -1.327527000 1.202010000 2.628236000

C 0.019269000 1.713182000 3.019152000

H -0.021629000 2.393788000 3.876463000

H 0.656151000 0.865146000 3.290311000

C 2.092201000 2.525488000 1.966097000

H 2.502665000 2.721220000 0.975325000

H 2.537941000 1.599027000 2.327919000

C 2.258895000 3.736876000 2.888699000

H 3.225052000 4.222726000 2.742664000

H 2.209124000 3.435080000 3.938580000

C 1.067179000 4.658496000 2.521836000

H 0.578642000 5.061143000 3.410735000

H 1.395886000 5.516099000 1.929545000

C 0.096166000 3.774483000 1.689325000

H -0.922383000 3.793916000 2.084165000

C 0.060791000 4.198301000 0.218856000

H 1.086285000 4.304924000 -0.140873000

C -0.782884000 5.483869000 0.000287000

H -0.242252000 6.213584000 -0.605097000

H -0.989334000 5.964321000 0.959834000

C -2.088850000 5.014438000 -0.696105000

H -2.982784000 5.442195000 -0.239138000

H -2.100750000 5.295979000 -1.751598000

C -2.072153000 3.490805000 -0.563581000

H -2.441416000 3.167773000 0.409252000

H -2.610709000 2.964011000 -1.347981000

C -0.098174000 3.211272000 -2.036423000

H -0.004984000 4.252542000 -2.358213000

H -0.835201000 2.726531000 -2.685708000

C 1.204959000 2.491124000 -2.045217000

C 2.681843000 1.045834000 -1.296906000

C 3.386586000 0.082599000 -0.583935000

H 3.003696000 -0.321157000 0.340246000

C 4.596570000 -0.347700000 -1.114355000

C 5.096324000 0.204737000 -2.315648000

H 6.049331000 -0.146540000 -2.693223000

C 4.402882000 1.178394000 -3.025719000

H 4.807546000 1.593120000 -3.940480000

C 3.171522000 1.580734000 -2.505271000

C -5.436548000 -1.318027000 -0.825369000

C -6.213309000 -0.500475000 -1.666306000

C -6.886012000 -1.040493000 -2.765372000

H -7.490221000 -0.395136000 -3.390738000

C -6.770378000 -2.395638000 -3.058990000

C -5.964507000 -3.209343000 -2.268497000

H -5.832003000 -4.253882000 -2.516702000

C -5.307320000 -2.678789000 -1.157153000

C -2.361769000 0.657928000 4.868690000

H -3.380421000 0.900467000 5.180912000

H -1.711243000 1.445089000 5.257035000

C -1.936174000 -0.719141000 5.374212000

H -0.904231000 -0.943400000 5.089745000

H -2.581729000 -1.501737000 4.968104000

H -2.005423000 -0.749182000 6.464344000

C 5.362337000 -1.407654000 -0.390905000

C 6.141595000 -1.095046000 0.737026000

C 6.876947000 -2.084527000 1.394317000

H 7.479621000 -1.817304000 2.253633000

C 6.838726000 -3.400845000 0.944421000

C 6.038265000 -3.737133000 -0.143605000

H 5.977765000 -4.764153000 -0.481924000

C 5.301871000 -2.751502000 -0.804681000

C 2.241778000 3.250706000 -4.216899000

H 3.131052000 3.887792000 -4.193553000

H 1.368363000 3.906236000 -4.216569000

C 2.232272000 2.338097000 -5.442029000

H 2.083783000 2.947009000 -6.337317000

H 1.442254000 1.586527000 -5.376828000

H 3.180871000 1.809450000 -5.555072000

C 0.261333000 -1.455941000 0.236507000

O -0.340665000 -1.524112000 -0.841653000

C 0.518475000 -2.716486000 1.069275000

H 7.419522000 -4.165018000 1.448699000

H -7.287874000 -2.812277000 -3.915533000

C -4.407161000 -3.580173000 -0.346199000

C -6.270127000 0.989913000 -1.441422000

C 6.146772000 0.306467000 1.300986000

C 4.370033000 -3.166237000 -1.919268000

F 3.075657000 -2.839470000 -1.612230000

F 4.643769000 -2.558990000 -3.093139000

F 4.392466000 -4.488906000 -2.134179000

F 4.932730000 0.605266000 1.864251000

F 6.374872000 1.249337000 0.367106000

F 7.063395000 0.459629000 2.267627000

F -4.805792000 -3.688427000 0.940445000

F -3.126086000 -3.102967000 -0.308956000

F -4.343440000 -4.822279000 -0.849257000

F -5.065649000 1.573721000 -1.743030000

F -6.545255000 1.323942000 -0.167034000

F -7.184062000 1.590099000 -2.225048000

C 0.342540000 -3.964217000 0.191788000

H -0.681114000 -4.041375000 -0.179492000

H 0.568582000 -4.859186000 0.775103000

C 1.934707000 -2.661835000 1.665559000

H 2.685130000 -2.660310000 0.871975000

H 2.078580000 -1.776879000 2.290894000

H 2.108736000 -3.547867000 2.279797000

C -0.587911000 -2.636047000 2.172030000

H -1.562051000 -2.565559000 1.674819000

H -0.442647000 -1.704502000 2.732166000

C -0.605018000 -3.818281000 3.143269000

H -1.396321000 -3.674015000 3.884780000

H -0.810166000 -4.760650000 2.630562000

H 0.339144000 -3.921533000 3.684720000

H 1.024819000 -3.937615000 -0.660400000

H 2.353660000 -1.209118000 -1.327339000

C 2.272567000 -1.232246000 -0.225116000

C 2.827212000 0.083875000 0.338022000

C 0.805088000 -1.413306000 0.160476000

C 1.985782000 1.284087000 -0.119495000

H 3.869805000 0.215319000 0.032917000

H 2.806667000 0.035695000 1.433091000

C -0.022959000 -0.217781000 -0.291645000

H 0.417219000 -2.336186000 -0.280017000

H 0.738498000 -1.513059000 1.249983000

C 0.518394000 1.097601000 0.262742000

H 2.060115000 1.373477000 -1.218477000

H -0.063236000 -0.164471000 -1.384408000

H -0.071216000 1.935812000 -0.115249000

H 0.437754000 1.088421000 1.356046000

O 2.965077000 -2.371122000 0.266576000

O 2.411879000 2.502107000 0.474976000

C 4.250502000 -2.558076000 -0.286550000

H 4.217164000 -2.591058000 -1.387050000

H 4.960336000 -1.773735000 0.012805000

H 4.620147000 -3.517370000 0.083215000

C 3.605995000 3.028573000 -0.063432000

H 3.760852000 4.005951000 0.399569000

H 4.482503000 2.400148000 0.151016000

H 3.534798000 3.160238000 -1.154598000

O -1.370139000 -0.437655000 0.187874000

C -2.369873000 0.170966000 -0.493588000

O -2.184634000 0.859861000 -1.475114000

C -3.730124000 -0.125762000 0.140154000

C -4.822614000 0.565554000 -0.684388000

H -4.667347000 1.646824000 -0.715123000

H -4.826012000 0.203257000 -1.715535000

H -5.803310000 0.365758000 -0.241269000

C -3.954155000 -1.651562000 0.156322000

H -3.949232000 -2.060752000 -0.859275000

H -3.178290000 -2.157740000 0.734793000

H -4.926838000 -1.876775000 0.605492000

C -3.725228000 0.416064000 1.584737000

H -4.695010000 0.222762000 2.054488000

H -2.947173000 -0.064051000 2.182318000

H -3.552837000 1.497215000 1.598677000

Mn 0.462072000 0.679880000 1.331347000

O -0.506321000 0.455803000 0.010486000

O 1.471718000 -0.868812000 1.049878000

N -0.695714000 -0.365090000 2.516884000

N -0.933603000 -1.735382000 4.241981000

N 1.435312000 0.959046000 3.266165000

N -0.348024000 2.545076000 1.740630000

N 1.712421000 1.843917000 0.355174000

N 1.941037000 3.601827000 -0.977820000

C -1.848344000 -1.110305000 2.284724000

C -2.719921000 -1.122442000 1.201537000

H -2.555070000 -0.483591000 0.349271000

C -3.771347000 -2.029544000 1.237698000

C -3.953476000 -2.882770000 2.350839000

H -4.787096000 -3.574854000 2.345684000

C -3.085866000 -2.874490000 3.438724000

H -3.235815000 -3.544969000 4.277016000

C -2.016546000 -1.979081000 3.379161000

C -0.166701000 -0.785248000 3.666870000

C 1.180300000 -0.277417000 4.061422000

H 1.263676000 -0.099268000 5.139007000

H 1.935452000 -1.018835000 3.781503000

C 2.893644000 1.302009000 3.264213000

H 3.060657000 2.011772000 2.453804000

H 3.489624000 0.414859000 3.050423000

C 3.144659000 1.946240000 4.631101000

H 4.008031000 2.613241000 4.608027000

H 3.350655000 1.183977000 5.387627000

C 1.822913000 2.693423000 4.945479000

H 1.503437000 2.533801000 5.976685000

H 1.936268000 3.772672000 4.816215000

C 0.779620000 2.140532000 3.933890000

H -0.126908000 1.783398000 4.428236000

C 0.388709000 3.189610000 2.889958000

H 1.298095000 3.640383000 2.487184000

C -0.595822000 4.245970000 3.461852000

H -0.267658000 5.260200000 3.227549000

H -0.632856000 4.172557000 4.551542000

C -1.973101000 3.925272000 2.820671000

H -2.782295000 3.914803000 3.552799000

H -2.238449000 4.661209000 2.058133000

C -1.789399000 2.550455000 2.176134000

H -1.908530000 1.746869000 2.902194000

H -2.434512000 2.363624000 1.320703000

C -0.163464000 3.365516000 0.492036000

H -0.237225000 4.432899000 0.719501000

H -0.976188000 3.114963000 -0.198236000

C 1.166993000 2.989150000 -0.060306000

C 2.896302000 1.663896000 -0.348750000

C 3.823390000 0.628362000 -0.317356000

H 3.687821000 -0.224739000 0.328857000

C 4.924163000 0.729825000 -1.159148000

C 5.099817000 1.862838000 -1.985837000

H 5.976609000 1.913189000 -2.620568000

C 4.183356000 2.908082000 -2.011260000

H 4.341017000 3.770171000 -2.647257000

C 3.062262000 2.783230000 -1.188755000

C -4.677886000 -2.103267000 0.050933000

C -5.689324000 -1.146749000 -0.151504000

C -6.531936000 -1.222106000 -1.263462000

H -7.313338000 -0.483445000 -1.391628000

C -6.359770000 -2.233090000 -2.203807000

C -5.331308000 -3.157240000 -2.046839000

H -5.161146000 -3.918183000 -2.796661000

C -4.500273000 -3.098695000 -0.926834000

C -0.637748000 -2.497900000 5.464170000

H -1.582523000 -2.623550000 5.998561000

H 0.006964000 -1.879814000 6.093399000

C 0.010660000 -3.845346000 5.152765000

H 0.972663000 -3.714441000 4.649246000

H -0.633455000 -4.450298000 4.509911000

H 0.181877000 -4.395357000 6.081341000

C 5.921652000 -0.382858000 -1.175416000

C 6.894598000 -0.497175000 -0.166713000

C 7.841668000 -1.523718000 -0.200134000

H 8.589020000 -1.584028000 0.581452000

C 7.827066000 -2.456929000 -1.232345000

C 6.844300000 -2.385678000 -2.215567000

H 6.805844000 -3.125399000 -3.005646000

C 5.895789000 -1.360843000 -2.187179000

C 1.636009000 4.855505000 -1.692776000

H 2.447538000 5.559471000 -1.485225000

H 0.727428000 5.259816000 -1.241573000

C 1.444850000 4.639762000 -3.193055000

H 1.051290000 5.557777000 -3.636587000

H 0.759543000 3.813664000 -3.395905000

H 2.390676000 4.411923000 -3.688628000

C 1.102761000 -1.664162000 0.071841000

O 0.290457000 -1.328933000 -0.798385000

C 1.647602000 -3.095673000 0.122573000

H 8.570266000 -3.245791000 -1.262860000

H -7.010265000 -2.286930000 -3.069263000

C -3.367750000 -4.090677000 -0.809449000

C -5.831615000 0.024140000 0.788630000

C 6.893965000 0.450043000 1.010192000

C 4.786787000 -1.369191000 -3.213180000

F 3.565108000 -1.470496000 -2.601839000

F 4.743848000 -0.243850000 -3.957075000

F 4.882585000 -2.406761000 -4.056033000

F 5.808328000 0.219466000 1.815589000

F 6.828053000 1.744350000 0.644735000

F 7.980013000 0.298930000 1.782020000

F -3.467016000 -4.861724000 0.295839000

F -2.157861000 -3.458671000 -0.725310000

F -3.301508000 -4.915258000 -1.865660000

F -4.779246000 0.892929000 0.646454000

F -5.853791000 -0.336239000 2.085144000

F -6.944228000 0.740650000 0.547111000

C 1.400779000 -3.793961000 -1.222506000

H 0.332499000 -3.875915000 -1.431610000

H 1.828018000 -4.798579000 -1.199441000

C 3.148173000 -3.065855000 0.456695000

H 3.706064000 -2.552965000 -0.329825000

H 3.342382000 -2.568678000 1.410628000

H 3.530918000 -4.086834000 0.517821000

C 0.802939000 -3.748580000 1.265761000

H -0.257857000 -3.635124000 1.014845000

H 0.979979000 -3.179990000 2.186629000

C 1.104204000 -5.228137000 1.513597000

H 0.481717000 -5.598162000 2.333597000

H 0.880515000 -5.839426000 0.636496000

H 2.148347000 -5.396100000 1.790769000

H 1.877626000 -3.244885000 -2.037103000

H -0.146956000 0.629827000 -2.247761000

C -0.532473000 0.881647000 -3.245476000

C -0.998805000 -0.410622000 -3.928986000

C -1.694934000 1.861596000 -3.091339000

C -2.144932000 -1.064653000 -3.139822000

H -0.160299000 -1.107209000 -4.008658000

H -1.341376000 -0.179707000 -4.944868000

C -2.812459000 1.206453000 -2.286528000

H -1.353081000 2.774177000 -2.595537000

H -2.070238000 2.140799000 -4.082176000

C -3.296843000 -0.080837000 -2.933835000

H -1.754728000 -1.384999000 -2.162469000

H -2.463199000 1.023543000 -1.267294000

H -4.073515000 -0.539100000 -2.321046000

H -3.739701000 0.147375000 -3.909445000

O 0.490408000 1.561922000 -3.966406000

O -2.717681000 -2.187922000 -3.797563000

C 1.739682000 0.891584000 -3.965461000

H 2.037245000 0.595781000 -2.950412000

H 1.734352000 -0.005173000 -4.596641000

H 2.480159000 1.587880000 -4.363005000

C -1.813596000 -3.242498000 -4.078395000

H -2.414839000 -4.098963000 -4.388192000

H -1.120490000 -2.990094000 -4.890558000

H -1.231503000 -3.523910000 -3.190699000

O -3.969844000 2.080955000 -2.198623000

C -3.952201000 3.056299000 -1.291044000

O -2.987228000 3.273794000 -0.562720000

C -5.216662000 3.908666000 -1.280364000

C -5.556516000 4.250521000 0.181634000

H -5.803842000 3.346989000 0.745146000

H -4.717416000 4.748926000 0.672639000

H -6.421517000 4.918395000 0.210966000

C -4.864717000 5.199902000 -2.056495000

H -4.023675000 5.720428000 -1.589631000

H -4.605733000 4.977899000 -3.096297000

H -5.727870000 5.871255000 -2.058403000

C -6.401634000 3.197548000 -1.950463000

H -7.277436000 3.851924000 -1.922776000

H -6.188316000 2.953420000 -2.992754000

H -6.653244000 2.274283000 -1.423926000

Mn 0.448216000 0.378873000 1.276215000

O -0.599463000 0.450865000 0.003783000

O 1.470794000 -1.066350000 0.795228000

N -0.721799000 -0.818867000 2.309389000

N -0.997693000 -2.397652000 3.840067000

N 1.435998000 0.336271000 3.236040000

N -0.345631000 2.178740000 2.034667000

N 1.683139000 1.695167000 0.506276000

N 1.956088000 3.682828000 -0.435396000

C -1.916221000 -1.465842000 2.011962000

C -2.815097000 -1.278766000 0.969084000

H -2.633575000 -0.537335000 0.208076000

C -3.916566000 -2.123580000 0.913190000

C -4.119697000 -3.113824000 1.901742000

H -4.990861000 -3.753638000 1.827706000

C -3.225770000 -3.303770000 2.951372000

H -3.393129000 -4.076004000 3.693287000

C -2.108322000 -2.467733000 2.981961000

C -0.194334000 -1.415578000 3.377687000

C 1.173906000 -1.013610000 3.813781000

H 1.288568000 -1.025488000 4.902951000

H 1.902561000 -1.709029000 3.385835000

C 2.899117000 0.653446000 3.270202000

H 3.070828000 1.488122000 2.590698000

H 3.475420000 -0.194146000 2.900901000

C 3.178160000 1.052633000 4.722449000

H 4.052535000 1.701044000 4.798799000

H 3.381069000 0.170266000 5.335650000

C 1.873054000 1.754384000 5.178609000

H 1.564850000 1.428447000 6.173478000

H 2.002188000 2.838403000 5.230162000

C 0.807546000 1.393549000 4.104854000

H -0.096563000 0.968419000 4.547152000

C 0.415547000 2.609429000 3.261873000

H 1.325866000 3.112405000 2.928226000

C -0.544278000 3.564055000 4.024096000

H -0.205548000 4.599702000 3.960095000

H -0.565027000 3.306484000 5.085917000

C -1.935726000 3.373187000 3.362155000

H -2.733113000 3.244178000 4.095980000

H -2.203627000 4.232982000 2.743575000

C -1.779118000 2.129047000 2.485354000

H -1.904446000 1.212853000 3.061558000

H -2.438972000 2.102475000 1.621167000

C -0.168133000 3.193644000 0.941092000

H -0.225605000 4.209674000 1.342784000

H -0.991886000 3.071955000 0.231343000

C 1.153202000 2.906308000 0.319086000

C 2.892230000 1.653184000 -0.176310000

C 3.841141000 0.642693000 -0.290306000

H 3.698012000 -0.315064000 0.185457000

C 4.982622000 0.917796000 -1.032393000

C 5.168097000 2.183410000 -1.634353000

H 6.074304000 2.362148000 -2.201250000

C 4.229963000 3.202474000 -1.513122000

H 4.396312000 4.170642000 -1.969853000

C 3.080285000 2.914625000 -0.774865000

C -4.842427000 -1.984741000 -0.252302000

C -5.795757000 -0.951985000 -0.308737000

C -6.639787000 -0.818021000 -1.414186000

H -7.377372000 -0.025375000 -1.429862000

C -6.526534000 -1.692905000 -2.490327000

C -5.559843000 -2.693835000 -2.470578000

H -5.439175000 -3.354991000 -3.318680000

C -4.727632000 -2.843692000 -1.360104000

C -0.718897000 -3.330205000 4.942173000

H -1.659881000 -3.483415000 5.476162000

H -0.035164000 -2.832067000 5.633591000

C -0.140763000 -4.651446000 4.439737000

H 0.816049000 -4.497314000 3.933356000

H -0.824805000 -5.135966000 3.738765000

H 0.021426000 -5.327345000 5.282942000

C 6.025870000 -0.144502000 -1.166424000

C 6.996339000 -0.329506000 -0.165976000

C 7.987748000 -1.303959000 -0.304291000

H 8.730871000 -1.421730000 0.474752000

C 8.020760000 -2.113948000 -1.435706000

C 7.042838000 -1.973644000 -2.416302000

H 7.042699000 -2.618623000 -3.286357000

C 6.050522000 -0.999946000 -2.282385000

C 1.687200000 5.056846000 -0.893695000

H 2.546242000 5.670251000 -0.606362000

H 0.824186000 5.418074000 -0.330346000

C 1.425010000 5.126456000 -2.396438000

H 1.142725000 6.147857000 -2.663578000

H 0.630191000 4.439953000 -2.691362000

H 2.316620000 4.862788000 -2.969314000

C 1.272750000 -1.725697000 -0.357044000

O 0.688435000 -1.237621000 -1.306897000

C 1.787584000 -3.171497000 -0.330943000

H 8.797739000 -2.862059000 -1.546175000

H -7.178211000 -1.584226000 -3.349807000

C -3.673278000 -3.925369000 -1.381156000

C -5.874322000 0.075742000 0.792450000

C 6.944031000 0.480203000 1.108349000

C 4.948149000 -0.935362000 -3.311443000

F 3.737363000 -1.211635000 -2.734265000

F 4.824337000 0.283954000 -3.879384000

F 5.118183000 -1.825415000 -4.298129000

F 5.836846000 0.152801000 1.846183000

F 6.872399000 1.806821000 0.884856000

F 8.007270000 0.256419000 1.894155000

F -3.872259000 -4.850781000 -0.417235000

F -2.424890000 -3.414959000 -1.172199000

F -3.636878000 -4.574165000 -2.555912000

F -4.782758000 0.906456000 0.763667000

F -5.906036000 -0.466989000 2.022922000

F -6.952854000 0.871472000 0.670949000

C 1.712510000 -3.754994000 -1.748594000

H 0.680336000 -3.793963000 -2.104096000

H 2.120142000 -4.768196000 -1.757074000

C 3.238172000 -3.194569000 0.182917000

H 3.888968000 -2.620529000 -0.482582000

H 3.317490000 -2.781995000 1.192590000

H 3.613562000 -4.220165000 0.201644000

C 0.811232000 -3.909343000 0.637173000

H -0.212894000 -3.752840000 0.276862000

H 0.877719000 -3.428550000 1.620284000

C 1.065531000 -5.410927000 0.785991000

H 0.344787000 -5.842672000 1.486546000

H 0.947940000 -5.938270000 -0.163326000

H 2.067229000 -5.623217000 1.170417000

H 2.292622000 -3.149950000 -2.447286000

H -0.232927000 0.904016000 -1.847862000

C -0.504052000 1.311805000 -2.845433000

C -0.917439000 0.113605000 -3.714103000

C -1.660785000 2.290440000 -2.667796000

C -2.125613000 -0.602419000 -3.086432000

H -0.085663000 -0.586559000 -3.799815000

H -1.180617000 0.468396000 -4.718786000

C -2.833318000 1.561533000 -2.016314000

H -1.348127000 3.136565000 -2.050917000

H -1.965301000 2.683693000 -3.644340000

C -3.282906000 0.368757000 -2.845418000

H -1.800577000 -1.041672000 -2.130671000

H -2.543803000 1.247655000 -1.011560000

H -4.103446000 -0.147779000 -2.346691000

H -3.654812000 0.717054000 -3.815279000

O 0.600663000 2.044083000 -3.334726000

O -2.648290000 -1.627240000 -3.916917000

C 1.773518000 1.286047000 -3.617795000

H 1.985102000 0.567455000 -2.820387000

H 1.680267000 0.744483000 -4.565852000

H 2.596900000 1.995683000 -3.697056000

C -1.738811000 -2.672847000 -4.224975000

H -2.327225000 -3.480796000 -4.661877000

H -0.979258000 -2.358561000 -4.951501000

H -1.238073000 -3.045992000 -3.323196000

O -3.977618000 2.439927000 -1.879281000

C -3.977816000 3.296090000 -0.852244000

O -3.035310000 3.399002000 -0.073225000

C -5.229064000 4.163341000 -0.776977000

C -5.611081000 4.328618000 0.705185000

H -5.893007000 3.367876000 1.143756000

H -4.779814000 4.743154000 1.279909000

H -6.464706000 5.006237000 0.790506000

C -4.833896000 5.533786000 -1.376637000

H -4.002298000 5.978428000 -0.822651000

H -4.543853000 5.437171000 -2.427488000

H -5.687500000 6.215060000 -1.322543000

C -6.401012000 3.560899000 -1.566002000

H -7.268906000 4.220948000 -1.482563000

H -6.158506000 3.444644000 -2.623958000

H -6.680394000 2.583659000 -1.165951000

Mn 0.314757000 0.081265000 1.348918000

O -0.700368000 0.415354000 0.090641000

O 1.444178000 -1.215224000 0.707765000

N -0.844258000 -1.339245000 2.072103000

N -1.059781000 -3.267437000 3.145958000

N 1.219848000 -0.344202000 3.325598000

N -0.563006000 1.673403000 2.452545000

N 1.549410000 1.568226000 0.939065000

N 1.862866000 3.734861000 0.575037000

C -1.985590000 -1.956011000 1.574810000

C -2.863673000 -1.557883000 0.576738000

H -2.712975000 -0.627874000 0.055125000

C -3.898605000 -2.424919000 0.248947000

C -4.064739000 -3.646603000 0.940735000

H -4.883938000 -4.298495000 0.662707000

C -3.194792000 -4.045010000 1.952566000

H -3.333741000 -4.989651000 2.465683000

C -2.138577000 -3.182003000 2.249071000

C -0.310560000 -2.155105000 2.979470000

C 1.010837000 -1.799041000 3.572137000

H 1.071594000 -2.050938000 4.636753000

H 1.798269000 -2.345940000 3.046207000

C 2.655435000 0.028364000 3.516740000

H 2.803214000 1.004346000 3.053524000

H 3.302157000 -0.678494000 2.998298000

C 2.842390000 0.100422000 5.036734000

H 3.660060000 0.769719000 5.309620000

H 3.087440000 -0.884227000 5.444293000

C 1.468984000 0.590506000 5.566430000

H 1.133948000 0.002975000 6.423060000

H 1.516777000 1.629999000 5.900277000

C 0.489197000 0.455621000 4.366497000

H -0.420097000 -0.088252000 4.633173000

C 0.096303000 1.820535000 3.800243000

H 1.003361000 2.412620000 3.659133000

C -0.949152000 2.546980000 4.692028000

H -0.639383000 3.569815000 4.914082000

H -1.043745000 2.032731000 5.651712000

C -2.279032000 2.510231000 3.892956000

H -3.127841000 2.209125000 4.509380000

H -2.518199000 3.488517000 3.469461000

C -2.022117000 1.506562000 2.767733000

H -2.163544000 0.479119000 3.102264000

H -2.617283000 1.674353000 1.873703000

C -0.329307000 2.922875000 1.654229000

H -0.411511000 3.807860000 2.292272000

H -1.111909000 3.003112000 0.894884000

C 1.025437000 2.788856000 1.053378000

C 2.790212000 1.700859000 0.327806000

C 3.729929000 0.747485000 -0.048727000

H 3.565157000 -0.299497000 0.139132000

C 4.888877000 1.193359000 -0.669910000

C 5.113577000 2.574800000 -0.871795000

H 6.034732000 2.889140000 -1.347838000

C 4.187363000 3.536219000 -0.484955000

H 4.380438000 4.589904000 -0.644235000

C 3.007665000 3.075447000 0.103810000

C -4.767358000 -2.049245000 -0.906588000

C -5.705841000 -1.004177000 -0.812920000

C -6.474022000 -0.630647000 -1.918913000

H -7.201851000 0.164943000 -1.819081000

C -6.305279000 -1.277278000 -3.138956000

C -5.356437000 -2.288023000 -3.259957000

H -5.199023000 -2.777979000 -4.212601000

C -4.591114000 -2.670932000 -2.156975000

C -0.734966000 -4.436512000 3.975776000

H -1.676884000 -4.811563000 4.382944000

H -0.136659000 -4.088440000 4.821230000

C -0.002246000 -5.513300000 3.177734000

H 0.954048000 -5.141707000 2.798895000

H -0.601248000 -5.844302000 2.325833000

H 0.194016000 -6.378023000 3.816411000

C 5.894568000 0.186030000 -1.126187000

C 6.762238000 -0.441045000 -0.213556000

C 7.722205000 -1.357345000 -0.651454000

H 8.386417000 -1.817597000 0.069686000

C 7.826470000 -1.670229000 -2.002893000

C 6.945038000 -1.096250000 -2.914620000

H 6.994514000 -1.360305000 -3.963694000

C 5.983593000 -0.180163000 -2.482648000

C 1.600870000 5.181587000 0.478851000

H 2.399917000 5.698580000 1.018691000

H 0.668139000 5.370914000 1.014071000

C 1.501373000 5.662000000 -0.968124000

H 1.166237000 6.702164000 -0.976817000

H 0.804306000 5.053139000 -1.547881000

H 2.469613000 5.620168000 -1.470972000

C 1.510860000 -1.628522000 -0.563307000

O 1.109253000 -0.963987000 -1.501007000

C 2.096323000 -3.041461000 -0.704052000

H 8.580341000 -2.371516000 -2.342655000

H -6.904202000 -0.987976000 -3.995157000

C -3.554877000 -3.757023000 -2.341521000

C -5.851223000 -0.205033000 0.459711000

C 6.621221000 -0.205687000 1.271121000

C 4.975477000 0.337106000 -3.483287000

F 3.709953000 -0.008144000 -3.112522000

F 4.985886000 1.684447000 -3.599546000

F 5.175754000 -0.169520000 -4.709646000

F 5.487278000 -0.820032000 1.745156000

F 6.512964000 1.095442000 1.598756000

F 7.647357000 -0.716502000 1.966548000

F -3.902314000 -4.901341000 -1.715976000

F -2.339203000 -3.397236000 -1.848900000

F -3.367684000 -4.052644000 -3.643042000

F -4.794596000 0.659279000 0.614989000

F -5.888327000 -0.965739000 1.566475000

F -6.957824000 0.560562000 0.453808000

C 2.592246000 -3.234501000 -2.145113000

H 1.765061000 -3.216859000 -2.857617000

H 3.103669000 -4.194709000 -2.241994000

C 3.255937000 -3.254650000 0.285772000

H 4.048941000 -2.513113000 0.152950000

H 2.916627000 -3.200287000 1.323307000

H 3.702931000 -4.238596000 0.127804000

C 0.895765000 -3.981861000 -0.376072000

H 0.067919000 -3.738818000 -1.051536000

H 0.545894000 -3.745869000 0.636527000

C 1.199660000 -5.478187000 -0.472464000

H 0.298786000 -6.055808000 -0.245304000

H 1.521751000 -5.762721000 -1.477251000

H 1.977662000 -5.787887000 0.231264000

H 3.290232000 -2.442590000 -2.426000000

H -1.136633000 -0.131350000 -1.699095000

C -1.569660000 0.169253000 -2.700370000

C -2.874659000 0.925466000 -2.462161000

C -0.494410000 1.052290000 -3.353834000

C -2.591671000 2.139185000 -1.590425000

H -3.607601000 0.276196000 -1.980551000

H -3.291420000 1.236302000 -3.426825000

C -0.243572000 2.296435000 -2.487146000

H 0.435966000 0.494764000 -3.460567000

H -0.841127000 1.360111000 -4.349225000

C -1.542017000 3.057221000 -2.210414000

H -2.253715000 1.803125000 -0.610894000

H 0.195144000 1.954078000 -1.538311000

H -1.344657000 3.903088000 -1.545277000

H -1.923361000 3.463890000 -3.153739000

O -1.892695000 -0.996694000 -3.402151000

O 0.640153000 3.211961000 -3.111891000

O -3.839874000 2.843890000 -1.410740000

C -0.833666000 -1.758170000 -3.973682000

H -0.450159000 -1.263885000 -4.874008000

H -0.013664000 -1.869190000 -3.254907000

H -1.264245000 -2.722556000 -4.235560000

C 1.955737000 2.714082000 -3.322990000

H 2.006430000 2.036387000 -4.182603000

H 2.593966000 3.576206000 -3.523218000

H 2.330995000 2.181484000 -2.442137000

C -4.006476000 3.500089000 -0.252230000

O -3.133917000 3.553355000 0.606975000

C -5.353137000 4.202306000 -0.131240000

C -6.375745000 3.681915000 -1.151680000

H -7.318796000 4.220193000 -1.022543000

H -6.034358000 3.829972000 -2.178100000

H -6.571021000 2.617735000 -1.001867000

C -5.872999000 3.989207000 1.303005000

H -6.804233000 4.544962000 1.441143000

H -6.076960000 2.931591000 1.490217000

H -5.146296000 4.340744000 2.038589000

C -5.076013000 5.704832000 -0.373968000

H -4.351113000 6.090320000 0.347994000

H -4.691846000 5.878874000 -1.383950000

H -6.006463000 6.268647000 -0.264398000

Mn 0.467689000 0.272195000 1.232642000

O -0.536631000 0.467014000 -0.250430000

O 1.566293000 -1.227705000 0.602240000

N -0.718377000 -1.113067000 2.073758000

N -1.013033000 -2.804418000 3.490454000

N 1.399912000 -0.014455000 3.155002000

N -0.388267000 1.948866000 2.147949000

N 1.736200000 1.677111000 0.640380000

N 1.979186000 3.774536000 -0.056680000

C -1.952866000 -1.675325000 1.796318000

C -2.902329000 -1.333505000 0.839491000

H -2.706710000 -0.519613000 0.159994000

C -4.062051000 -2.097355000 0.780618000

C -4.267524000 -3.168874000 1.681344000

H -5.182371000 -3.744312000 1.608487000

C -3.324795000 -3.514378000 2.644508000

H -3.499443000 -4.343730000 3.320431000

C -2.155570000 -2.751916000 2.684306000

C -0.186026000 -1.810918000 3.064696000

C 1.180321000 -1.442643000 3.536083000

H 1.307977000 -1.601407000 4.612147000

H 1.923696000 -2.042314000 3.004620000

C 2.844735000 0.344530000 3.290954000

H 3.008705000 1.268639000 2.737096000

H 3.464905000 -0.425795000 2.834191000

C 3.060934000 0.552400000 4.794556000

H 3.894297000 1.229607000 4.989585000

H 3.297831000 -0.394189000 5.287953000

C 1.705781000 1.113563000 5.299564000

H 1.377733000 0.613058000 6.212427000

H 1.778252000 2.178238000 5.535589000

C 0.701029000 0.889168000 4.134690000

H -0.208980000 0.382698000 4.464674000

C 0.309189000 2.206531000 3.459171000

H 1.221086000 2.768785000 3.243871000

C -0.694159000 3.025748000 4.319254000

H -0.350938000 4.051682000 4.465258000

H -0.786046000 2.581895000 5.313954000

C -2.040224000 2.972763000 3.551265000

H -2.887197000 2.760989000 4.206457000

H -2.247969000 3.924514000 3.054873000

C -1.837131000 1.866726000 2.512281000

H -2.008476000 0.878683000 2.940075000

H -2.451249000 1.966259000 1.620897000

C -0.145907000 3.096724000 1.221091000

H -0.215210000 4.061194000 1.737339000

H -0.925903000 3.076269000 0.457774000

C 1.195399000 2.887472000 0.609049000

C 2.939820000 1.746337000 -0.041060000

C 3.899226000 0.763852000 -0.270501000

H 3.759165000 -0.237826000 0.106806000

C 5.035186000 1.129465000 -0.982375000

C 5.189788000 2.451444000 -1.463347000

H 6.086665000 2.701278000 -2.018321000

C 4.237953000 3.440765000 -1.232806000

H 4.388071000 4.449635000 -1.600353000

C 3.107790000 3.069291000 -0.499850000

C -5.059928000 -1.797611000 -0.290811000

C -5.889340000 -0.660941000 -0.229916000

C -6.803793000 -0.383934000 -1.250296000

H -7.446291000 0.483807000 -1.170879000

C -6.892483000 -1.219980000 -2.358559000

C -6.060143000 -2.331056000 -2.452382000

H -6.102431000 -2.974984000 -3.321631000

C -5.154670000 -2.620036000 -1.429789000

C -0.727901000 -3.833162000 4.496592000

H -1.659764000 -4.022516000 5.035510000

H -0.023860000 -3.409443000 5.217192000

C -0.178062000 -5.114238000 3.872332000

H 0.776324000 -4.929376000 3.372132000

H -0.876999000 -5.519544000 3.136012000

H -0.020087000 -5.868181000 4.647704000

C 6.121765000 0.121264000 -1.185160000

C 7.049828000 -0.139339000 -0.160144000

C 8.085224000 -1.058067000 -0.348625000

H 8.791874000 -1.234052000 0.452934000

C 8.210262000 -1.737585000 -1.556488000

C 7.280316000 -1.522256000 -2.568641000

H 7.350979000 -2.067151000 -3.501874000

C 6.244101000 -0.603618000 -2.384657000

C 1.671352000 5.187003000 -0.313118000

H 2.603586000 5.659630000 -0.626382000

H 1.383655000 5.651445000 0.634764000

C 0.581187000 5.367960000 -1.367956000

H 0.466202000 6.429423000 -1.602114000

H -0.385761000 5.000892000 -1.010574000

H 0.843947000 4.838232000 -2.287752000

C 1.446909000 -1.869922000 -0.510836000

O 0.738084000 -1.473038000 -1.465990000

C 2.182066000 -3.214172000 -0.589661000

H 9.020912000 -2.442415000 -1.703273000

H -7.602911000 -1.003188000 -3.148355000

C -4.267695000 -3.835445000 -1.579285000

C -5.767398000 0.326640000 0.904312000

C 6.911769000 0.522790000 1.192244000

C 5.194687000 -0.479549000 -3.458141000

F 3.977040000 -0.907892000 -2.992256000

F 4.999395000 0.792790000 -3.877144000

F 5.472556000 -1.217242000 -4.540365000

F 5.808661000 0.055419000 1.851448000

F 6.772225000 1.860184000 1.110728000

F 7.965973000 0.273141000 1.984308000

F -4.615821000 -4.821196000 -0.725959000

F -2.963219000 -3.547089000 -1.333900000

F -4.327025000 -4.346255000 -2.823953000

F -4.628719000 1.085552000 0.769276000

F -5.701571000 -0.247571000 2.114885000

F -6.790972000 1.203275000 0.925123000

C 2.164054000 -3.736425000 -2.032824000

H 1.142802000 -3.912517000 -2.378882000

H 2.719556000 -4.674430000 -2.104667000

C 3.635945000 -3.039579000 -0.113252000

H 4.175741000 -2.355332000 -0.774064000

H 3.679332000 -2.644860000 0.905471000

H 4.164886000 -3.995240000 -0.133658000

C 1.383731000 -4.156359000 0.361091000

H 0.329627000 -4.138873000 0.053670000

H 1.417997000 -3.722289000 1.367104000

C 1.866977000 -5.606837000 0.412903000

H 1.261329000 -6.180586000 1.121007000

H 1.780644000 -6.099168000 -0.558808000

H 2.908854000 -5.681101000 0.737369000

H 2.631805000 -3.017103000 -2.708716000

H -0.171806000 -0.266321000 -0.818068000

C -0.556514000 1.359778000 -2.949854000

C -0.884473000 0.042125000 -3.530496000

C -1.642120000 2.311233000 -2.614923000

C -2.157269000 -0.622405000 -2.909517000

H -0.038158000 -0.640196000 -3.496145000

H -1.132101000 0.249632000 -4.585549000

C -2.825073000 1.602951000 -1.927338000

H -1.253680000 3.135734000 -2.021950000

H -2.002290000 2.714588000 -3.574461000

C -3.284405000 0.391698000 -2.722679000

H -1.858985000 -1.051157000 -1.945350000

H -2.507273000 1.326858000 -0.923425000

H -4.115642000 -0.090381000 -2.208800000

H -3.649451000 0.704358000 -3.707558000

O 0.629187000 1.810904000 -2.821200000

O -2.646025000 -1.628356000 -3.755832000

C 1.818801000 1.014694000 -3.174301000

H 1.824081000 0.121735000 -2.554036000

H 1.777466000 0.774782000 -4.236547000

H 2.657035000 1.667300000 -2.955601000

C -1.757026000 -2.727000000 -3.963677000

H -2.367071000 -3.554423000 -4.324013000

H -0.996063000 -2.488636000 -4.717650000

H -1.263153000 -3.020311000 -3.031710000

O -3.935583000 2.511245000 -1.838797000

C -3.867916000 3.456043000 -0.875926000

O -2.873709000 3.601710000 -0.180135000

C -5.105014000 4.338883000 -0.791035000

C -5.423803000 4.559768000 0.700304000

H -5.703159000 3.619295000 1.181710000

H -4.563427000 4.979749000 1.225736000

H -6.262769000 5.254108000 0.794756000

C -4.716331000 5.683284000 -1.451621000

H -3.857113000 6.133463000 -0.947153000

H -4.473617000 5.550553000 -2.510881000

H -5.558539000 6.377099000 -1.384209000

C -6.314281000 3.725322000 -1.510781000

H -7.167620000 4.402844000 -1.420541000

H -6.116776000 3.563507000 -2.572468000

H -6.592331000 2.770692000 -1.060164000

Mn 0.440377000 0.167466000 1.171941000

O -0.424385000 0.250435000 -0.409838000

O 1.786356000 -1.360720000 0.625350000

N -0.843770000 -1.166596000 2.023483000

N -1.150692000 -2.874562000 3.413390000

N 1.281544000 -0.091301000 3.133967000

N -0.463545000 2.042661000 2.243600000

N 1.684559000 1.736888000 0.683355000

N 1.912110000 3.847403000 0.026905000

C -2.078956000 -1.720096000 1.729301000

C -3.017941000 -1.357140000 0.773237000

H -2.816218000 -0.537919000 0.103178000

C -4.182865000 -2.111775000 0.694332000

C -4.399546000 -3.195419000 1.577047000

H -5.317211000 -3.764258000 1.489961000

C -3.464593000 -3.561765000 2.541374000

H -3.649210000 -4.399716000 3.203836000

C -2.291866000 -2.806688000 2.599563000

C -0.320792000 -1.878970000 3.014964000

C 1.044258000 -1.524472000 3.499597000

H 1.163193000 -1.693073000 4.574388000

H 1.784801000 -2.122327000 2.962289000

C 2.735726000 0.236602000 3.270852000

H 2.909823000 1.174515000 2.745131000

H 3.332838000 -0.531080000 2.782812000

C 2.962543000 0.392468000 4.779016000

H 3.795461000 1.066172000 4.987169000

H 3.209485000 -0.568761000 5.237709000

C 1.610867000 0.931886000 5.318236000

H 1.274146000 0.368835000 6.190886000

H 1.693427000 1.974525000 5.633458000

C 0.605132000 0.806903000 4.139249000

H -0.326155000 0.320699000 4.438778000

C 0.265257000 2.179142000 3.540426000

H 1.199535000 2.714193000 3.351615000

C -0.684338000 2.977802000 4.483491000

H -0.303851000 3.983575000 4.671797000

H -0.760496000 2.485653000 5.456630000

C -2.052933000 3.008566000 3.755957000

H -2.886169000 2.769017000 4.419477000

H -2.249042000 3.999845000 3.337715000

C -1.901048000 1.981602000 2.626448000

H -2.104078000 0.967412000 2.979119000

H -2.532056000 2.171578000 1.760201000

C -0.176929000 3.191148000 1.356349000

H -0.163280000 4.148912000 1.895291000

H -0.978410000 3.254749000 0.616718000

C 1.138094000 2.945699000 0.688575000

C 2.878546000 1.826553000 -0.017779000

C 3.845142000 0.854952000 -0.265028000

H 3.710234000 -0.152027000 0.098900000

C 4.982725000 1.242182000 -0.964173000

C 5.124389000 2.574512000 -1.422093000

H 6.020422000 2.843984000 -1.968945000

C 4.162250000 3.550754000 -1.177704000

H 4.304617000 4.567200000 -1.526973000

C 3.035886000 3.157994000 -0.449625000

C -5.165868000 -1.783708000 -0.382469000

C -5.974100000 -0.632168000 -0.315445000

C -6.865910000 -0.321788000 -1.346004000

H -7.494662000 0.555464000 -1.262116000

C -6.950698000 -1.137040000 -2.469998000

C -6.138896000 -2.262758000 -2.568361000

H -6.178950000 -2.891757000 -3.448637000

C -5.256526000 -2.586000000 -1.535902000

C -0.856963000 -3.941535000 4.378478000

H -1.785772000 -4.158186000 4.911909000

H -0.152355000 -3.542374000 5.112188000

C -0.301167000 -5.189144000 3.693934000

H 0.638796000 -4.968423000 3.180129000

H -1.009860000 -5.578724000 2.958604000

H -0.114053000 -5.968558000 4.436849000

C 6.093827000 0.257216000 -1.154880000

C 6.950894000 -0.057978000 -0.082796000

C 8.012551000 -0.950639000 -0.251227000

H 8.661562000 -1.168104000 0.587943000

C 8.239136000 -1.549252000 -1.486086000

C 7.381905000 -1.280750000 -2.547924000

H 7.528644000 -1.765474000 -3.505067000

C 6.318979000 -0.388944000 -2.385297000

C 1.596121000 5.262608000 -0.202039000

H 2.532473000 5.754401000 -0.470969000

H 1.270991000 5.697484000 0.747500000

C 0.539126000 5.457531000 -1.287919000

H 0.416477000 6.523138000 -1.498155000

H -0.433645000 5.067418000 -0.973398000

H 0.839618000 4.956497000 -2.212337000

C 1.716458000 -2.023587000 -0.478137000

O 0.949456000 -1.704382000 -1.422179000

C 2.600497000 -3.273083000 -0.586968000

H 9.070555000 -2.232779000 -1.616358000

H -7.643009000 -0.893248000 -3.267961000

C -4.393178000 -3.817802000 -1.689861000

C -5.858841000 0.329546000 0.842245000

C 6.708512000 0.500320000 1.301345000

C 5.340761000 -0.238170000 -3.521462000

F 4.120251000 -0.757399000 -3.170603000

F 5.108397000 1.048300000 -3.870953000

F 5.729837000 -0.885765000 -4.626729000

F 5.610240000 -0.085157000 1.872859000

F 6.482853000 1.827867000 1.308746000

F 7.738873000 0.261875000 2.127663000

F -4.786413000 -4.810890000 -0.864052000

F -3.089628000 -3.565801000 -1.405198000

F -4.432753000 -4.301613000 -2.945044000

F -4.700756000 1.064769000 0.756726000

F -5.838725000 -0.274844000 2.040141000

F -6.863175000 1.227894000 0.855159000

C 2.535811000 -3.840229000 -2.011189000

H 1.519092000 -4.141666000 -2.274692000

H 3.191167000 -4.709133000 -2.106980000

C 4.054103000 -2.894026000 -0.242205000

H 4.441496000 -2.171651000 -0.965410000

H 4.132920000 -2.458818000 0.757353000

H 4.700644000 -3.773664000 -0.285920000

C 2.020850000 -4.276344000 0.453812000

H 0.948790000 -4.401403000 0.249779000

H 2.098120000 -3.809453000 1.443389000

C 2.686615000 -5.653351000 0.489074000

H 2.232341000 -6.272415000 1.268980000

H 2.568562000 -6.186253000 -0.457590000

H 3.755944000 -5.585508000 0.707885000

H 2.863969000 -3.090795000 -2.735703000

H 0.003419000 -0.535119000 -0.879989000

C -0.466992000 1.275818000 -2.756089000

C -0.797009000 -0.028937000 -3.373173000

C -1.543187000 2.238392000 -2.416287000

C -2.116215000 -0.668725000 -2.852656000

H 0.032947000 -0.730092000 -3.297991000

H -0.954454000 0.202810000 -4.441413000

C -2.787265000 1.551249000 -1.838123000

H -1.156566000 3.017701000 -1.764325000

H -1.826932000 2.709998000 -3.371065000

C -3.222853000 0.372711000 -2.695316000

H -1.891736000 -1.137714000 -1.887384000

H -2.561639000 1.250849000 -0.816554000

H -4.100012000 -0.099772000 -2.253578000

H -3.515615000 0.729389000 -3.689439000

O 0.717286000 1.750546000 -2.696978000

O -2.593458000 -1.631422000 -3.757548000

C 1.889605000 0.965332000 -3.110520000

H 1.941119000 0.073419000 -2.490938000

H 1.794120000 0.717649000 -4.167611000

H 2.734168000 1.623981000 -2.941740000

C -1.726257000 -2.747527000 -3.959970000

H -2.340838000 -3.546635000 -4.373471000

H -0.924360000 -2.509096000 -4.670280000

H -1.282743000 -3.085044000 -3.017118000

O -3.887015000 2.480182000 -1.815459000

C -3.859102000 3.434711000 -0.862939000

O -2.900787000 3.578174000 -0.117726000

C -5.088741000 4.331678000 -0.850172000

C -5.478566000 4.572796000 0.621091000

H -5.791538000 3.640663000 1.098227000

H -4.640764000 4.989324000 1.184228000

H -6.313676000 5.276670000 0.666278000

C -4.652869000 5.663982000 -1.505603000

H -3.814883000 6.110866000 -0.963817000

H -4.359394000 5.516311000 -2.549944000

H -5.489500000 6.367514000 -1.487746000

C -6.267146000 3.721647000 -1.622006000

H -7.116881000 4.408456000 -1.582266000

H -6.019141000 3.544102000 -2.670514000

H -6.576716000 2.775398000 -1.174141000

Mn 0.384675000 0.247247000 1.408531000

O -0.688388000 0.523773000 -0.000280000

O 1.448704000 -1.200501000 0.618350000

N -0.755366000 -1.219185000 2.166805000

N -0.965462000 -3.071925000 3.381661000

N 1.333726000 -0.141372000 3.310928000

N -0.467958000 1.866891000 2.408515000

N 1.633535000 1.682892000 0.874723000

N 1.925206000 3.819721000 0.335577000

C -1.911808000 -1.855824000 1.754691000

C -2.808355000 -1.513996000 0.749766000

H -2.637705000 -0.620768000 0.169852000

C -3.865959000 -2.381752000 0.510689000

C -4.033785000 -3.550716000 1.290152000

H -4.872934000 -4.203867000 1.082657000

C -3.142730000 -3.894962000 2.303059000

H -3.283860000 -4.800868000 2.881505000

C -2.063333000 -3.033699000 2.514461000

C -0.208408000 -1.973091000 3.105148000

C 1.137326000 -1.588626000 3.624250000

H 1.252154000 -1.794894000 4.694010000

H 1.902502000 -2.150814000 3.081096000

C 2.771573000 0.237598000 3.466954000

H 2.914248000 1.193261000 2.962628000

H 3.409127000 -0.492298000 2.968599000

C 2.985251000 0.369683000 4.979867000

H 3.801050000 1.057003000 5.210668000

H 3.247918000 -0.595587000 5.421319000

C 1.616976000 0.867438000 5.516245000

H 1.297591000 0.299054000 6.391763000

H 1.665549000 1.914701000 5.824664000

C 0.619964000 0.700701000 4.335333000

H -0.286699000 0.167179000 4.629971000

C 0.218547000 2.052430000 3.735959000

H 1.125377000 2.636564000 3.561481000

C -0.799682000 2.807704000 4.636765000

H -0.471287000 3.828475000 4.841436000

H -0.889036000 2.307664000 5.604725000

C -2.141887000 2.779581000 3.859979000

H -2.987937000 2.516699000 4.497776000

H -2.362364000 3.755176000 3.418551000

C -1.917214000 1.740957000 2.757833000

H -2.071025000 0.725448000 3.125243000

H -2.527414000 1.882962000 1.869059000

C -0.230327000 3.074557000 1.551597000

H -0.300076000 3.999330000 2.134346000

H -1.013012000 3.104204000 0.790215000

C 1.114296000 2.903267000 0.926923000

C 2.841115000 1.776820000 0.205079000

C 3.756560000 0.785696000 -0.128708000

H 3.577317000 -0.240599000 0.149280000

C 4.887265000 1.169342000 -0.837212000

C 5.102345000 2.525886000 -1.174025000

H 6.001331000 2.791719000 -1.718161000

C 4.197776000 3.525852000 -0.828208000

H 4.387931000 4.560117000 -1.089268000

C 3.049011000 3.126883000 -0.139558000

C -4.774517000 -2.076311000 -0.634434000

C -5.693232000 -1.008912000 -0.585619000

C -6.523663000 -0.724055000 -1.673767000

H -7.236251000 0.088269000 -1.602438000

C -6.443398000 -1.485275000 -2.835717000

C -5.516219000 -2.520015000 -2.918687000

H -5.431416000 -3.104503000 -3.826452000

C -4.685266000 -2.810517000 -1.833812000

C -0.650496000 -4.161672000 4.311958000

H -1.589851000 -4.465135000 4.781424000

H -0.018073000 -3.751420000 5.103244000

C 0.026992000 -5.341129000 3.617250000

H 0.990039000 -5.047785000 3.191101000

H -0.599203000 -5.729697000 2.809951000

H 0.200140000 -6.146326000 4.335771000

C 5.866303000 0.110127000 -1.230368000

C 6.822363000 -0.365629000 -0.315773000

C 7.736350000 -1.355567000 -0.684656000

H 8.469077000 -1.697729000 0.035758000

C 7.705648000 -1.895459000 -1.966783000

C 6.738465000 -1.469840000 -2.872312000

H 6.686352000 -1.906051000 -3.862128000

C 5.823044000 -0.479736000 -2.507481000

C 1.643427000 5.244727000 0.118447000

H 2.482651000 5.817748000 0.524146000

H 0.762383000 5.492944000 0.714135000

C 1.412936000 5.571266000 -1.356480000

H 1.106384000 6.615614000 -1.455397000

H 0.643290000 4.930729000 -1.792591000

H 2.324300000 5.432050000 -1.941902000

C 1.292426000 -1.760265000 -0.532690000

O 0.535202000 -1.315063000 -1.428200000

C 2.038432000 -3.089953000 -0.721625000

H 8.422808000 -2.656091000 -2.254390000

H -7.097524000 -1.270935000 -3.673559000

C -3.664509000 -3.917009000 -1.988929000

C -5.761707000 -0.096825000 0.617944000

C 6.829202000 0.129228000 1.112187000

C 4.726494000 -0.116644000 -3.478023000

F 3.494060000 -0.413686000 -2.962208000

F 4.700116000 1.201121000 -3.780601000

F 4.826347000 -0.788673000 -4.636294000

F 5.725472000 -0.321551000 1.783078000

F 6.813984000 1.472149000 1.203952000

F 7.898560000 -0.312615000 1.793056000

F -3.968879000 -5.010104000 -1.269692000

F -2.419761000 -3.520683000 -1.609863000

F -3.553384000 -4.304981000 -3.282315000

F -4.690313000 0.760333000 0.633445000

F -5.754569000 -0.750432000 1.787582000

F -6.860266000 0.683864000 0.594189000

C 2.038376000 -3.503179000 -2.199922000

H 1.032973000 -3.764430000 -2.540735000

H 2.674393000 -4.379024000 -2.349043000

C 3.489601000 -2.955670000 -0.226267000

H 4.040470000 -2.241055000 -0.844300000

H 3.530819000 -2.617078000 0.811875000

H 4.010494000 -3.913353000 -0.296858000

C 1.231261000 -4.106972000 0.141072000

H 0.180862000 -4.063585000 -0.177151000

H 1.253996000 -3.755798000 1.178941000

C 1.716803000 -5.556046000 0.077747000

H 1.098202000 -6.189905000 0.720087000

H 1.653423000 -5.962547000 -0.934900000

H 2.751697000 -5.657449000 0.416794000

H 2.423624000 -2.695841000 -2.826981000

H -0.339121000 -0.135737000 -0.658661000

C -1.908076000 0.051428000 -3.172624000

C -3.092943000 0.788229000 -2.665151000

C -0.657624000 0.750898000 -3.543124000

C -2.707513000 1.990164000 -1.796769000

H -3.776785000 0.103038000 -2.161702000

H -3.615247000 1.163659000 -3.560777000

C -0.369972000 2.039710000 -2.712003000

H 0.188621000 0.063911000 -3.513520000

H -0.800764000 1.075444000 -4.588808000

C -1.643455000 2.846257000 -2.473431000

H -2.339116000 1.640554000 -0.832479000

H 0.042180000 1.715802000 -1.749171000

H -1.408797000 3.699603000 -1.833930000

H -2.018053000 3.240084000 -3.425742000

O -2.089732000 -1.196313000 -3.334861000

O 0.539926000 2.868803000 -3.391112000

O -3.929156000 2.722746000 -1.609605000

C -1.068367000 -2.091513000 -3.910690000

H -0.764028000 -1.696825000 -4.879915000

H -0.248468000 -2.138823000 -3.193116000

H -1.585834000 -3.039774000 -4.010162000

C 1.863353000 2.342994000 -3.478908000

H 1.940568000 1.553621000 -4.235542000

H 2.513055000 3.167482000 -3.772334000

H 2.198678000 1.939909000 -2.517872000

C -4.008396000 3.475097000 -0.486631000

O -3.076252000 3.570580000 0.294245000

C -5.331049000 4.214865000 -0.339272000

C -6.427532000 3.646471000 -1.251603000

H -7.349736000 4.214145000 -1.100930000

H -6.153478000 3.716191000 -2.306482000

H -6.633176000 2.600828000 -1.012763000

C -5.760671000 4.122655000 1.137493000

H -6.665772000 4.715896000 1.291882000

H -5.978920000 3.088619000 1.416170000

H -4.977791000 4.504308000 1.796228000

C -5.039207000 5.688116000 -0.712045000

H -4.263304000 6.108234000 -0.066770000

H -4.716068000 5.778127000 -1.754011000

H -5.949895000 6.280433000 -0.588633000

Mn 0.351584000 0.084790000 1.341140000

O -0.577941000 0.270375000 -0.179873000

O 1.690321000 -1.391948000 0.629105000

N -0.882620000 -1.347021000 2.072942000

N -1.119988000 -3.231635000 3.224379000

N 1.195024000 -0.307645000 3.281267000

N -0.558102000 1.877829000 2.512654000

N 1.584919000 1.693623000 0.938944000

N 1.861228000 3.854147000 0.494867000

C -2.040808000 -1.956535000 1.627025000

C -2.924645000 -1.560352000 0.633630000

H -2.733578000 -0.651812000 0.085430000

C -4.000439000 -2.396751000 0.362960000

C -4.187820000 -3.591253000 1.097822000

H -5.038138000 -4.221177000 0.865773000

C -3.304515000 -3.989992000 2.098891000

H -3.464596000 -4.913537000 2.643454000

C -2.212900000 -3.154398000 2.345776000

C -0.353326000 -2.133869000 2.999809000

C 0.982576000 -1.765342000 3.556172000

H 1.073344000 -1.995912000 4.622536000

H 1.755234000 -2.314693000 3.011145000

C 2.640770000 0.042592000 3.451162000

H 2.793829000 1.017478000 2.989576000

H 3.261950000 -0.674508000 2.916370000

C 2.854185000 0.103723000 4.968572000

H 3.667006000 0.784217000 5.228018000

H 3.124202000 -0.879033000 5.364317000

C 1.484740000 0.569783000 5.532264000

H 1.152472000 -0.067852000 6.353914000

H 1.539493000 1.585698000 5.929719000

C 0.494539000 0.514557000 4.334984000

H -0.434955000 -0.001032000 4.587540000

C 0.147245000 1.923799000 3.828664000

H 1.077733000 2.481892000 3.696635000

C -0.828098000 2.640757000 4.810687000

H -0.466032000 3.636834000 5.072585000

H -0.909717000 2.082165000 5.746840000

C -2.186870000 2.701891000 4.066067000

H -3.024605000 2.398569000 4.697180000

H -2.395533000 3.717571000 3.718762000

C -1.999628000 1.765928000 2.864999000

H -2.189442000 0.723978000 3.136423000

H -2.619960000 2.011711000 2.004916000

C -0.269401000 3.095985000 1.718772000

H -0.268217000 4.003237000 2.337355000

H -1.062138000 3.219116000 0.975605000

C 1.057331000 2.906840000 1.050675000

C 2.789518000 1.829908000 0.264493000

C 3.719328000 0.864600000 -0.105472000

H 3.552251000 -0.173301000 0.133708000

C 4.855561000 1.292379000 -0.780053000

C 5.055437000 2.664355000 -1.059153000

H 5.957104000 2.964523000 -1.580474000

C 4.134408000 3.637151000 -0.681318000

H 4.313249000 4.683999000 -0.896353000

C 2.986729000 3.194843000 -0.017777000

C -4.906836000 -2.020623000 -0.763099000

C -5.784897000 -0.922355000 -0.665685000

C -6.604599000 -0.560513000 -1.738845000

H -7.287089000 0.273239000 -1.631125000

C -6.553328000 -1.272707000 -2.932856000

C -5.668936000 -2.339597000 -3.061056000

H -5.608353000 -2.889203000 -3.992328000

C -4.849304000 -2.708282000 -1.991531000

C -0.805157000 -4.373668000 4.092106000

H -1.743487000 -4.700843000 4.547063000

H -0.167612000 -4.009705000 4.901531000

C -0.133028000 -5.508719000 3.321862000

H 0.820141000 -5.183719000 2.896334000

H -0.770473000 -5.857391000 2.505210000

H 0.057222000 -6.350499000 3.992419000

C 5.869561000 0.269179000 -1.181134000

C 6.782045000 -0.246276000 -0.243099000

C 7.730538000 -1.201911000 -0.616026000

H 8.427165000 -1.576160000 0.123944000

C 7.780893000 -1.666601000 -1.926430000

C 6.857037000 -1.203464000 -2.858140000

H 6.863607000 -1.585369000 -3.871443000

C 5.906514000 -0.248789000 -2.489335000

C 1.572409000 5.285289000 0.337855000

H 2.419925000 5.843973000 0.746224000

H 0.703822000 5.512358000 0.959234000

C 1.313374000 5.663271000 -1.120096000

H 1.020596000 6.714528000 -1.179147000

H 0.522434000 5.049847000 -1.556807000

H 2.207584000 5.526742000 -1.731909000

C 1.615878000 -1.926690000 -0.538147000

O 0.862638000 -1.491694000 -1.447692000

C 2.473740000 -3.179114000 -0.771727000

H 8.525451000 -2.399613000 -2.216091000

H -7.198061000 -0.996598000 -3.759802000

C -3.887854000 -3.859244000 -2.192684000

C -5.824162000 -0.063010000 0.577762000

C 6.700409000 0.152549000 1.212311000

C 4.841040000 0.128730000 -3.488334000

F 3.603315000 -0.246746000 -3.043336000

F 4.774143000 1.459792000 -3.718692000

F 5.019151000 -0.470751000 -4.676451000

F 5.587949000 -0.390659000 1.798441000

F 6.619122000 1.483578000 1.393803000

F 7.755344000 -0.289238000 1.915514000

F -4.276414000 -4.971122000 -1.543514000

F -2.633827000 -3.562881000 -1.760906000

F -3.766129000 -4.175792000 -3.502081000

F -4.728658000 0.762174000 0.630099000

F -5.833205000 -0.769502000 1.716687000

F -6.899858000 0.748224000 0.592578000

C 2.391675000 -3.614948000 -2.240415000

H 1.380739000 -3.938188000 -2.505841000

H 3.071404000 -4.448773000 -2.431455000

C 3.937170000 -2.860645000 -0.409524000

H 4.336323000 -2.093779000 -1.077428000

H 4.030937000 -2.503142000 0.618588000

H 4.563718000 -3.748282000 -0.524472000

C 1.883580000 -4.262274000 0.177889000

H 0.806961000 -4.347360000 -0.024822000

H 1.977489000 -3.886985000 1.203712000

C 2.521879000 -5.648866000 0.078731000

H 2.059289000 -6.331018000 0.798830000

H 2.389978000 -6.087424000 -0.913704000

H 3.593227000 -5.622502000 0.296201000

H 2.674614000 -2.789427000 -2.898298000

H -0.101989000 -0.389062000 -0.772903000

C -1.775771000 0.071519000 -3.010286000

C -2.975984000 0.794595000 -2.520656000

C -0.526157000 0.779464000 -3.370442000

C -2.626532000 2.019093000 -1.674938000

H -3.653530000 0.104802000 -2.015452000

H -3.497326000 1.140943000 -3.428613000

C -0.277910000 2.108737000 -2.593119000

H 0.327768000 0.103184000 -3.290454000

H -0.641660000 1.049180000 -4.435358000

C -1.573957000 2.881284000 -2.359786000

H -2.272977000 1.696543000 -0.697010000

H 0.165646000 1.830642000 -1.630281000

H -1.361034000 3.752650000 -1.737335000

H -1.956935000 3.249350000 -3.318974000

O -1.954873000 -1.173965000 -3.195075000

O 0.580240000 2.954003000 -3.318953000

O -3.863698000 2.736615000 -1.528331000

C -0.946654000 -2.062308000 -3.806260000

H -0.644318000 -1.637074000 -4.763335000

H -0.115844000 -2.150255000 -3.103199000

H -1.480524000 -2.998005000 -3.936308000

C 1.918710000 2.476148000 -3.435136000

H 1.998027000 1.648269000 -4.149026000

H 2.520563000 3.307875000 -3.801381000

H 2.309551000 2.142107000 -2.469008000

C -3.978815000 3.515034000 -0.427683000

O -3.069733000 3.633953000 0.377104000

C -5.308084000 4.250890000 -0.332003000

C -6.367503000 3.679783000 -1.285041000

H -7.295483000 4.246604000 -1.171278000

H -6.052879000 3.747411000 -2.328744000

H -6.580652000 2.634499000 -1.052131000

C -5.792473000 4.157760000 1.128021000

H -6.703038000 4.750460000 1.248724000

H -6.020693000 3.123594000 1.398237000

H -5.034696000 4.539890000 1.815165000

C -5.005955000 5.724870000 -0.693383000

H -4.255546000 6.146243000 -0.019627000

H -4.644559000 5.815995000 -1.722657000

H -5.922057000 6.314888000 -0.603852000

**1.9. NMR spectra**

^1^H-NMR of **S1** in CDCl_3_

^13^C-NMR of **S1** in CDCl_3_

^1^H-NMR of **S2** in CDCl_3_

^13^C-NMR of **S2** in CDCl_3_

^1^H-NMR of **S3** in CDCl_3_

^13^C-NMR of **S3** in CDCl_3_

^1^H-NMR of **S5** in CDCl_3_

^13^C-NMR of **S5** in CDCl_3_

^1^H-NMR of **S6** in CDCl_3_

^13^C-NMR of **S6** in CDCl_3_

^1^H-NMR of **S7** in CDCl_3_

^13^C-NMR of **S7** in CDCl_3_

^1^H-NMR of **S8** in CDCl_3_

^13^C-NMR of **S8** in CDCl_3_

^1^H-NMR of **S9** in CDCl_3_

^13^C-NMR of **S9** in CDCl_3_

^1^H-NMR of **B1** in CDCl_3_

^13^C-NMR of **B1** in CDCl_3_

^1^H-NMR of **S10** in CDCl_3_

^13^C-NMR of **S10** in CDCl_3_

^1^H-NMR of **S11** in CDCl_3_

^13^C-NMR of **S11** in CDCl_3_

^1^H-NMR of **S12** in CDCl_3_

^12^C-NMR of **S12** in CDCl_3_

^1^H-NMR of **S13** in CDCl_3_

^13^C-NMR of **S13** in CDCl_3_

^1^H-NMR of **S14** in CDCl_3_

^13^C-NMR of **S14** in CDCl_3_

^31^P-NMR of **S14** in CDCl_3_

^1^H-NMR of **P1** in CDCl_3_

^13^C-NMR o f**P1** in CDCl_3_

^1^H-NMR of **P2** in CDCl_3_

^13^C-NMR of **P2** in CDCl_3_

^1^H-NMR of **P3** in CDCl_3_

^13^C-NMR of **P3** in CDCl_3_

^1^H-NMR of **P4** in CDCl_3_

^13^C-NMR of **P4** in CDCl_3_

^1^H-NMR of **P5** in CDCl_3_

^13^C-NMR of **P5** in CDCl_3_

^1^H-NMR of **P6** in CDCl_3_

^13^C-NMR of **P6** in CDCl_3_

^1^H-NMR of **P7** in CDCl_3_

^13^C-NMR of **P7** in CDCl_3_

^1^H-NMR of **P8+S8** reaction mixture in CD_3_CN

^1^H-NMR of **P8+S8** reaction mixture in CD_3_CN (peaks highlighted)


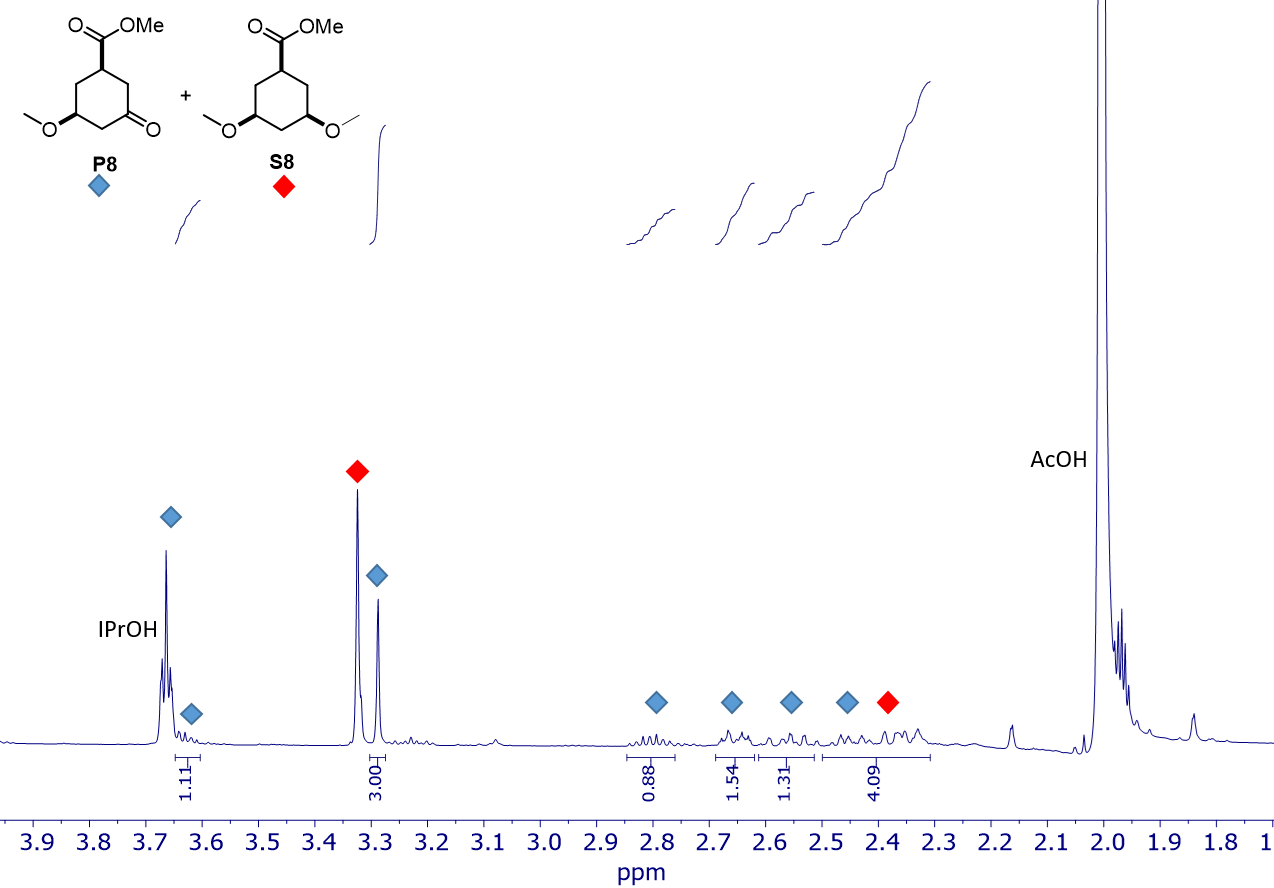


^1^H-NMR of **P9** in CDCl_3_

^13^C-NMR of **P9** in CDCl_3_

^1^H-NMR of **P10** in CDCl_3_

^13^C-NMR of **P10** in CDCl_3_

^1^H-NMR of **P11** in CDCl_3_

^13^C-NMR of **P11** in CDCl_3_

^1^H-NMR of **P12** + **S12** in CDCl_3_

^13^C-NMR of **P12** + **S12** in CDCl_3_

^1^H-NMR of **P13** in CDCl_3_

+

^13^C-NMR of **P13** in CDCl_3_

^1^H-NMR of **P14** in CDCl_3_

^13^C-NMR of **P14** in CDCl_3_

^31^P-NMR of **P14** in CDCl_3_

^1^H-NMR of **P2’** in CDCl_3_

^13^C-NMR of **P2’** in CDCl_3_

COSY-NMR of **P2’** in CDCl_3_


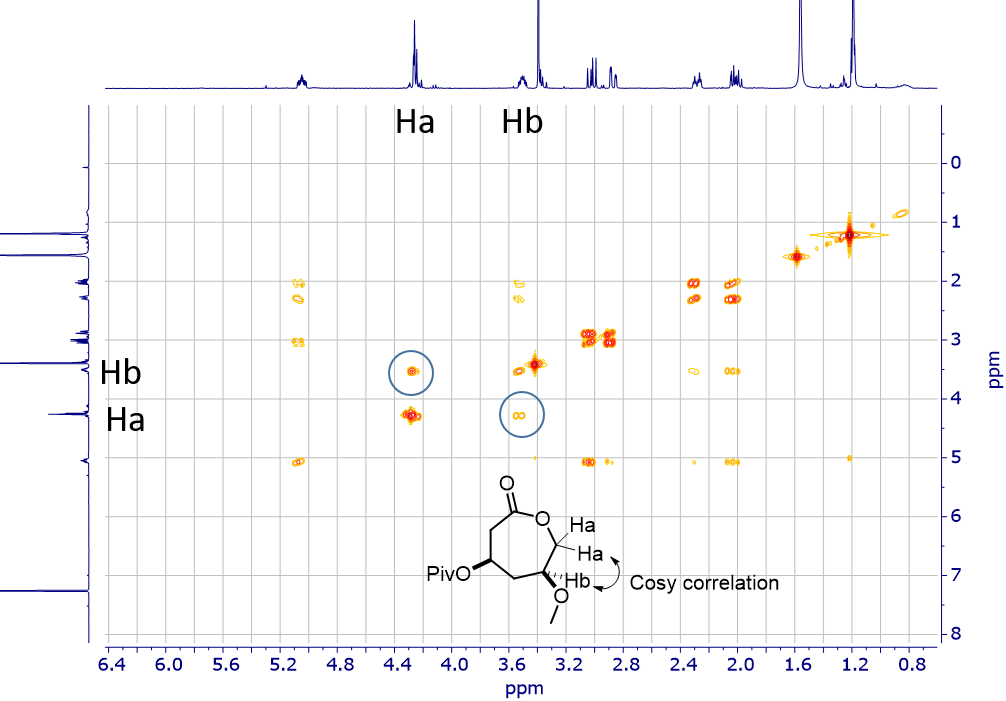


^1^H-NMR of **P2’a** + **P2’b** in CDCl_3_

^13^C-NMR of **P2’a + P2’b** in CDCl_3_

# **2.0. SFC and GC traces**

The racemic products were obtained by substrate oxidations with rac-Mn (^TIPS^mcp) complex.

**Rac-P1**


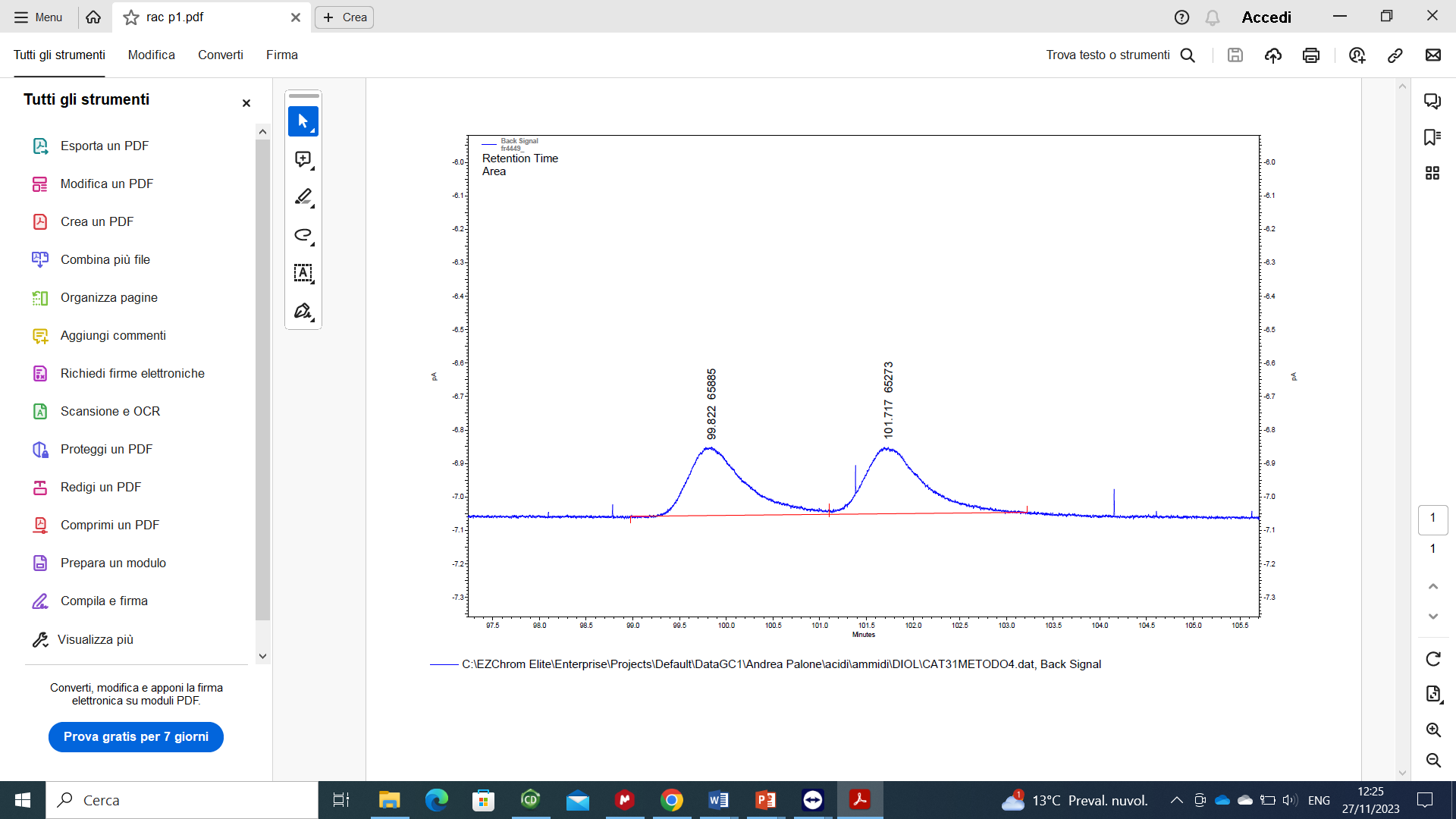


**Chiral-P1**


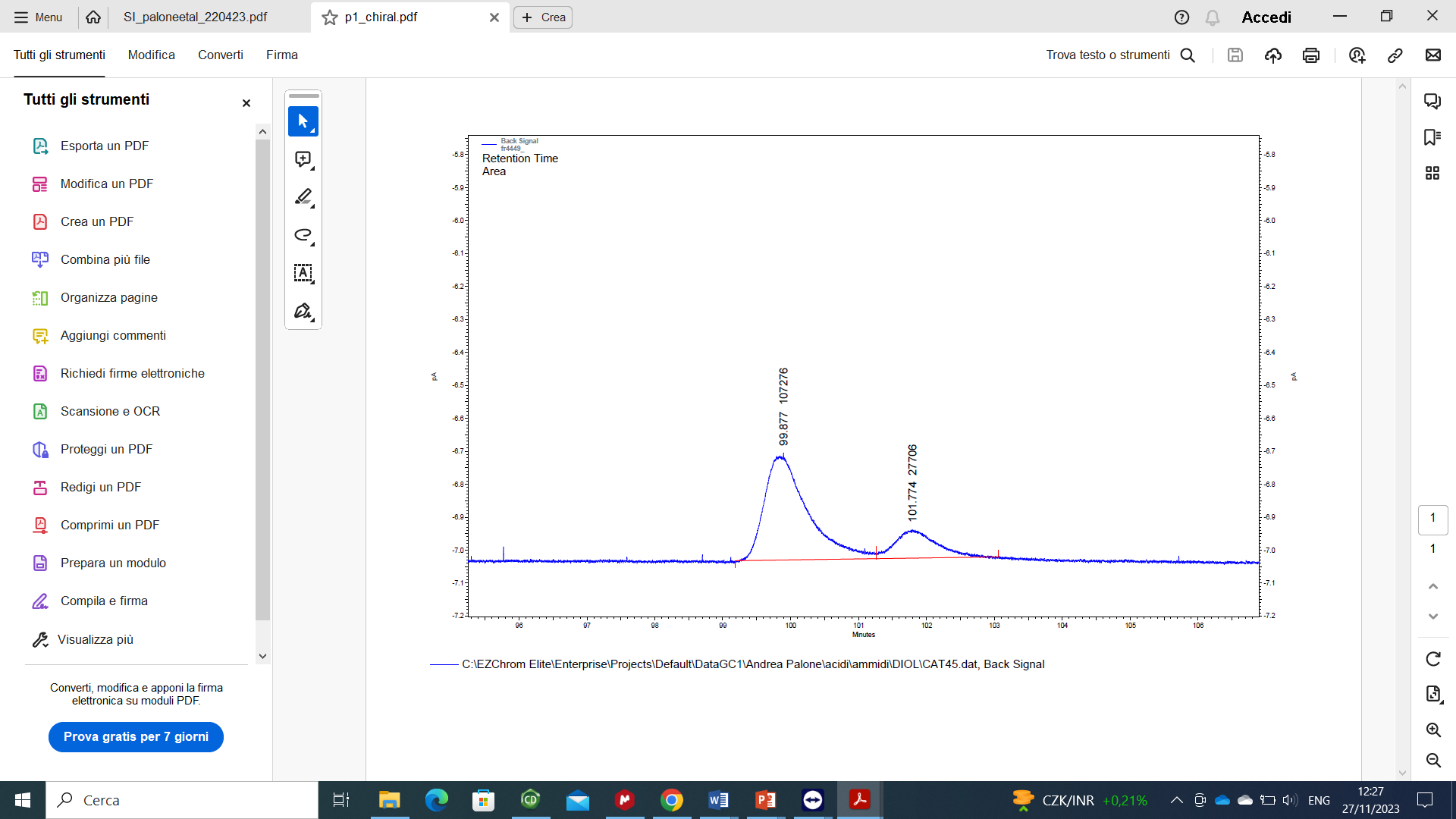


**Rac-P2**


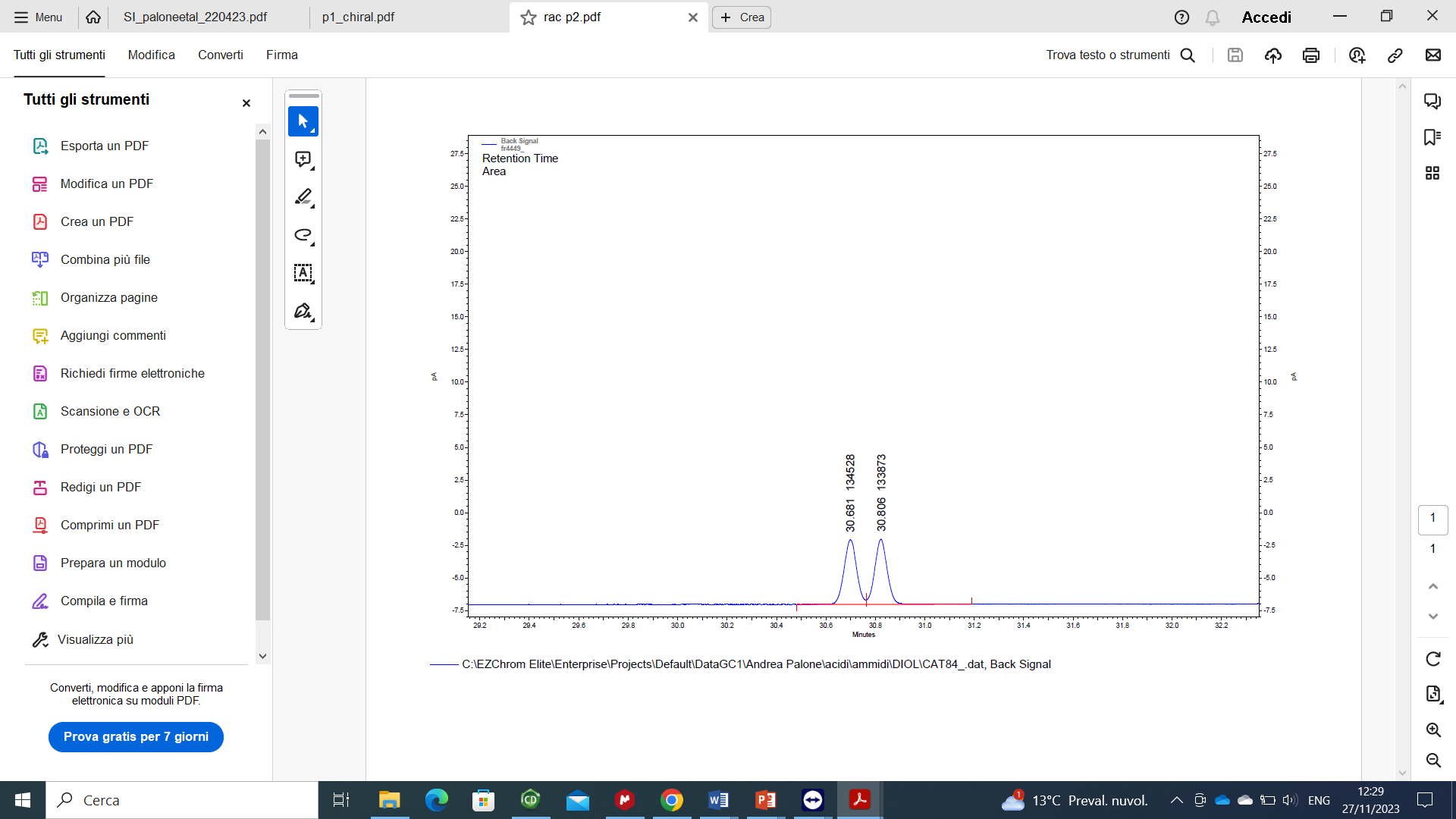


**Chiral-P2**


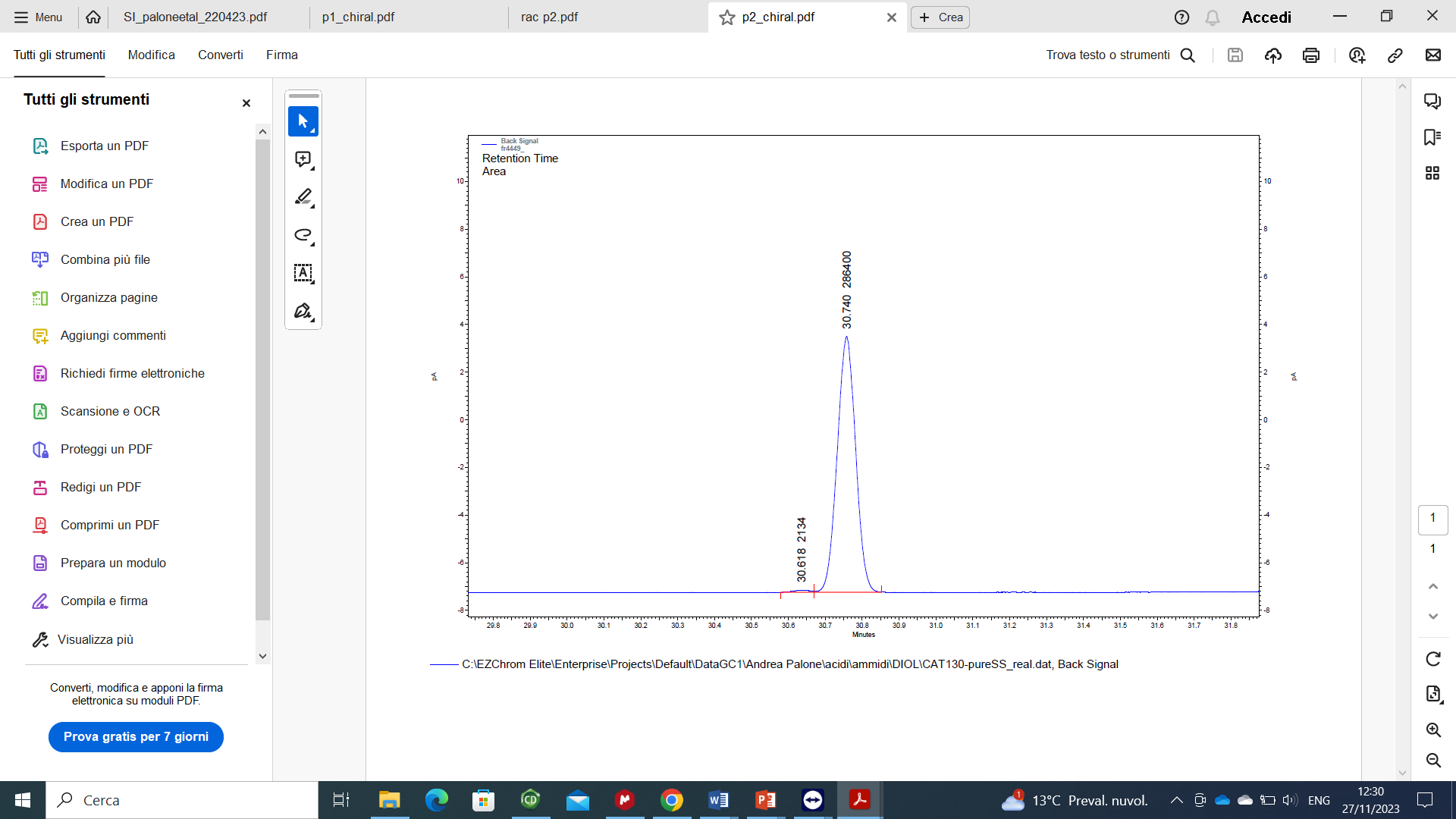


**Rac-P3**


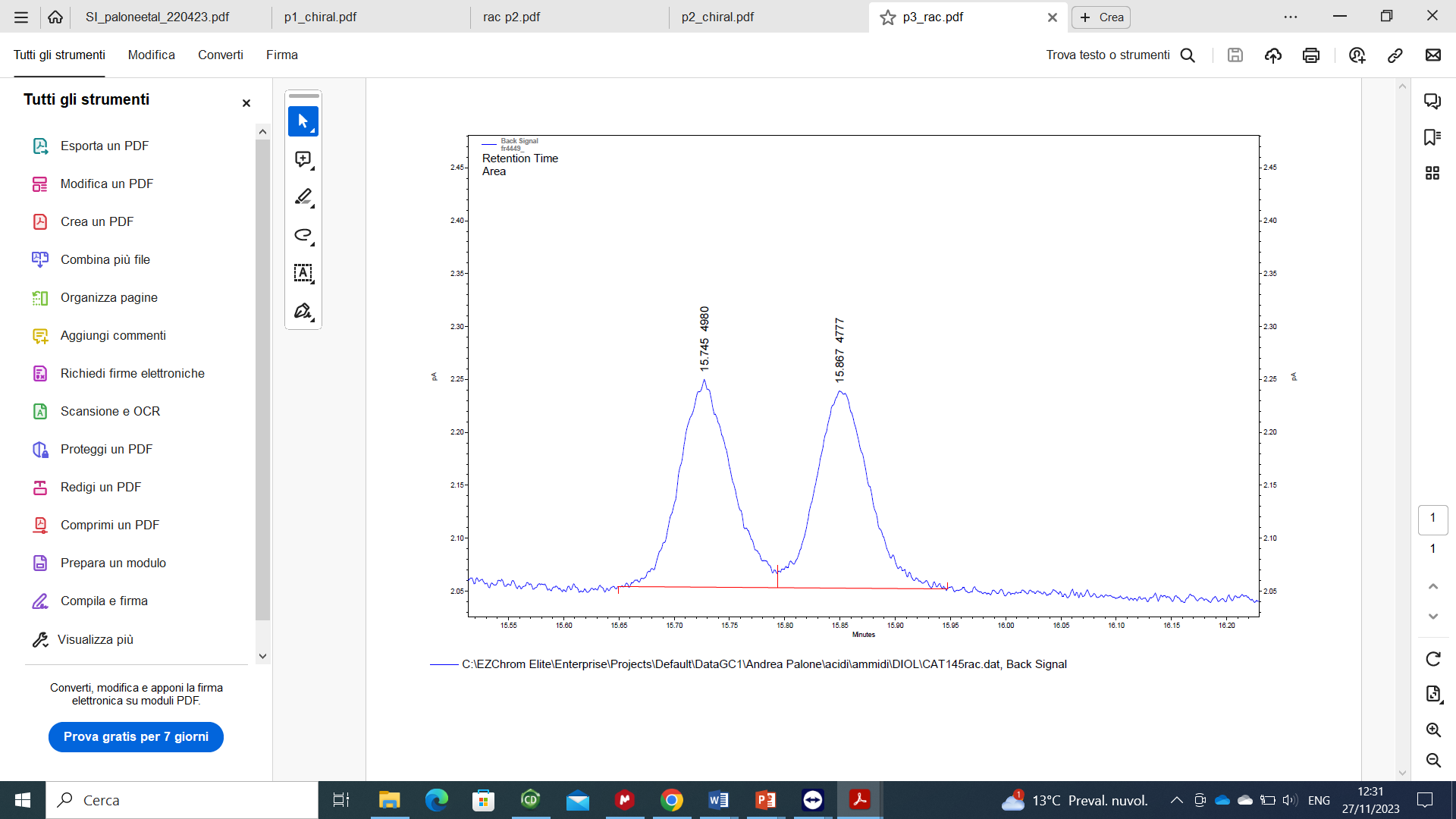


**Chiral-P3**


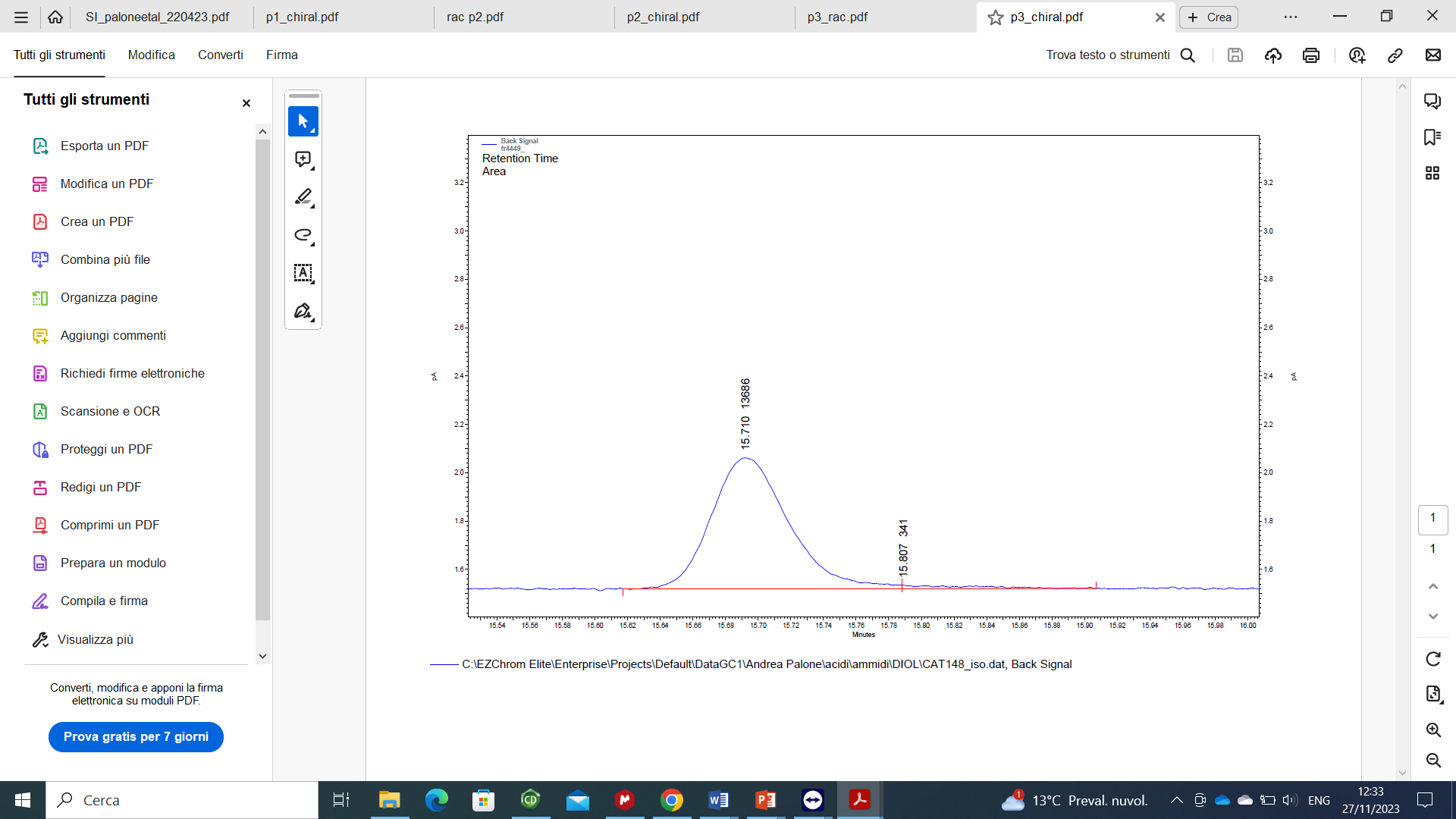


**Rac-P4**


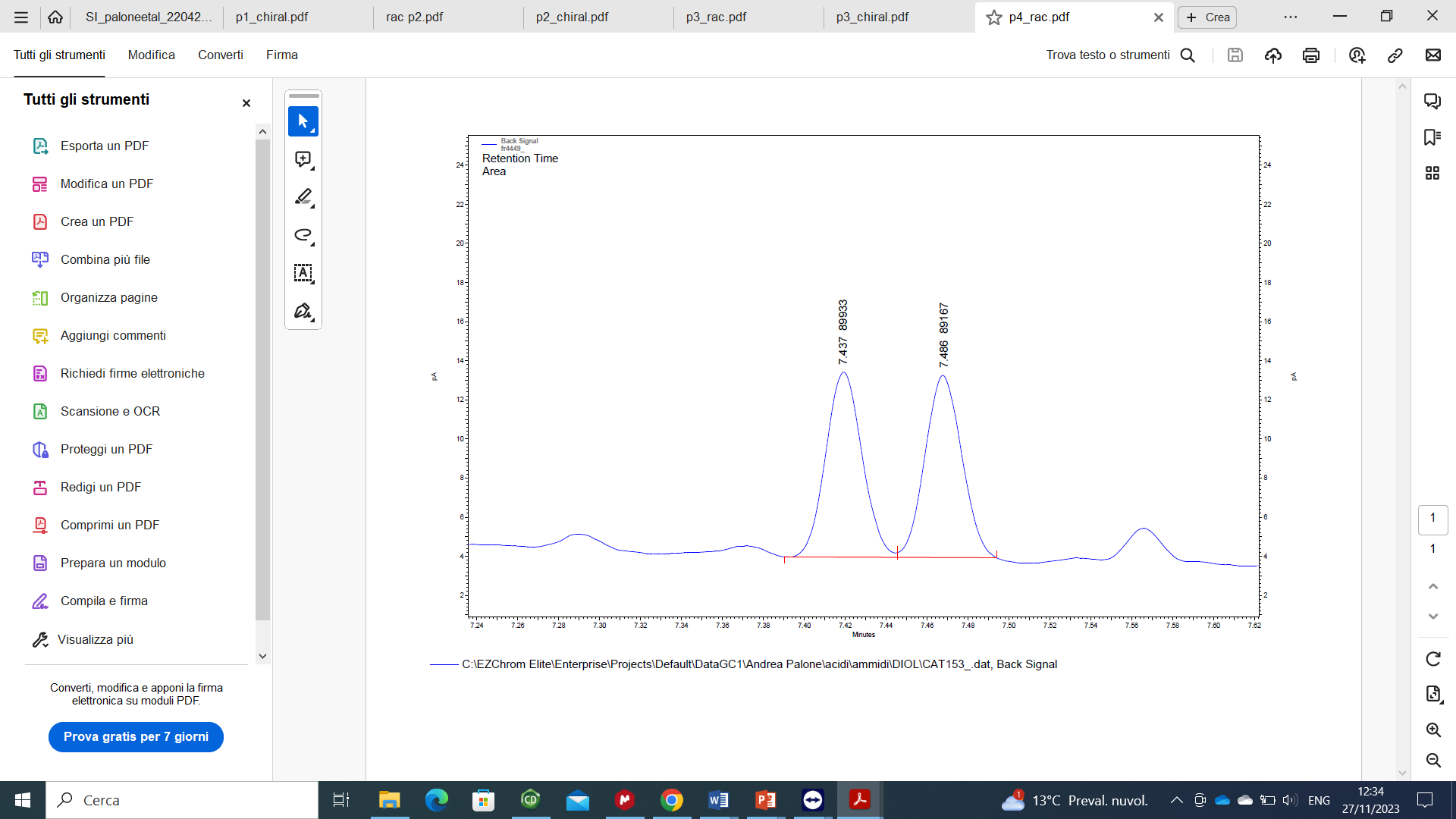


**Chiral-P4**


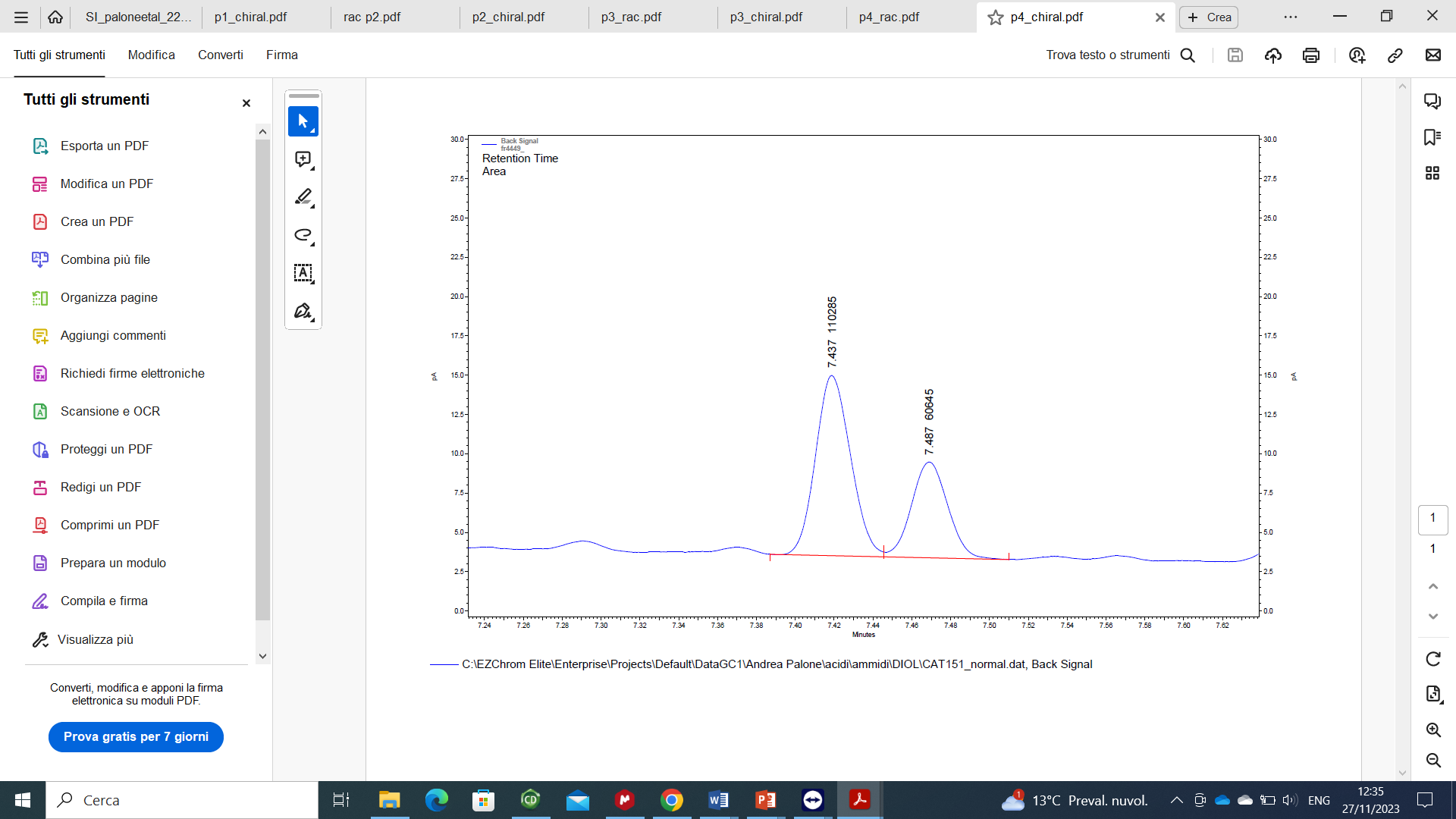


**Rac-P5**


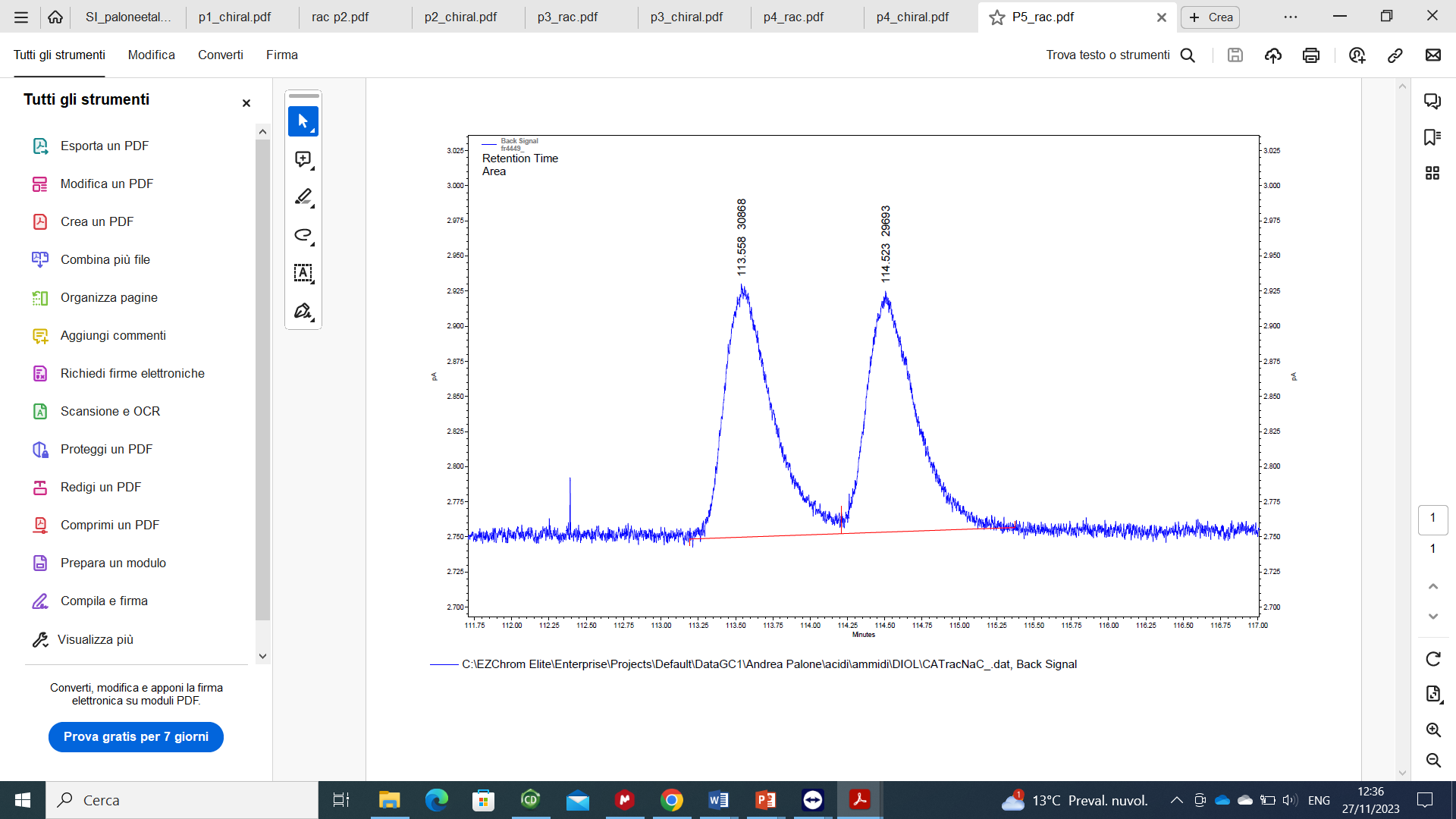


**Chiral-P5**


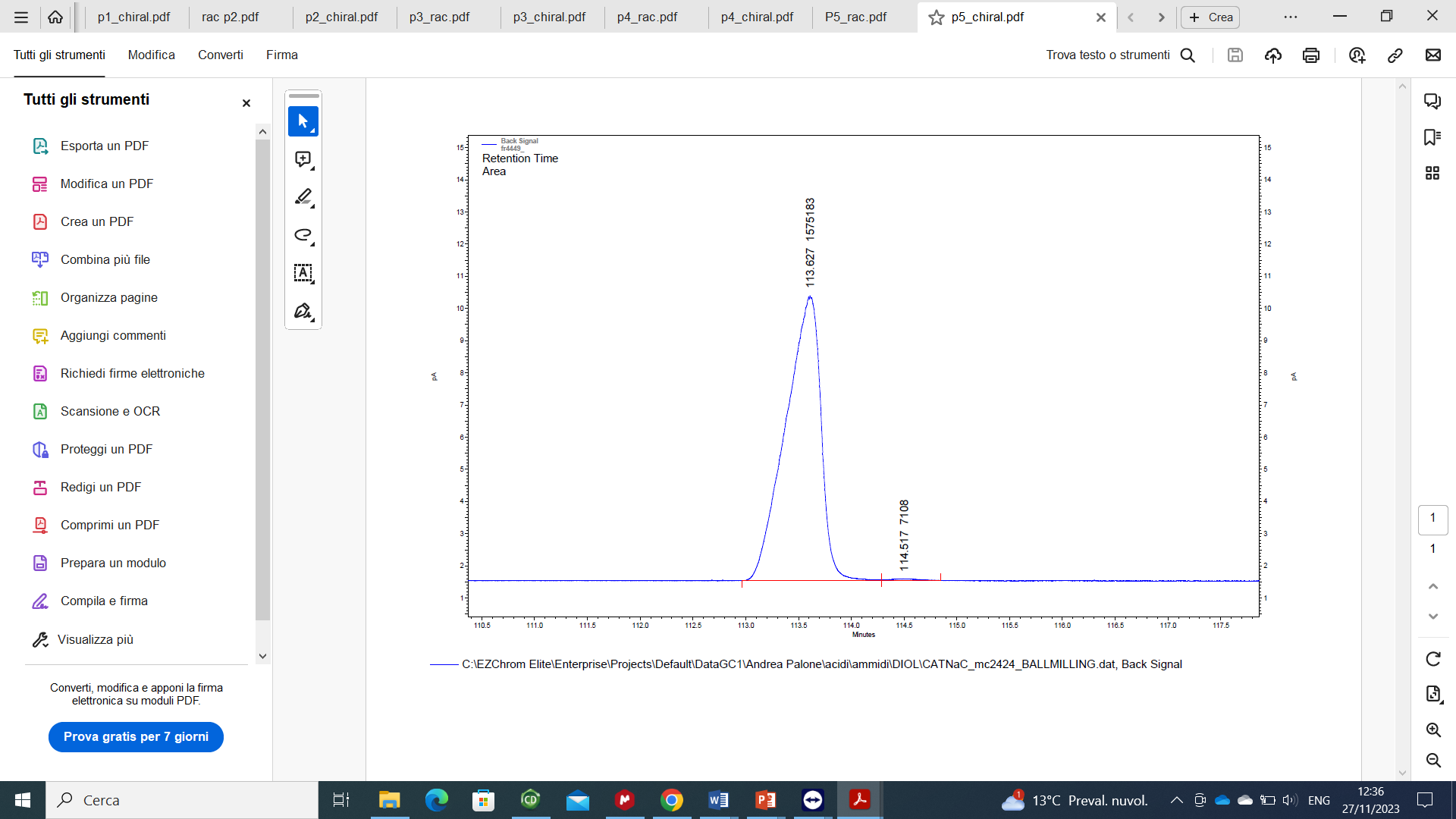


**Rac-P6**


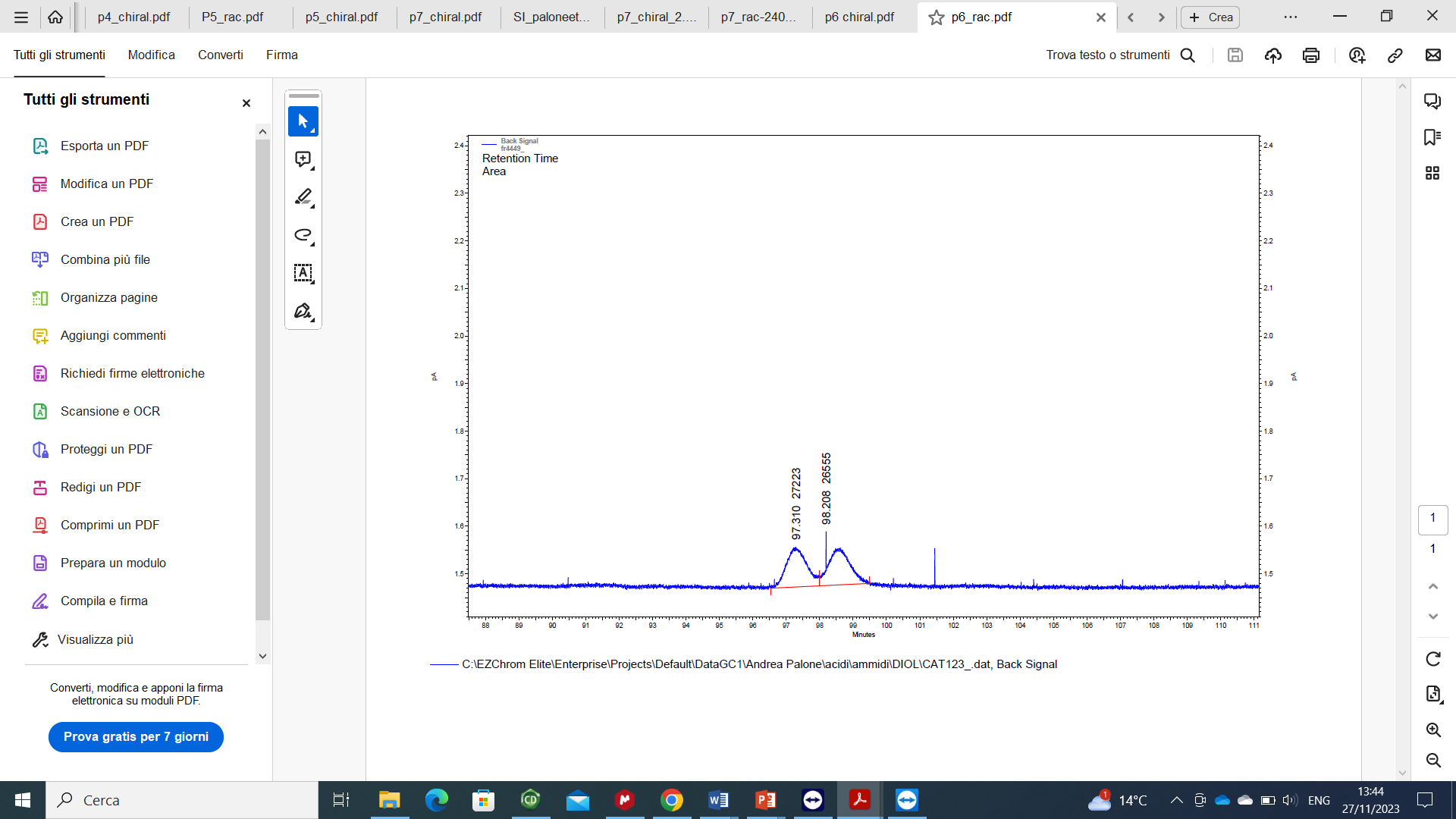


**Chiral-P6**


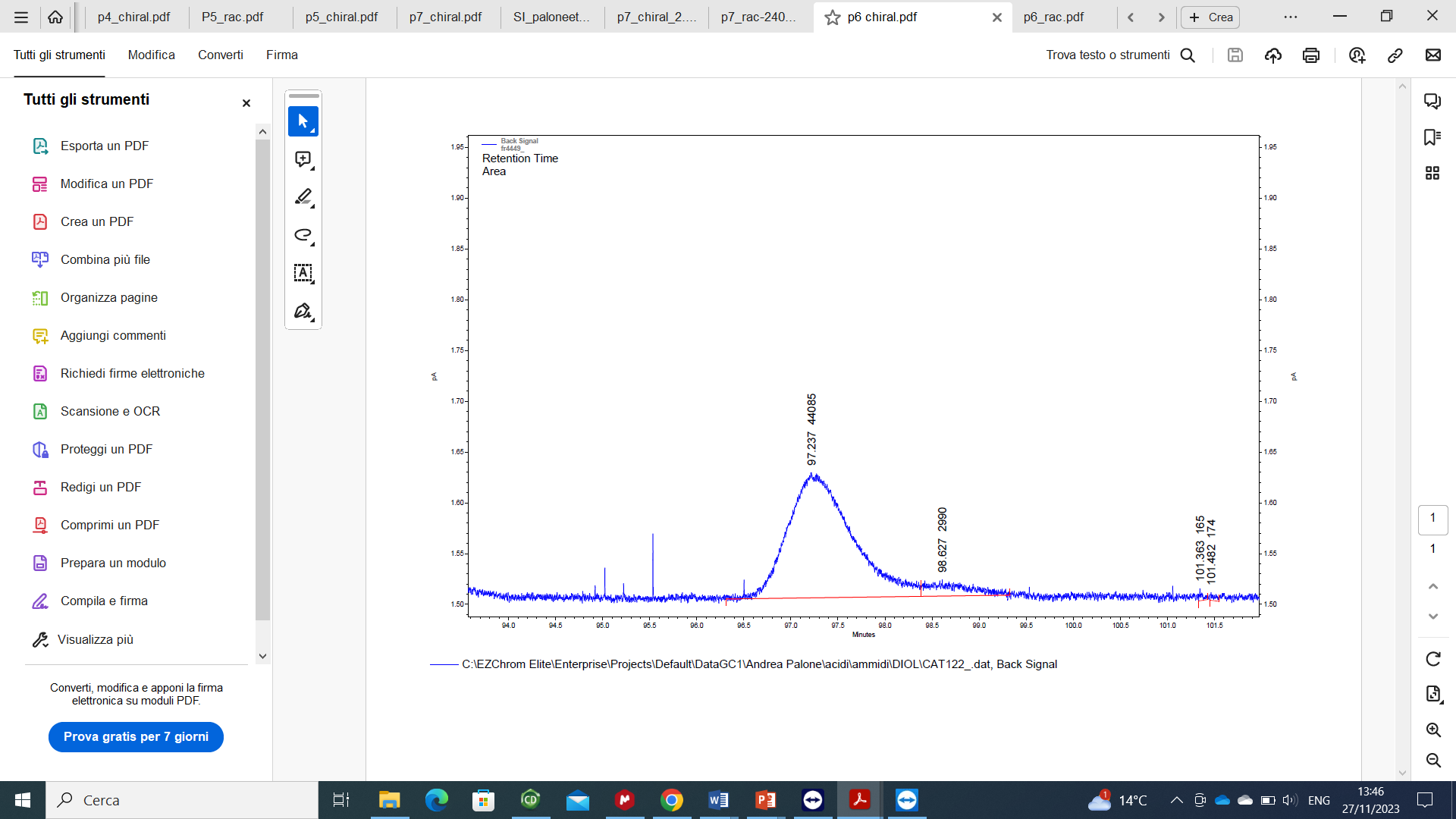


SFC separation conditions: Chiralpack IC-3 (100 × 4.6mm, 3μm), 240.4 nm, CO_2_/MeOH= 80:20, 1.2 mL/min; r.t. (minor) = 1.96 min, r.t. (major) = 2.25 min.

**Rac-P7**


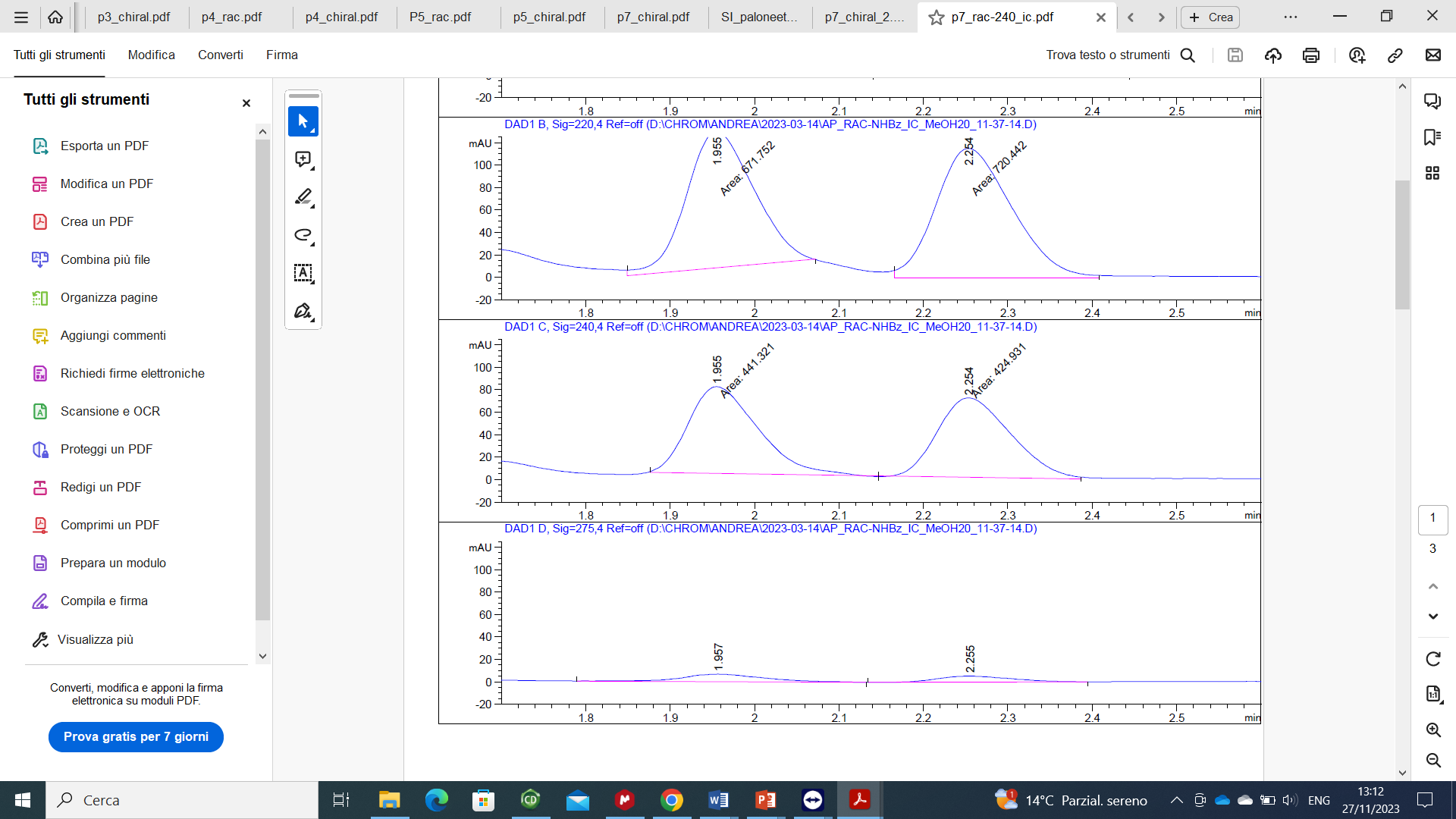


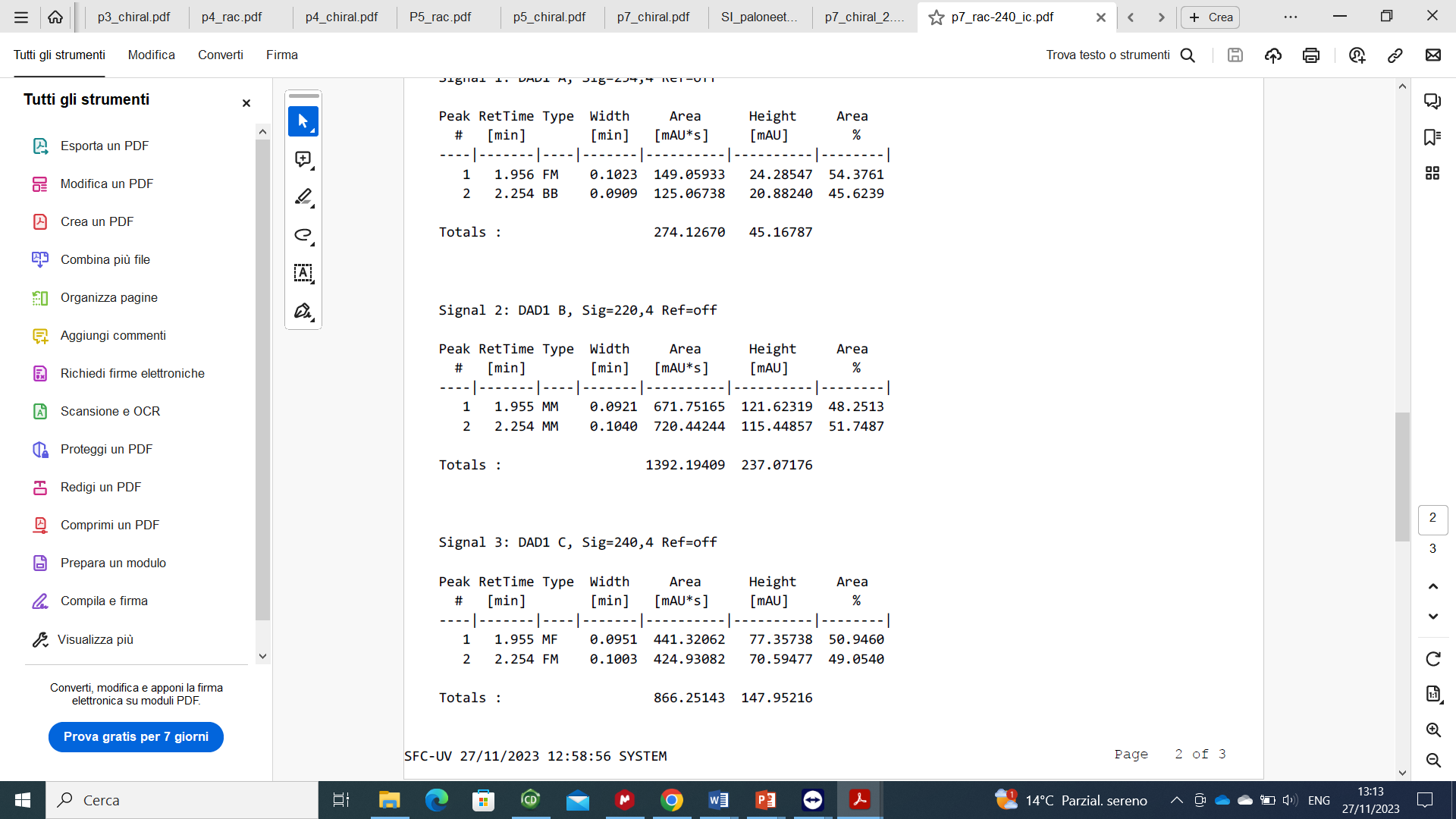


**Chiral-P7**


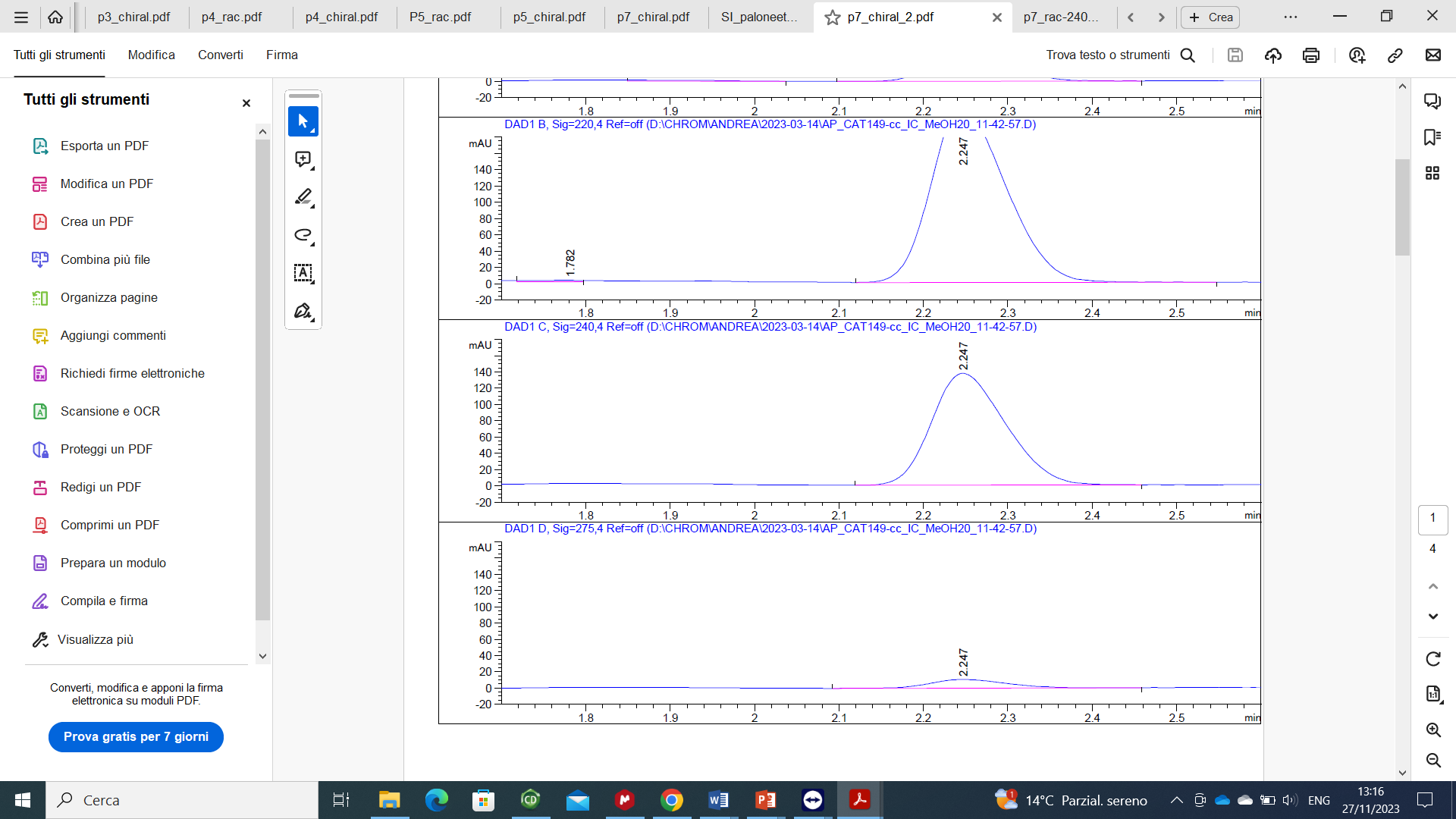

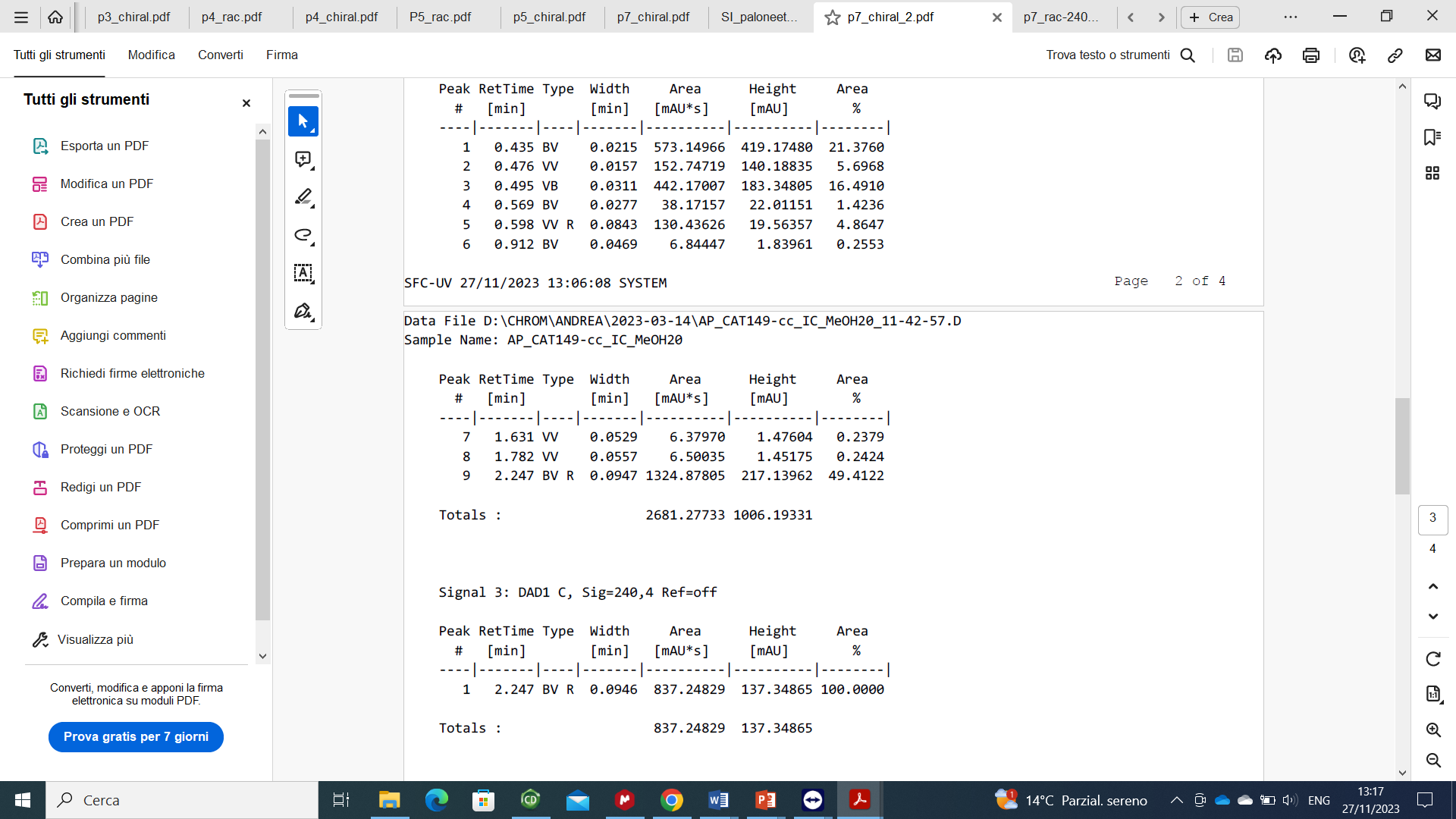


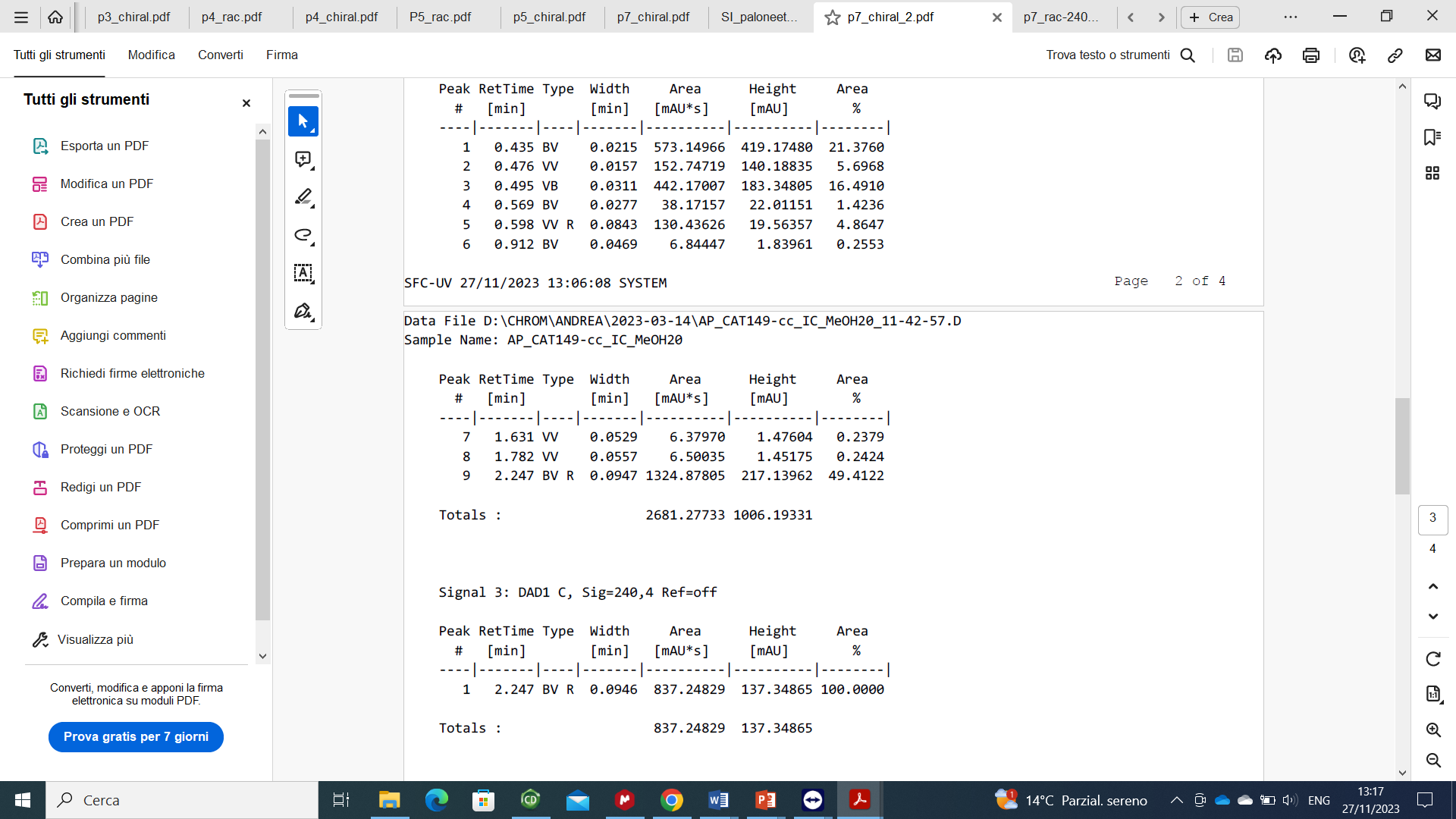


**Rac-P8**


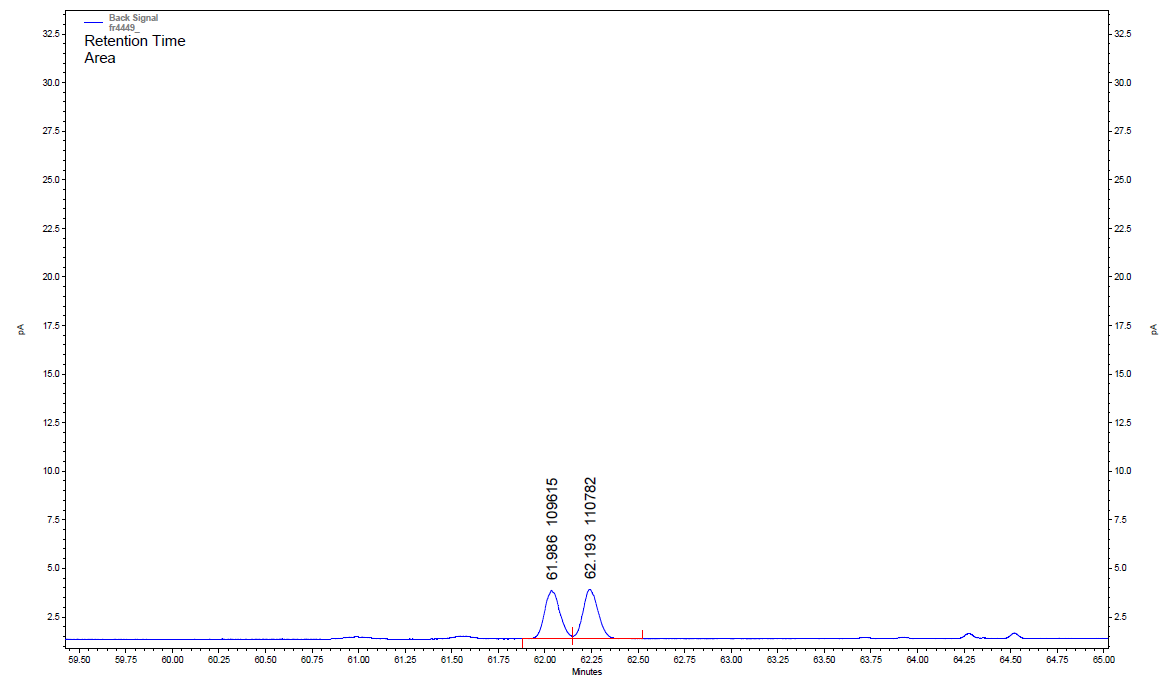


**Chiral-P8**


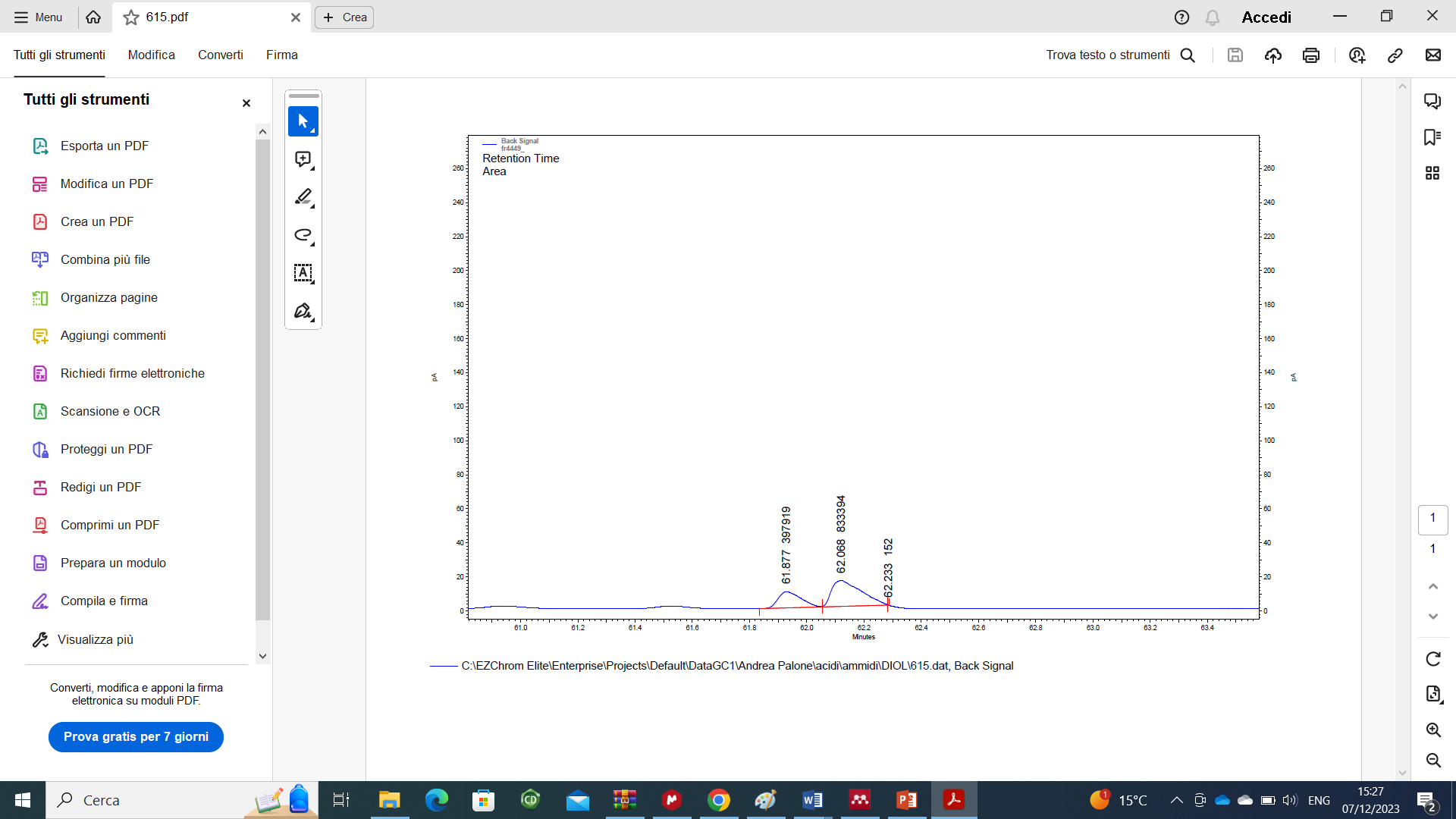


**Rac-P9**


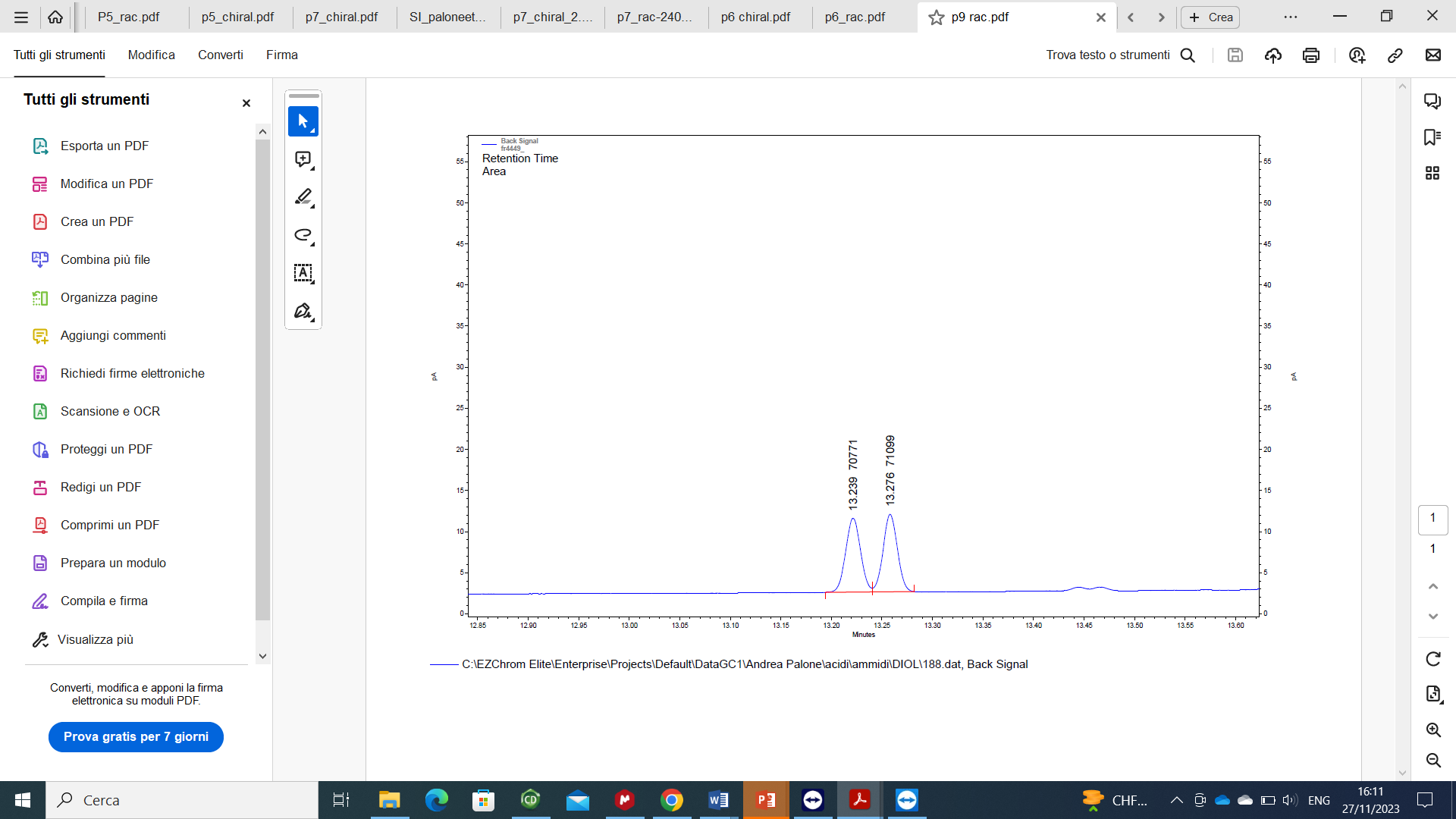


**Chiral-P9**


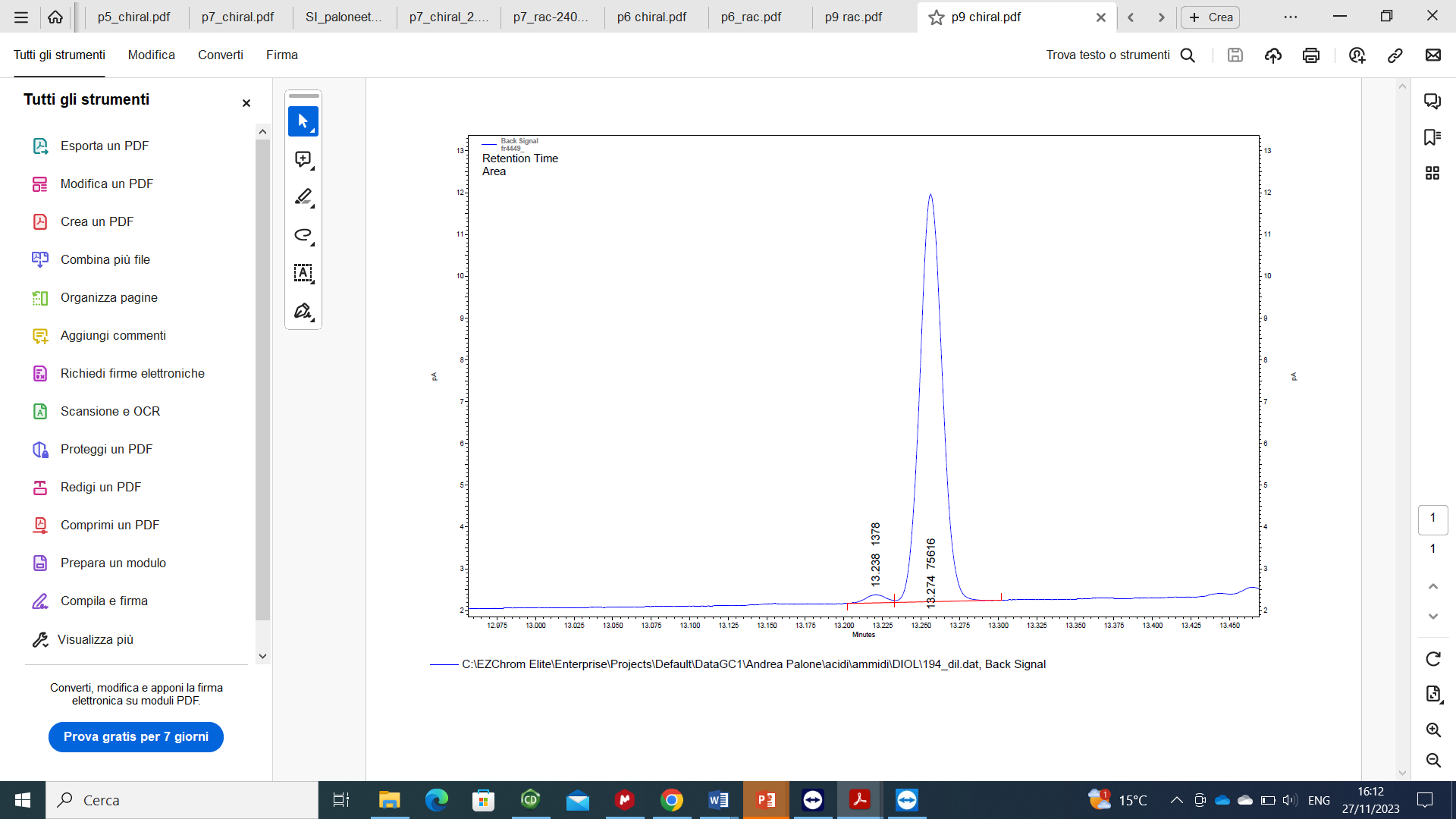


**Rac-P10**


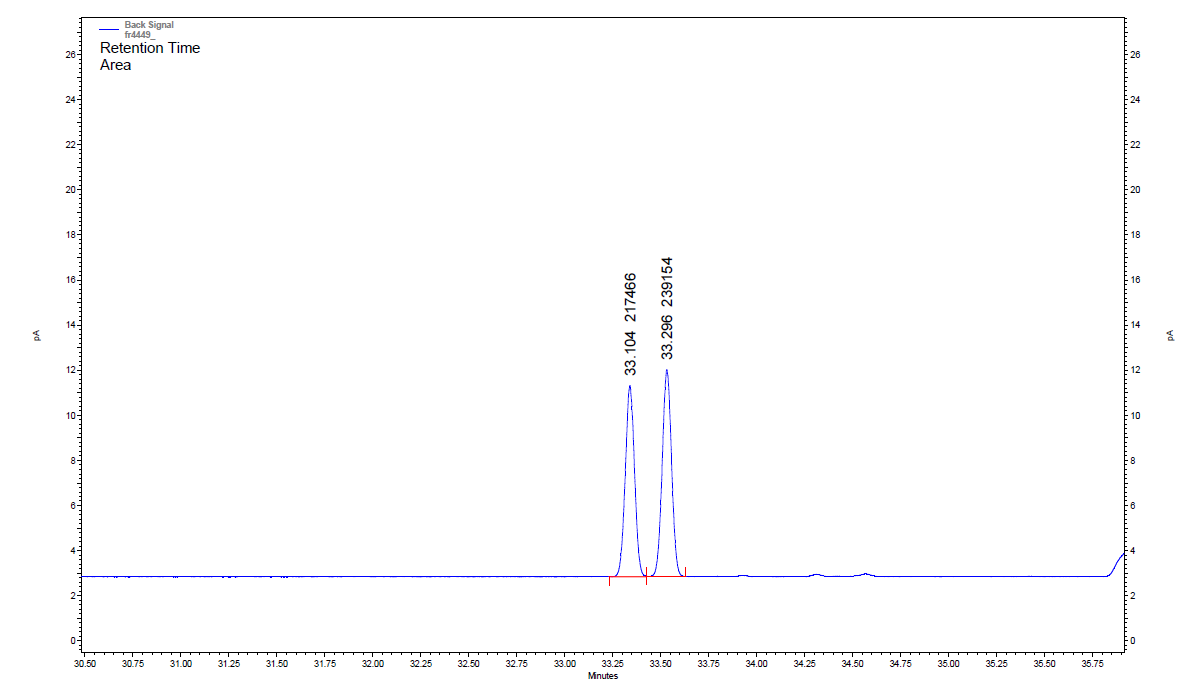


**Chiral-P10**


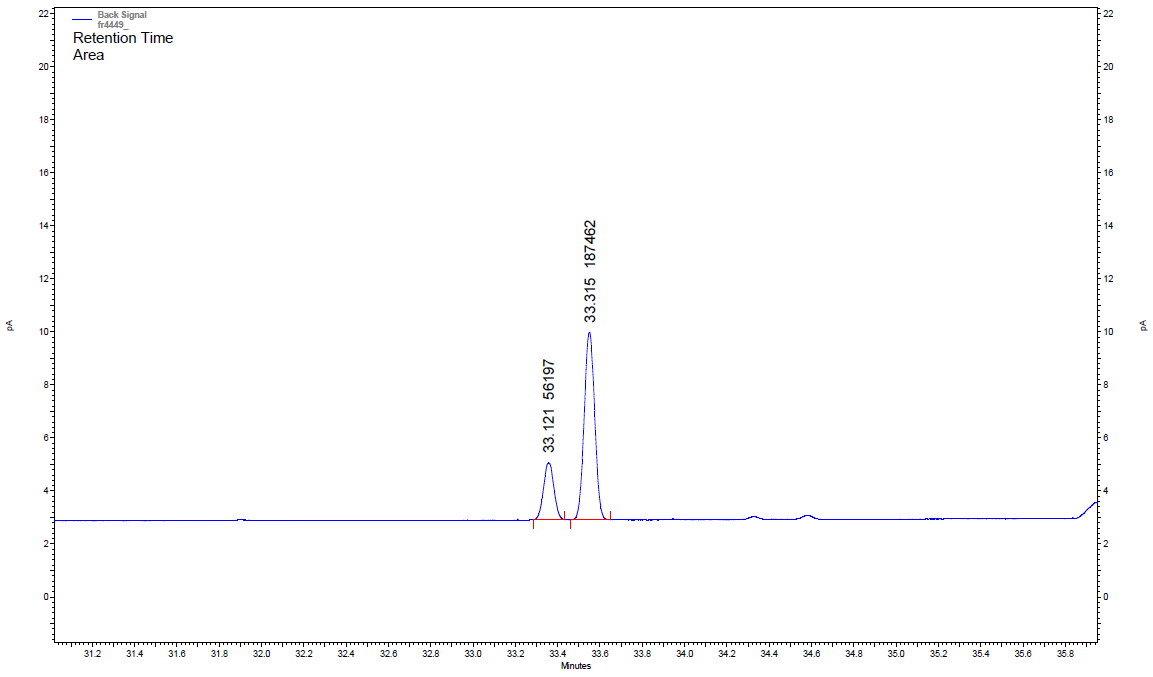


SFC separation conditions: Chiralpack IB-3 (100 × 4.6mm, 3μm), 210.4 nm, CO_2_/ MeOH= 92:8, 1.0 mL/min; r.t. (major) = 1.96 min, r.t. (minor) = 2.22 min.

**Rac-P11**


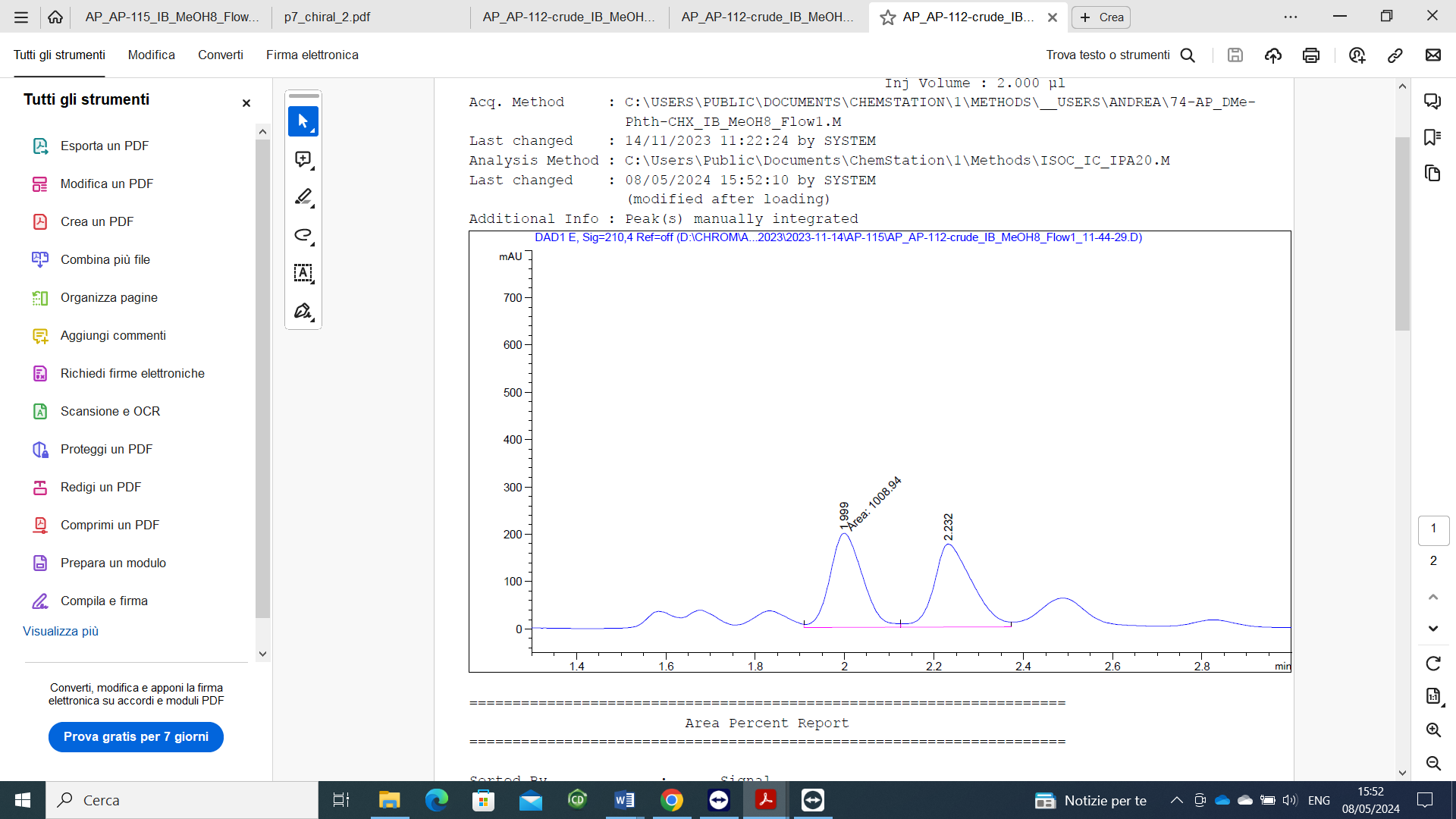


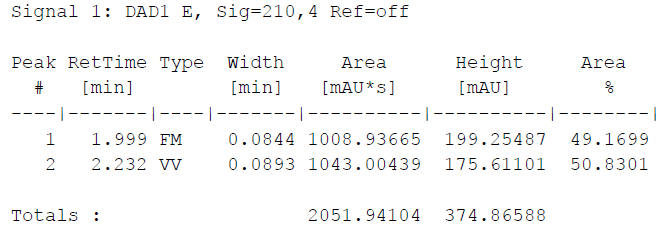


**Chiral-P11**


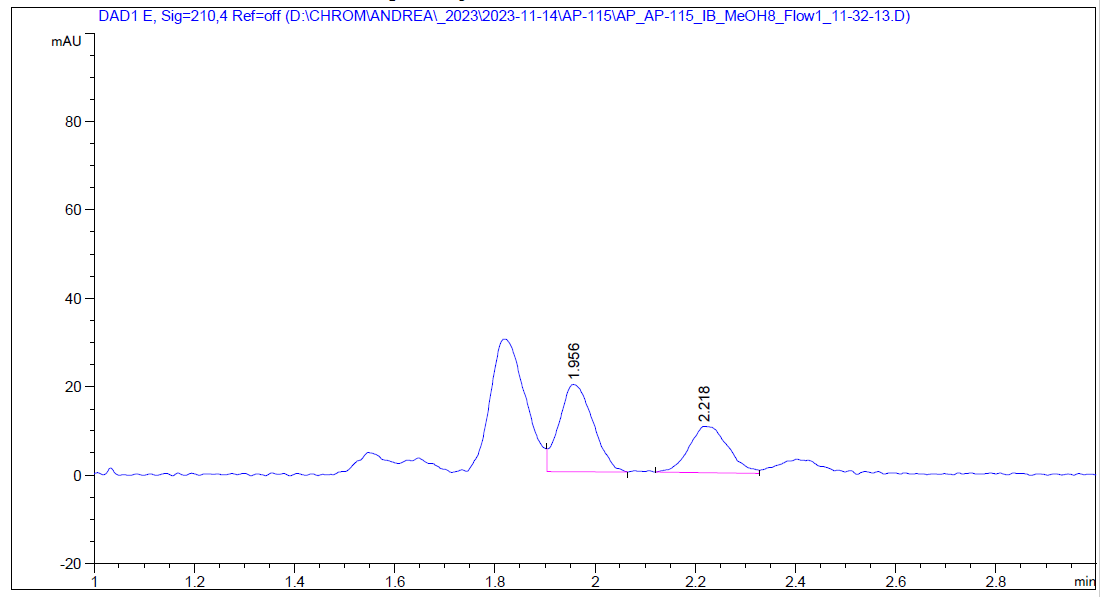


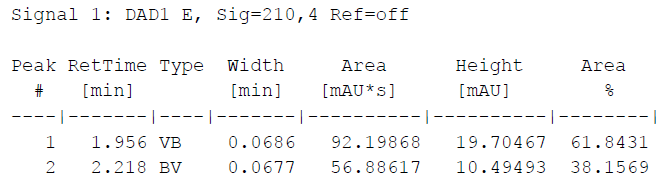


SFC separation conditions: Chiralpack IA-3 (100 × 4.6mm, 3μm), 220.4 nm, CO_2_/ iPA= 80:20, 1.0 mL/min; r.t. (major) = 1.86 min, r.t. (minor) = 2.31 min.

**Rac-P12**


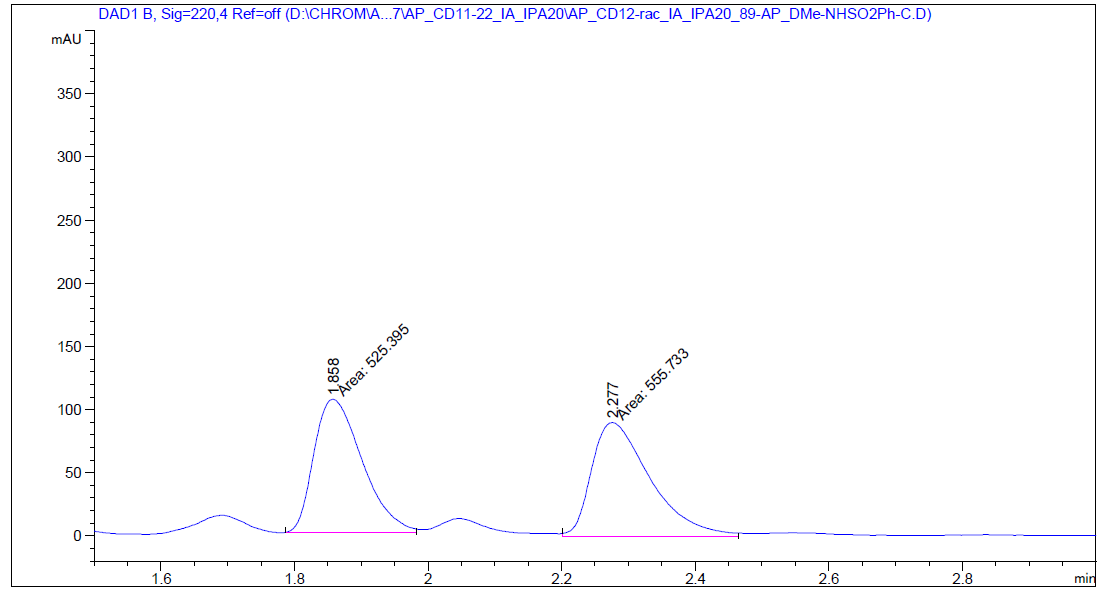


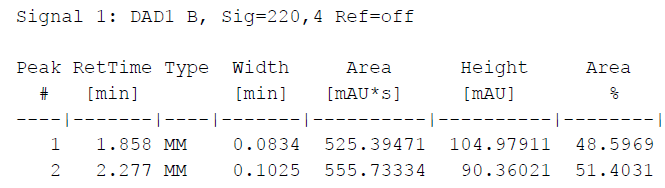


**Chiral-P12**


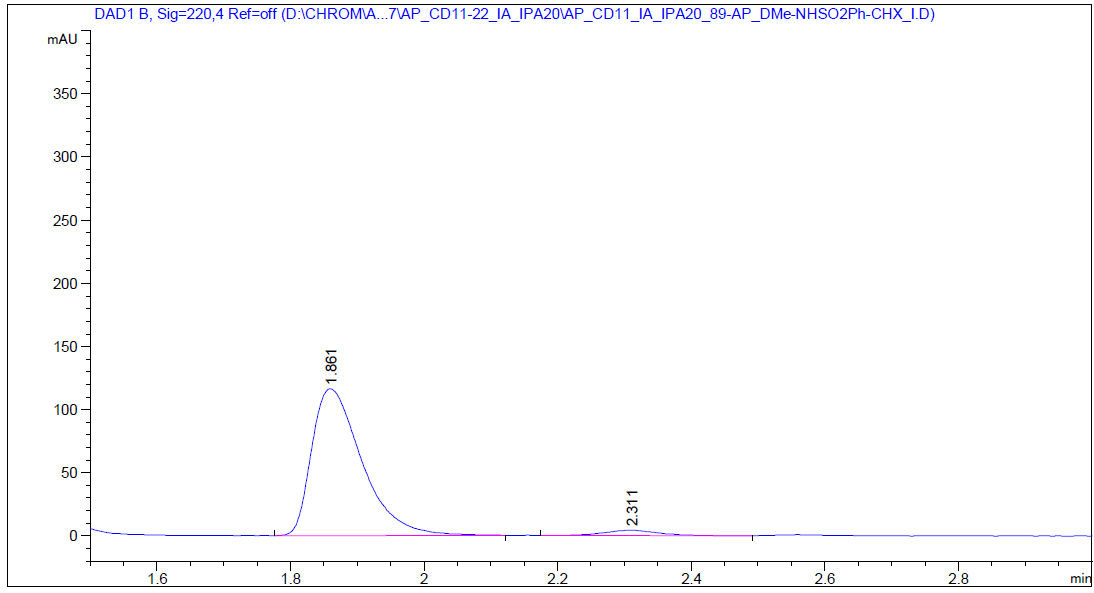


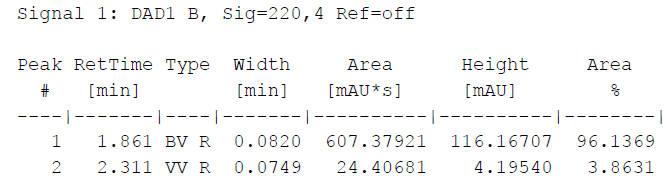


**Rac-P13**


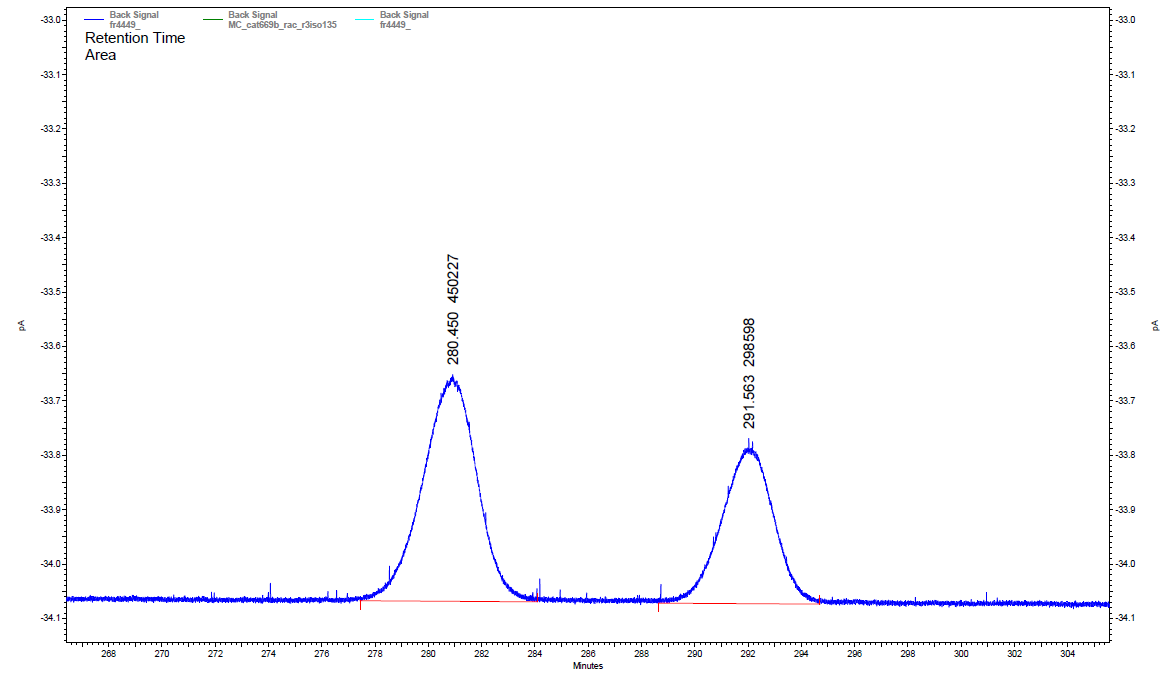


**Chiral-P13**


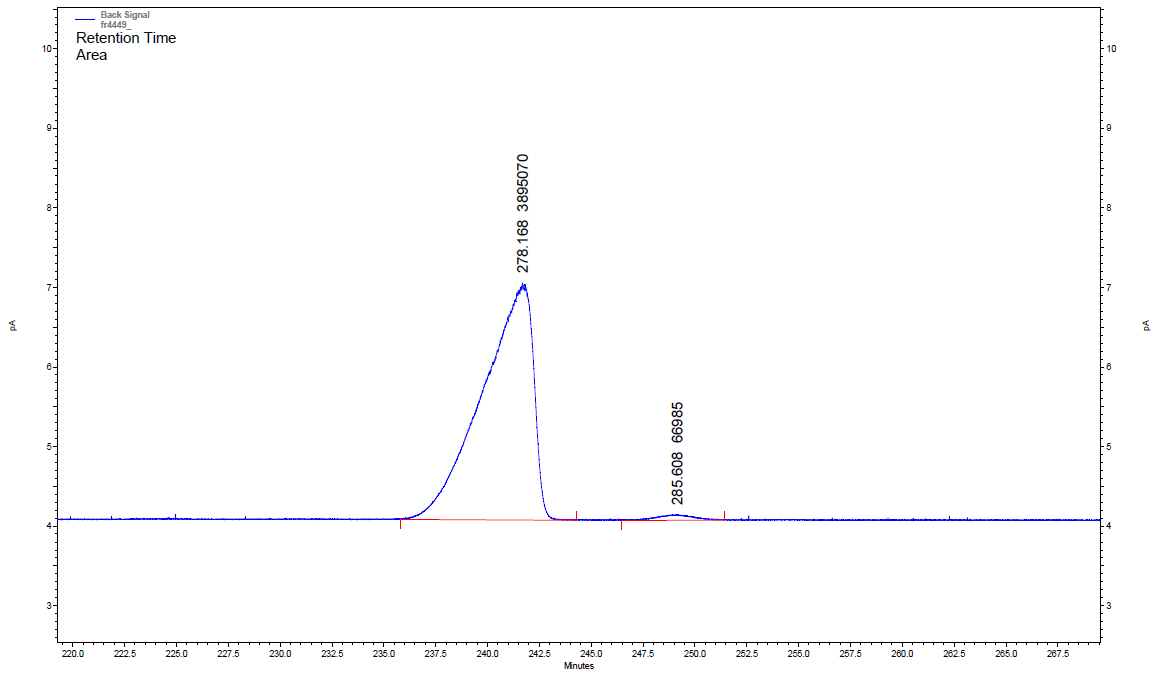


**Rac-P14**


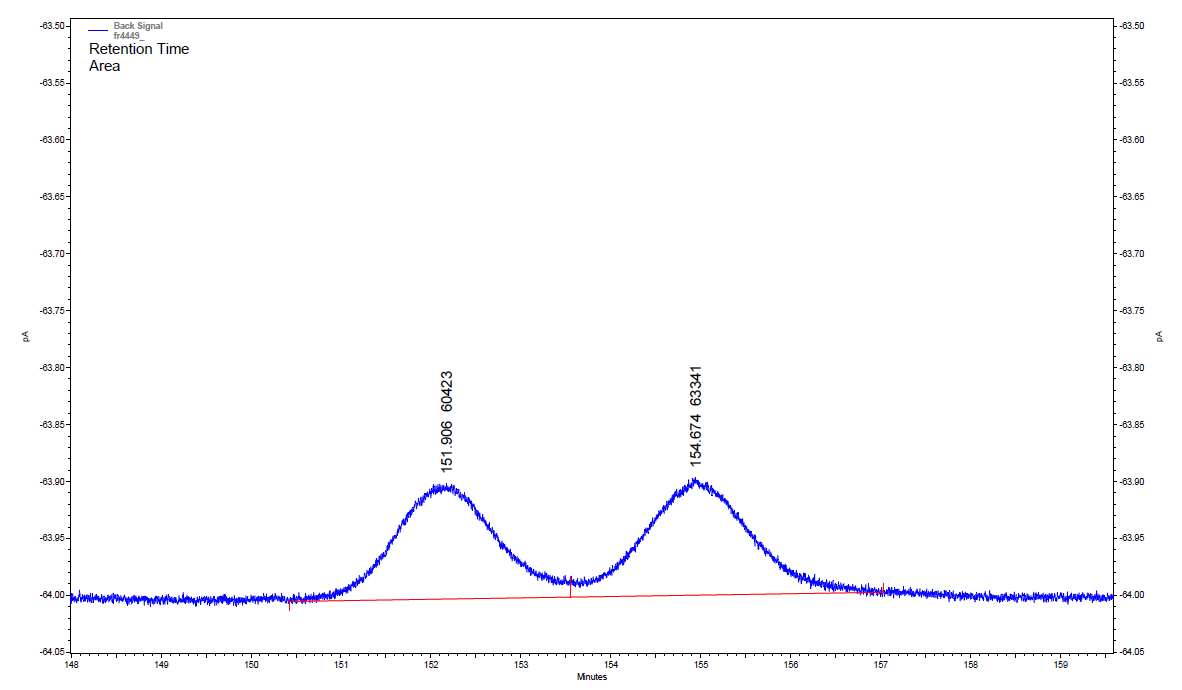


**Chiral-P14**


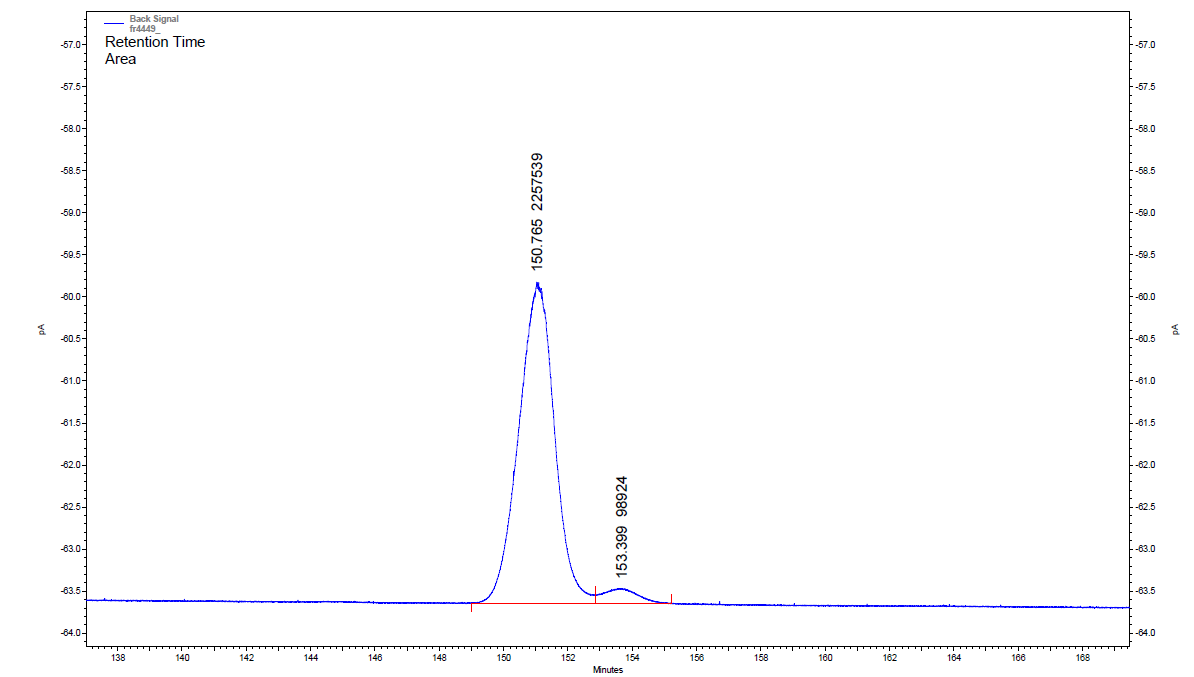


**2.1. References**

[1] K. Usuda, T. Biswas, T. Yamaguchi, Y. Akagi, K. Yasui, M. Uesugi, I. Shimizu, S. Hosokawa, K. Nagasawa, *Chem. Pharm. Bull.* **2016**, *64*, 1190–1195.

[2] Y. Zhao, Y. Wu, P. D. Clercq, M. Vandewalle, P. Maillos, J.-C. Pascal, *Tetrahedron: Asym.* **2000**, *11*, 3887-3900.

[3] M. Milan, M. Bietti, M. Costas, *ACS Cent. Sci.* **2017**, *3*, 196-204.

[4] A. Call, G. Capocasa, A. Palone, L. Vicens, E. Aparicio, N. Choukairi Afailal, N. Siakavaras, M. E. López Saló, M. Bietti, M. Costas, *J. Am. Chem. Soc.* **2023**, *145*, 18094-18103.

[5] M. Galeotti, L. Vicens, M. Salamone, M. Costas, M. Bietti, *J. Am. Chem. Soc.* **2022**, *144*, 7391-7401.

[6] R. V. Ottenbacher, K. P. Bryliakov, E. P. Talsi, *Adv. Synth. Catal.* **2011**, *353*, 885-889.

[7] D. Font, M. Canta, M. Milan, O. Cussó, X. Ribas, R. J. M. K. Gebbink, M. Costas, *Angew. Chem. Int. Ed.* **2016**, *55*, 5776 –5779.

[8] O. Cussó, I. Garcia-Bosch, D. Font, X. Ribas, J. Lloret-Fillol, M. Costas, *Org. Lett.* **2013**, *15*, 6158-6161.

[9] K. C. Nicolaou, Y. H. Lim, J. L. Piper, C. D. Papageorgiou, *J. Am. Chem. Soc.* **2007**, *129*, 4001-4013.

[10] N. Uchida, J. Kuwabara, A. Taketoshi, T. Kanbara, *J. Org. Chem.* **2012**, *77*, 10631-10637.

[11] S. Escayola, N. Bahri-Laleh, A. Poater, *Chem Soc Rev* **2024**, *53*, 853-882.

[12] K. P. Bryliakov, E. P. Talsi, *Coord. Chem. Rev.* **2014**, *276*, 73-96.

[13] L. Falivene, Z. Cao, A. Petta, L. Serra, A. Poater, R. Oliva, V. Scarano, L. Cavallo, *Nat. Chem.* **2019**, *11*, 872-879.

[14] M. J. Frisch, G. W. Trucks, H. B. Schlegel, G. E. Scuseria, M. A. Robb, J. R. Cheeseman, G. Scalmani, V. Barone, G. A. Petersson, H. Nakatsuji, X. Li, M. Caricato, A. V. Marenich, J. Bloino, B. G. Janesko, R. Gomperts, B. Mennucci, H. P. Hratchian, J. V. Ortiz, A. F. Izmaylov, J. L. Sonnenberg, Williams, F. Ding, F. Lipparini, F. Egidi, J. Goings, B. Peng, A. Petrone, T. Henderson, D. Ranasinghe, V. G. Zakrzewski, J. Gao, N. Rega, G. Zheng, W. Liang, M. Hada, M. Ehara, K. Toyota, R. Fukuda, J. Hasegawa, M. Ishida, T. Nakajima, Y. Honda, O. Kitao, H. Nakai, T. Vreven, K. Throssell, J. A. Montgomery Jr., J. E. Peralta, F. Ogliaro, M. J. Bearpark, J. J. Heyd, E. N. Brothers, K. N. Kudin, V. N. Staroverov, T. A. Keith, R. Kobayashi, J. Normand, K. Raghavachari, A. P. Rendell, J. C. Burant, S. S. Iyengar, J. Tomasi, M. Cossi, J. M. Millam, M. Klene, C. Adamo, R. Cammi, J. W. Ochterski, R. L. Martin, K. Morokuma, O. Farkas, J. B. Foresman, D. J. Fox, Wallingford, CT, **2016**.

[15] S. Grimme, S. Ehrlich, L. Goerigk, *J. Comput. Chem.* **2011**, *32*, 1456-1465.

[16] S. Grimme, J. Antony, S. Ehrlich, H. Krieg, *J. Chem. Phys.* **2010**, *132*, 154104-154119.

[17] A. Schäfer, C. Huber, R. Ahlrichs, *J. Chem. Phys.* **1994**, *100*, 5829-5835.

[18] A. V. Marenich, C. J. Cramer, D. G. Truhlar, *J. Phys. Chem. B* **2009**, *113*, 6378-6396.

[19] J. Contreras-Garcia, E. R. Johnson, S. Keinan, R. Chaudret, J. P. Piquemal, D. N. Beratan, W. Yang, *J. Chem. Theory Comput.* **2011**, *7*, 625-632.
